# “High-Throughput Characterization of Region-Specific Mitochondrial Function and Morphology”

**DOI:** 10.1038/s41598-017-05152-z

**Published:** 2017-07-27

**Authors:** Joseph R. Daniele, Daniel J. Esping, Gilbert Garcia, Lee S. Parsons, Edgar A. Arriaga, Andrew Dillin

**Affiliations:** 1 Department of Molecular & Cellular Biology, University of California, Berkeley, Berkeley, CA 94720-3370 USA; 2 Department of Chemistry, University of Minnesota, Minneapolis, MN 55455 USA

## Abstract

The tissue-specific etiology of aging and stress has been elusive due to limitations in data processing of current techniques. Despite that many techniques are high-throughput, they usually use singular features of the data (e.g. whole fluorescence). One technology at the nexus of fluorescence-based screens is large particle flow cytometry (“biosorter”), capable of recording positional fluorescence and object granularity information from many individual live animals. Current processing of biosorter data, however, do not integrate positional information into their analysis and data visualization. Here, we present a bioanalytical platform for the quantification of positional information (“longitudinal profiling”) of *C*. *elegans*, which we posit embodies the benefits of both high-throughput screening and high-resolution microscopy. We show the use of these techniques in (1) characterizing distinct responses of a transcriptional reporter to various stresses in defined anatomical regions, (2) identifying regions of high mitochondrial membrane potential in live animals, (3) monitoring regional mitochondrial activity in aging models and during development, and (4) screening for regulators of muscle mitochondrial dynamics in a high-throughput format. This platform offers a significant improvement in the quality of high-throughput biosorter data analysis and visualization, opening new options for region-specific phenotypic screening of complex physiological phenomena and mitochondrial biology.

## Introduction

Methods to reliably identify causative factors of cellular phenomena at the organismal level have been laborious and time consuming due to limitations in current imaging techniques. Automated robotic systems which use multi-well plates (e.g. cell culture) have increased throughput and enabled the characterization of many cellular responses (e.g. reporter genes): however, these readouts are not always applicable to the biology of an intact animal.

To date, a number of genetic screens have been carried out on the small nematode worm, *C*. *elegans*, using a large particle flow cytometer(“biosorter”)^1–6^, which works very similarly to a conventional flow cytometer. However, the currently available analytical platforms do not utilize the positional data being recorded from each particle, leaving regional information out of the analysis. High resolution microscopy, by contrast, has enabled the tissue-specific measurement of various cellular processes in both live and fixed cells, but it is labor intensive, time consuming, and susceptible to high variability due to small sample size. Moreover, these modern imaging techniques have been optimized for single-celled organisms and tissue culture but do not account for the complexity found in metazoans.

Thus, with the intent to improve the quality and visualization of high-throughput, whole-organism, biosorter data we developed a methodology and freely available software (which we have called the “**L**ongitudinal **A**lignment **M**etabolic **P**rofiler” or “**LAMPro**”) to exploit previously under-used positional information and define key regions of interest in various celluar phenomena (e.g. mitochondrial biology). We posit that this platform embodies advantages of both high-throughput screening and high-resolution microscopy, while also substantially removing artefactual data. LAMPro systematically: (1) aligns and normalizes profiles (which enables “within region” statistical analyses with respect to position along an organism) and (2) performs powerful data visualization to enable identification of subtle differences between animals/strains. While LAMPro can be used for a variety of model organisms, here we demonstrate, with applications to *C*. *elegans* biology, i.e. stress response, bioenergetics, and mitochondrial morphology/dynamics, how LAMPro’s streamlined data analysis and unprecedented data visualization capability can improve the quality and sensitivity of conventional biosorter data.

## Results

Individual organism measurements done by large particle flow cytometry represent the total response per organism^1–3^. Because animals being studied are composed of different cell types, which vary in their spatial distribution and contribution to this “representative” value, such a portrayal may inadequately represent the biological process of interest in a given screening study. Our methodology and software, LAMPro, can accommodate for the positional complexity and variability found in multi-celled metazoa via a three-step process: (1) non-ideal longitudinal profiles are excluded, which reduces data variability, (2) individual profiles are aligned, allowing for positional statistics to be done on large populations, and (3) data can be effectively visualized and quantified. Thus, through the study of mitochondrial stress, bioenergetics in development and longevity, and morphology/dynamics, we outline the main features of LAMPro and its ability to improve the quality of longitudinal data analysis and visualization.

### Object Orientation for Tissue-Specific Analysis

**Figure 1:**
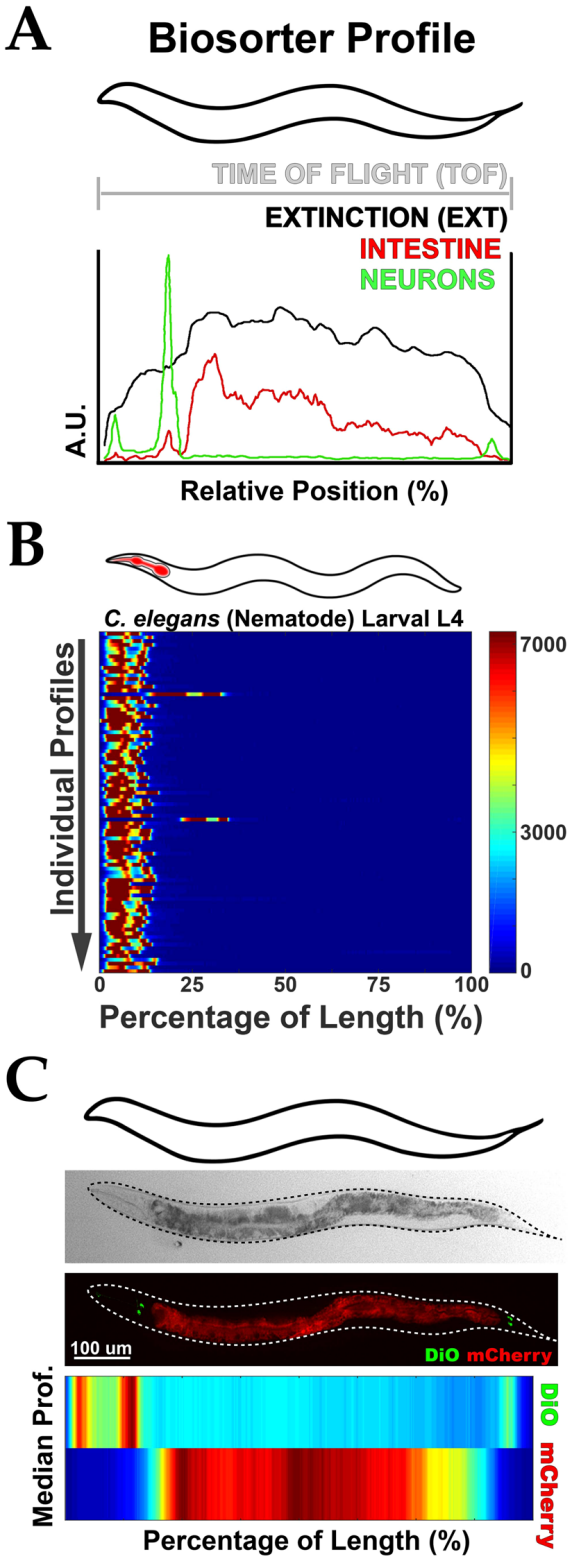
Algorithm to align biosorter data and derive positional information. (**A**) Representative profile for a single nematode displaying longitudinal information in: Extinction (Black) and tissue-specific fluorescent signals (Green = Neurons, Red = Intestine). Anterior is to the left. “A.U.” = Arbitrary Units. (**B,C,E,F**) Illustration of orientation algorithm devised to align animals. Example shows individual profiles (Y axis) represented as in (**A**) with signal intensity plotted as a function of the animal’s length (X axis). Tissues have been labeled (red in cartoons above) to enable orientation of nematodes (**B**). Data is shown in a heat map with Red as the most signal and Blue as the least. (**D**) Demonstration of an average profile algorithm following alignment. (Top) Representative micrographs of transgenic worms analyzed below with intestine (Red, *vha-6p* *::mCherry::HDEL*) and sensory neurons (DiO, Green) fluorescently labeled. Worms are oriented with their anterior to the left. Scale = 200 µm. (Bottom) Median profile from 100+ animals in the Red (intestine) and Green (neurons) channels. Data is shown in a heat map with Red as the highest intensity signal and Blue, the lowest. All animals are positioned with the anterior side to the left. Plots are representative of three biological replicates and derived from *n* ≥ 50 organisms. Sample size (*n*) for all experiments can be found in the Supplementary Table. L.A. Daniele provided the illustrations.

In large particle flow cytometry, optical and fluorescent intensity values are recorded as objects move past a laser detector. The length of an object is recorded as the “time of flight” (TOF, upper gray bar, Fig. 1A) and each relative time represents a different longitudinal position. For a *C*. *elegans* expressing fluorescent, tissue-specific reporters, longitudinal measurements can be represented in an optical profile, which demonstrates the location and intensity of all parameters at each position. For example, green fluorescently labeled sensory neurons in the far anterior and posterior (green trace, Fig. 1A) and red fluorescently labeled intestine (red trace, Fig. 1A). Such measurements also include a longitudinal representation of the nematode’s “width” (granularity) plotted by “extinction” (EXT, black trace, Fig. 1A). Furthermore, the spatial resolution of a longitudinal profile is ~12 µm (or ~2% of a 620 µm long larval L4 *C*. *elegans*), which can distinguish individual neural clusters and pharyngeal bulbs, gut cells, and other fine features within a single animal.

Through our alignment and length-normalization algorithms, LAMPro software is able to align asymmetrically-distributed tissues from a broad range of longitudinal profile data (see Figs 1B, S1, S2B and S2C, and Supplementary Information, *LAMPro Software Details and Specifications*). Once aligned and normalized, the points along the length of each individual animal’s optical trace (in each channel recorded) allows for representative sample-population statistics (e.g. median signal intensity, Median Absolute Deviation (MAD)) to be rendered digitally as heat maps for any recorded channel (e.g. fluorescence in three channels (green, yellow, and red) and granularity/width (extinction, EXT)). An example of a heat map output is shown for the medians of sensory neurons (green) and intestine (red) at the bottom of Fig. 1C. This profile is in perfect agreement with a representative micrograph of one nematode with the same labels (Fig. 1C, middle panel). Thus, following alignment and length-normalization, the “median profile” can be used to represent the longitudinal fluorescence pattern of different tissues for a population of animals, such as neurons and intestine.

**Figure 2:**
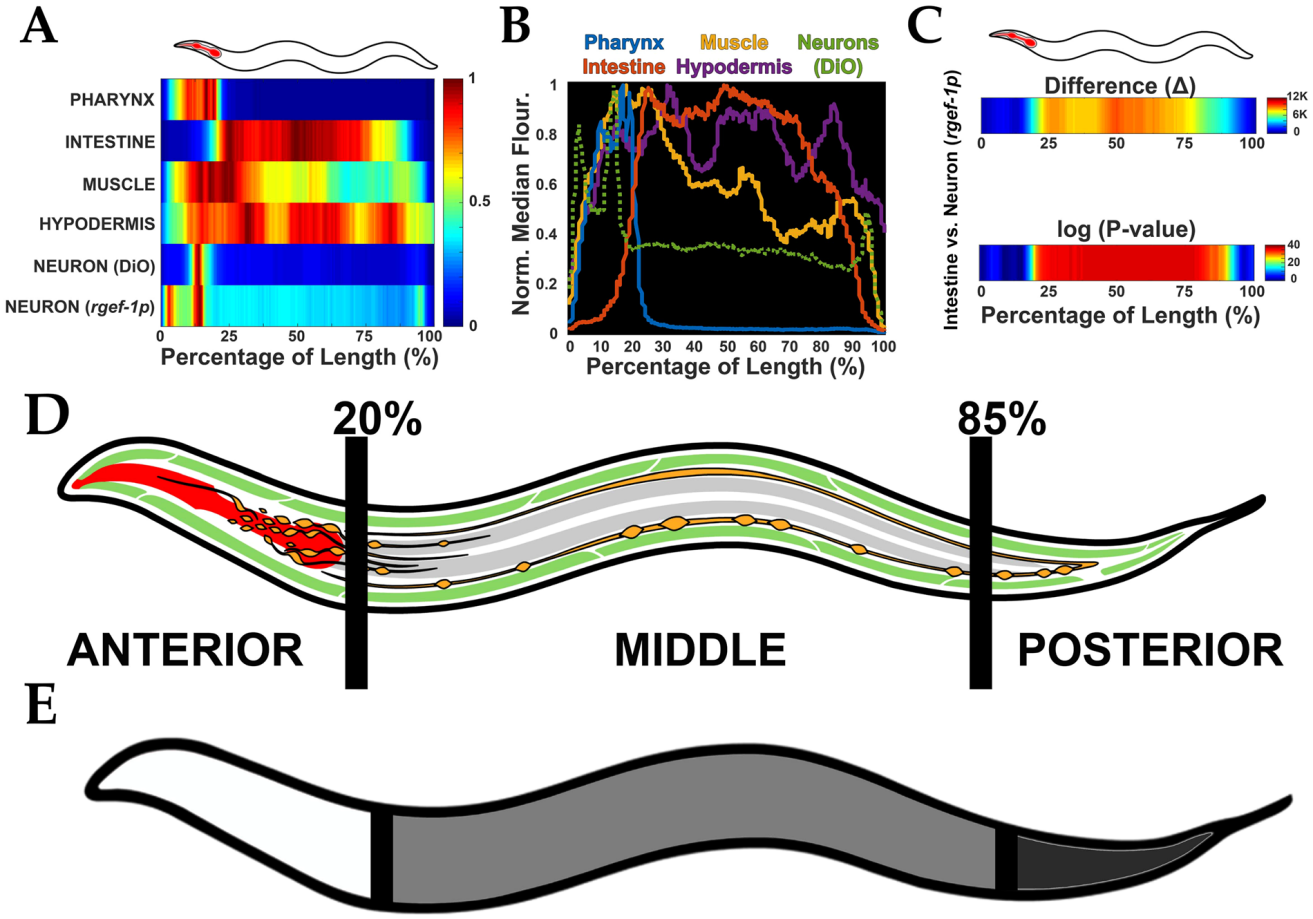
Characterization of tissue-specific expression and derivation of “regions of interest”. (**A**) Median profiles derived from various *C*. *elegans* transgenic lines expressing a fluorescent marker in each respective tissue. Animals were concurrently live-stained with DiO or DiI (which labels sensory neurons) to enable consistent orientation across strains. (**B**) Graphical representation of transgenic lines in (**A**) to demonstrate resolution of individual tissues as a function of the animal’s length. (**C**) Difference (Δ) plot (top) derived from average profiles in (**A**) and significance plot (bottom) based on these differences. For significance plots “−1.3-log_10_(*P-value*)” was plotted which distinguished regions of significance by Wilcoxon Rank Sum Test. A value ≥ 0 is equilvalent to *P* ≤ 0.05. (**D**) Anatomical representation of various *C*. *elegans* tissues and the derived “regions of interest” from them (e.g. Anterior: 0–20%, Middle: 20–85%, and Posterior: 85–100%). Pharynx in red, neurons in orange, muscle in green, and intestine in grey. (**E**) Simplified schematic of these three regions, Anterior (white), Middle (grey), and Posterior (dark grey) to demonstrate the relative cutoff points of these various areas of interest. All animals are positioned with the anterior side to the left. Plots are representative of three biological replicates and derived from *n* ≥ 50 organisms. Sample size (*n*) for all experiments and exact *P-values* can be found in the Supplementary Table. L.A. Daniele provided the illustrations.

LAMPro can also represent the longitudinal distribution of tissue-specific reporters in most of the nematode tissues as shown by the maps of “median” profiles shown for pharynx, intestine, muscle, hypodermis, and neurons (Fig. 2A). Such profiles were aligned using DiO or DiI (Figure S2B and S2C, respectively) and recapitulated with high fidelity the expression patterns seen via microscopy (Figure S2A). An overlay of “median” profiles also reveals boundary patterns for each tissue (Fig. 2B), and median absolute deviation (MAD) for longitudinal data points shows that there is consistency in alignment as seen by the low signal variability outside of the observed tissue-specific borders for each corresponding median profile (Figure S2D). Of note, all experiments in this manuscript used animals that were staged prior to germline development to eliminate variability in egg number and germline expansion which might affect the spatial patterning of adjacent tissues (Figure S2E,F). Together, these observations illustrate the capability of LAMPro to represent a wide range of tissue-specific reporters in the *C*. *elegans* model.

In comparisons of two reporters, a difference profile and a statistical significance profile were essential to ascertain the region where disparities exist. For instance, we can represent region-specific “differences” between a pan-neuronal reporter and an intestinal reporter (Fig. 2C, **top**) and then test for their statistical significance (Fig. 2C, **bottom**). Comparison of differences in various tissue regions (Figs 2A,B, and S2D) also provided a spatial reference to define three regions of interest (ROI) and their particular tissue composition (Fig. 2D and E). These ROI are the “Anterior” (0% to 20%, included neurons, pharynx, and high expression of hypodermis and muscle markers), the “Middle” (20% to 85%, included mainly intestine, vulval muscle, and a small number of neurons that run laterally), and the “Posterior” regions (85–100%, mainly tail muscle and tail neurons e.g. pre-anal, dorsorectal, and lumbar ganglia). Thus, LAMPro software can “landmark” regions of interest within profiles, with defined tissue compositions, and compare fluorescence traces in live animals.

### Quantification of transcriptional reporters and “regional” significance testing

*C*. *elegans* is an excellent model to explore mechanisms by which mitochondria respond to proteotoxic stress (e.g. mitochondrial unfolded protein response, UPR^mt^)^1, 7–10^. By coupling the transcriptional activity of mitochondrial chaperone genes, *hsp-6* and *hsp-60*, to GFP production, e.g. *hsp-6p* *::gfp* and *hsp-60p*, several groups have identified different mechanisms by which the UPR^mt^ is activated^1, 7, 8, 10^. The current method for comparing UPR^mt^ reporter activity, however, uses the “normalized” total green fluorescence of each profile (one data point per animal) and then plots the descriptive statistics for each data set (Figure S3A). This is inadequate if tissues and cells have differential sensitivity to UPR^mt^ activation, such as that observed in intestinal cells^1, 3^. Also, identification of statistically significant, region-specific, differences would be more tedious to acquire using microscopy alone (Figure S3B). Using LAMPro, we investigated the importance of comparing spatial distribution and intensity of UPR^mt^ induction (via the *hsp-6p* reporter) of the following previously identified challenges: [1] disrupting the formation of electron transport chain (ETC) super-complexes (*cco-1* RNAi, complex IV subunit), [2] removal of a protease that monitors translation and protein folding in the mitochondrial inner membrane (*spg-7* RNAi, m-AAA protease), [3] inhibition of mitochondrial protein translation (*mrps-5* RNAi, 28S ribosomal subunit S5), and [4] inhibition of mitochondrial protein import into the matrix (*tim-17* RNAi, a translocase in the inner mitochondrial membrane).

**Figure 3:**
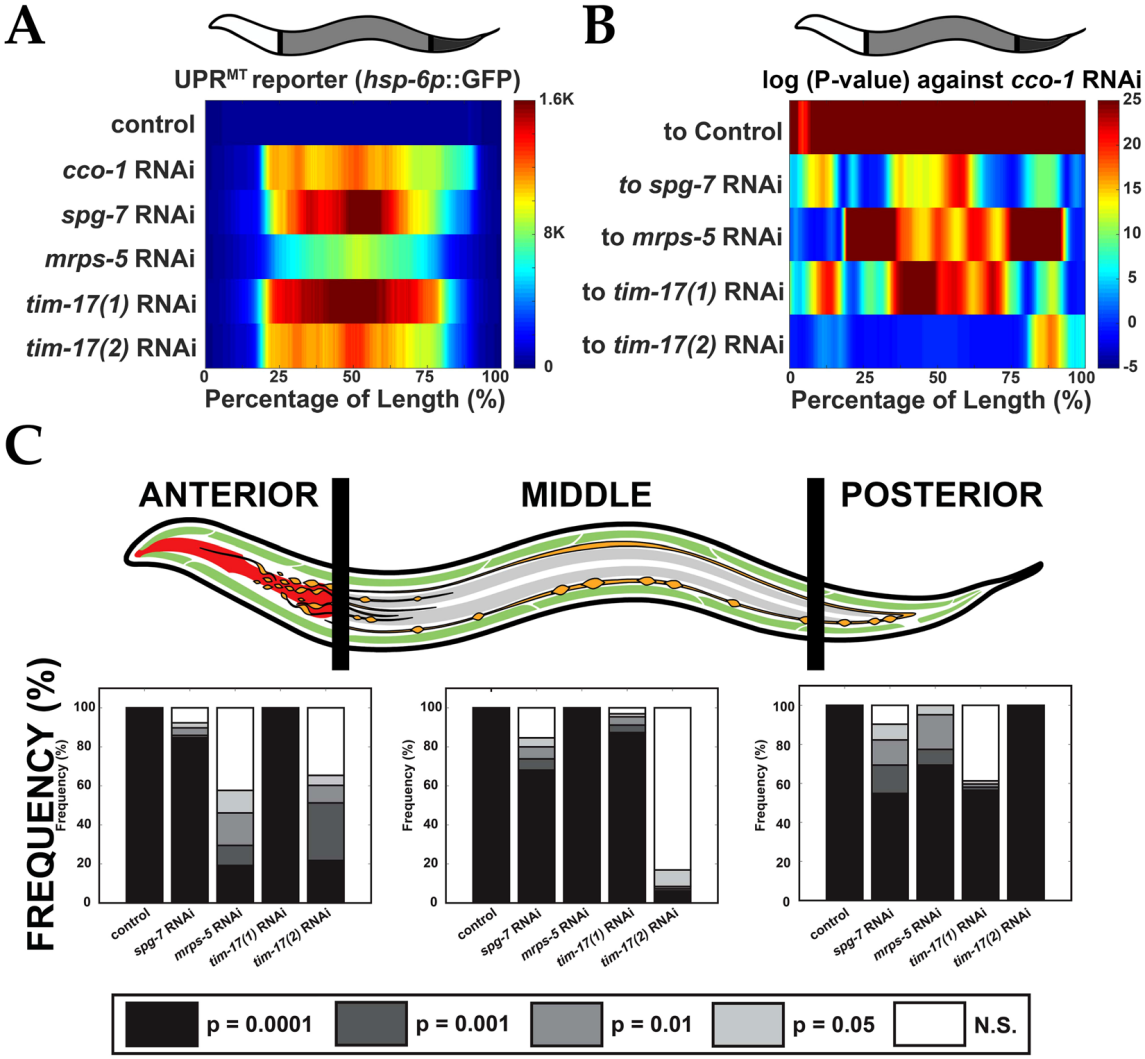
Longitudinal quantification of transcriptional reporter. (**A**) Median profiles of UPR^mt^ after induction of the mitochondrial unfolded protein response (UPR^mt^) by various RNAi treatments. (**B**) Significance plot (log_10_(*P-value*)) derived from median profiles in (**A**) comparing all profiles to “*cco-1* RNAi” (downregulation of Complex I). For significance plots a value ≥ 0 is equivalent to *P* ≤ 0.05 by Wilcoxon Rank Sum Test. (**C**) Frequency plots for the percentage of bits in each region of the worm (left) that correspond to varying degrees of significance by Wilcoxon Ran Sum (white = not significant “N.S.”; light grey = *P* < 0.05; medium grey = *P* < 0.01; dark grey = *P* < 0.001; black = *P* < 0.0001). Comparisons are made for each of these regions against “*cco-1* RNAi”. All animals are positioned with the anterior side to the left. Plots are representative of three biological replicates and derived from *n* ≥ 50 organisms. Sample size (*n*) for all experiments and exact *P-values* can be found in the Supplementary Table. L.A. Daniele provided the illustrations.

The reporter activation profiles between *cco-1* and *tim-17*(*2*) RNAi were very similar to each other, but the UPR^mt^ activation profiles under *spg-7* and *mrps-5* RNAi were quite different from each other (see median profiles, respective variability profiles, and signal saturation plots in Figs 3A, S3C, and S3D–F, respectively). Furthermore, a systematic comparison using heat maps to visualize differences between *cco-1* RNAi and all other conditions (Fig. 3B), showed that the reporter expression under *cco-1* or *tim-17*(*2*) RNAi was statistically identical along the profiles, while comparison of *cco-1* RNAi to *spg-7, mrps-5*, and *tim-17*(*1*) displayed regions that differed significantly (Fig. 3B). To further explore region specific changes, we used the three ROIs defined in Fig. 2D,E (“Anterior”, “Middle”, and “Posterior”) to measure the proportion of significance tiers in Fig. 3C for each ROI (e.g. Anterior) against “*cco-1* RNAi”. Thus, longitudinal profiling made it possible to discern even minor differences in reporter expression under *cco-1* or *tim-17*(*2*) RNAi (Fig. 3B,C). In a more screen-able context, when little or nothing is known about a phenotype, the ability to make statistically significant regional comparisons (“Anterior”, “Middle”, and “Posterior”) in positional UPR^mt^ activation quickly and effectively enabled the identification of the most biologically relevant areas/tissues affected.

### Functional Measurement of Mitochondrial Membrane Potential (ΔΨ)

**Figure 4:**
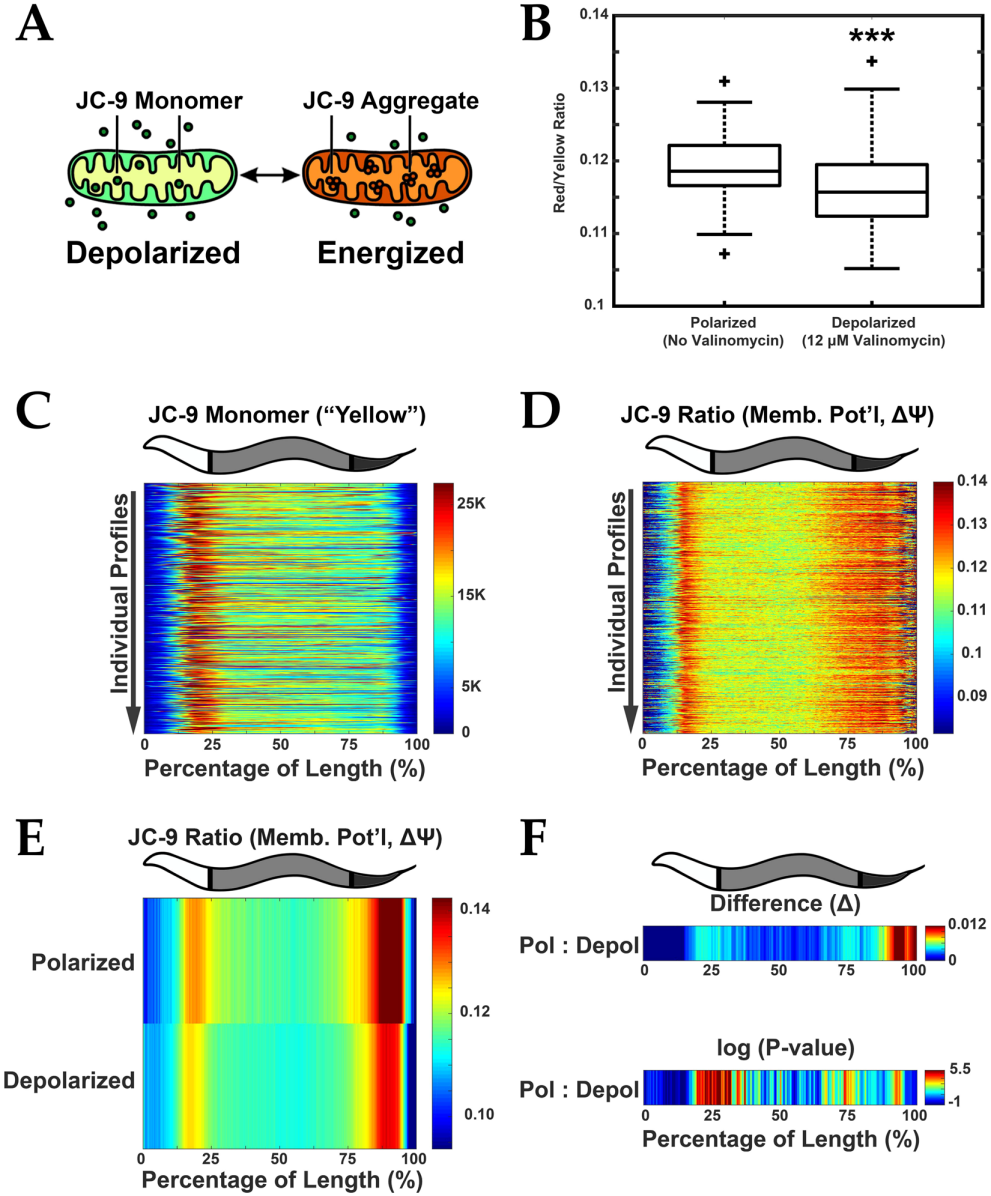
Functional analysis of mitochondrial membrane potential. (**A**) JC-9 is a mitochondrial (Mito) dye that exists in a monomeric (yellow fluorescent) state but forms red fluorescent aggregates when mitochondria are polarized. Red fluorescence increases relative to an increase in mitochondria membrane potential (Δψ). The ratio of Aggregate (Red) to Monomer (Yellow) signal thus approximates the magnitude of membrane potential. (**B**) Ratio of Aggregate to Monomer fluorescence decreases upon depolarization with 12 µM valinomycin indicating a reduction in membrane potential (Δψ). ***P < 0.0001. (**C**) Demonstration of orientation algorithm showing individual profiles (Y axis) represented with signal intensity plotted as a function of the animal’s length (X axis). Yellow (monomeric JC-9) fluorescence is shown which enables orientation in young adult *C*. *elegans*. (**D**) JC-9 ratio (mitochondrial Δψ) for these corresponding profiles is also plotted. Data is shown in a heat map with Red as the most signal and Blue the least. (**E**) Median profiles from animals in (**B**) plotting JC-9 (Aggregate/Monomer) ratio when mitochondria are in the polarized and depolarized state. (**F**) Difference (Δ) plot (top) and significance plot (bottom) derived from median profiles in (**E**) comparing polarized to depolarized conditions. For significance plots a value ≥ 0 is equivalent to *P* ≤ 0.05 by Wilcoxon Rank Sum. All animals are positioned with the anterior side to the left. Plots are representative of three biological replicates and derived from *n* ≥ 50 organisms. Sample size (*n*) and *P-values* can be found in the Supplementary Table. L.A. Daniele provided the illustrations for Part A originally published in: Daniele, J. R., Heydari, K., Arriaga, E. A. & Dillin, A. Identification and Characterization of Mitochondrial Subtypes in Caenorhabditis elegans via Analysis of Individual Mitochondria by Flow Cytometry. *Anal*. *Chem*. **88**, 6309–6316 (2016)^11^.

We used JC-9, a chemical dye, to characterize the spatial distribution of mitochondrial membrane potential (Δψ) in live nematodes (Fig. 4A)^11–13^. Many cellular and physiological phenomena (e.g. aging, metabolism, and stress-resistance) depend heavily on the functionality of mitochondria and indeed, mitochondrial membrane potential (Δψ) perturbation in nematodes, has been associated with lifespan extension, fertility, apoptosis, and increased stress resistance^14–19^. Here, we investigated differences in longitudinal profiling of Δψ as a function of larval development and between long-lived mutant animals.

After labeling of mitochondria in live animals with Δψ-responsive JC-9 (Fig. 4A)^11^, which localized to mitochondria (Figure S4A), the red/yellow fluorescence ratio decreased when treating with the depolarizing agent valinomycin, which is consistent with the properties of this ratiometric dye (Figs 4B and S4B,C). These changes were also present in the comparison of the “Red” channel to the “Yellow” channel in micrograph images confirming the responsiveness of the dye (Figure S4D). The highly asymmetric spatial patterning of yellow fluorescent “JC-9 Monomer” enabled orientation (Fig. 4C) and subsequent longitudinal membrane potential measurements (Fig. 4D) of live-labeled animals. The profiles of Δψ under polarized and depolarized conditions (see median and MAD profiles in Fig. 4E and Figure S4E, respectively) showed similar regional trends with expected lower red/yellow fluorescence ratio for valinomycin treated animals. Interestingly, the most significant changes in Δψ upon depolarization were in the “Middle” and “Posterior” regions (Figure S4F), with more significant changes at the intestinal boundaries (~25% and ~75%, Fig. 4F). Thus, the longitudinal profiling of Δψ in live worms enables us to rapidly and effectively identify biologically relevant, region-specific changes, in Δψ.

### Longitudinal Changes in Mitochondrial Membrane Potential During Development

**Figure 5:**
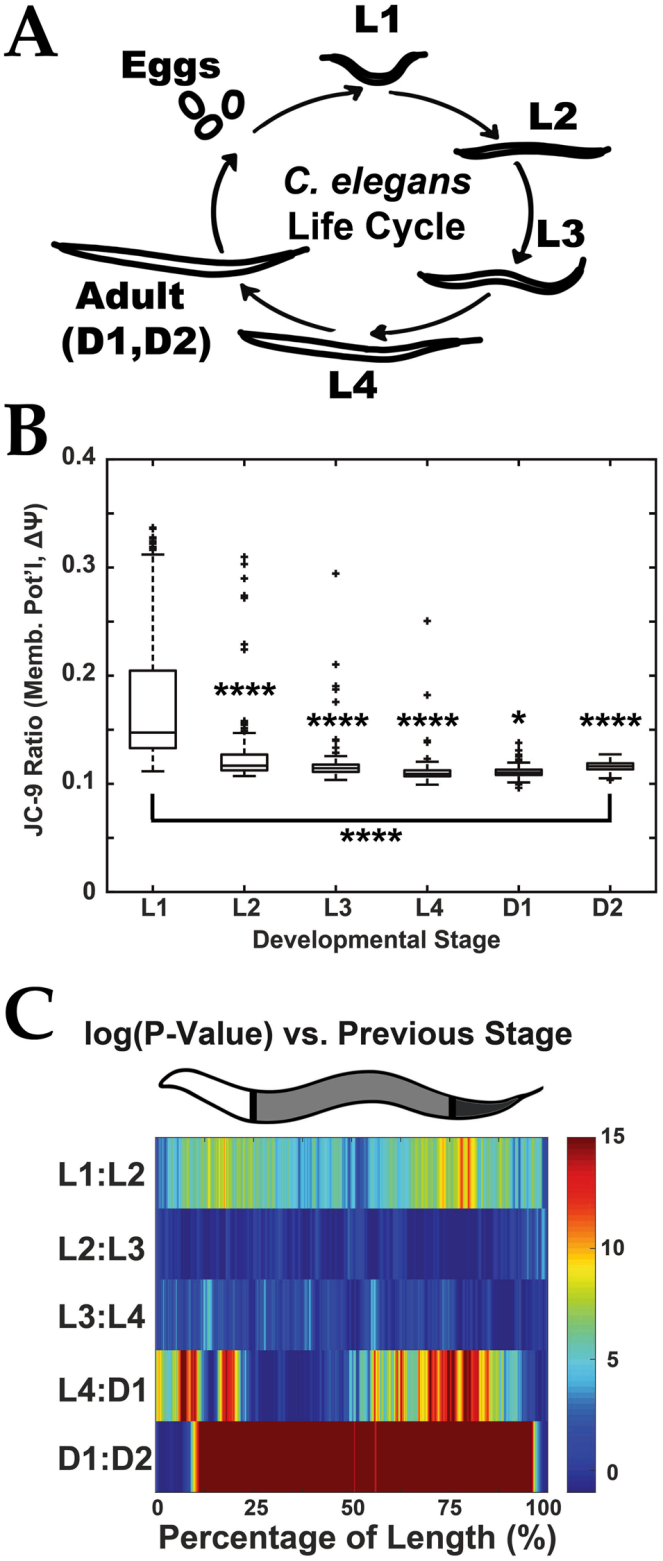
Mitochondrial membrane potential (Δψ) measurement during early *C*. *elegans* development. (**A**) Schematic of *C*. *elegans* life cycle to illustrate size and morphology differences. (**B**) Quantification of Δψ (by JC9–9 ratio) from live nematodes labeled with JC-9 at various stages of development. *P < 0.05, ****P < 0.00001. (**C**) Significance (log(*P-value*)) plots derived from median profiles in Figure S5H comparing each profile to its previous stage (e.g. L1–L2). For significance plots a value ≥ 0 is equivalent to *P* ≤ 0.05 by Wilcoxon Rank Sum Test. Plots are representative of three biological replicates and derived from *n* ≥ 50 organisms. Sample size (*n*) for all experiments and exact *P-values* can be found in the Supplementary Table. L.A. Daniele provided the illustrations for Part A originally published in: Daniele, J. R., Heydari, K., Arriaga, E. A. & Dillin, A. Identification and Characterization of Mitochondrial Subtypes in Caenorhabditis elegans via Analysis of Individual Mitochondria by Flow Cytometry. *Anal*. *Chem*. **88**, 6309–6316 (2016)^11^.

Several groups have reported that Δψ increases when tissues are developing and expanding in mammalian systems^20, 21^. Similarly, we measured Δψ in isolated mitochondria from *C*. *elegans* larval stages (Fig 5A) and noticed a ~50 fold higher Δψ in the L1-L2 larval stages relative to L4s and adults^11^. This work did not define longitudinal regions of the animal where Δψ was high in early development. Similar to these findings^11^, the overall Δψ (Red:Yellow fluorescent ratio) of whole worms decreases from L1 to L4 (Figs 5B, S5D-I, and Supplementary Information). This trend is also observed, though with less sensitivity, using microscopy (Figure S5A).

Since nematode development proceeds in several stages differing in the body sizes/optical densities (S5B, C)and tissue composition of animals^22, 23^, it would not be surprising if we observed that mitochondrial activity (e.g. metabolic demand) also varied along the worm profile. Indeed, profiles of Δψ at each larval stage are clearly distinct from each other (see difference between medians and statistical significance in Figs S5J and 5C, respectively). The most dramatic difference in Δψ is between L1-L2 larval stages, but the most statistically significant change is between Day 1 and Day 2 adults (see Figs S5J and 5C). The most significant differences were in L1 – L2, L4 – Day 1 adult, and Day 1–2 adult comparisons with the most dramatic Δψ occurring at the “Anterior” and “Posterior” regions (Figure S5K). Remarkably, one subtle difference that could have gone unnoticed without a regional analysis would have been that of the “Anterior” region between the L4 and Day 1 adult transition. Thus, the application of LAMPro to the functional measurement of Δψ in all size and stage *C*. *elegans* enables us to identify biologically relevant, region-specific, changes in mitochondrial health.

### Longitudinal Differences in Mitochondrial Membrane Potential in Long-Lived Mutants

**Figure 6:**
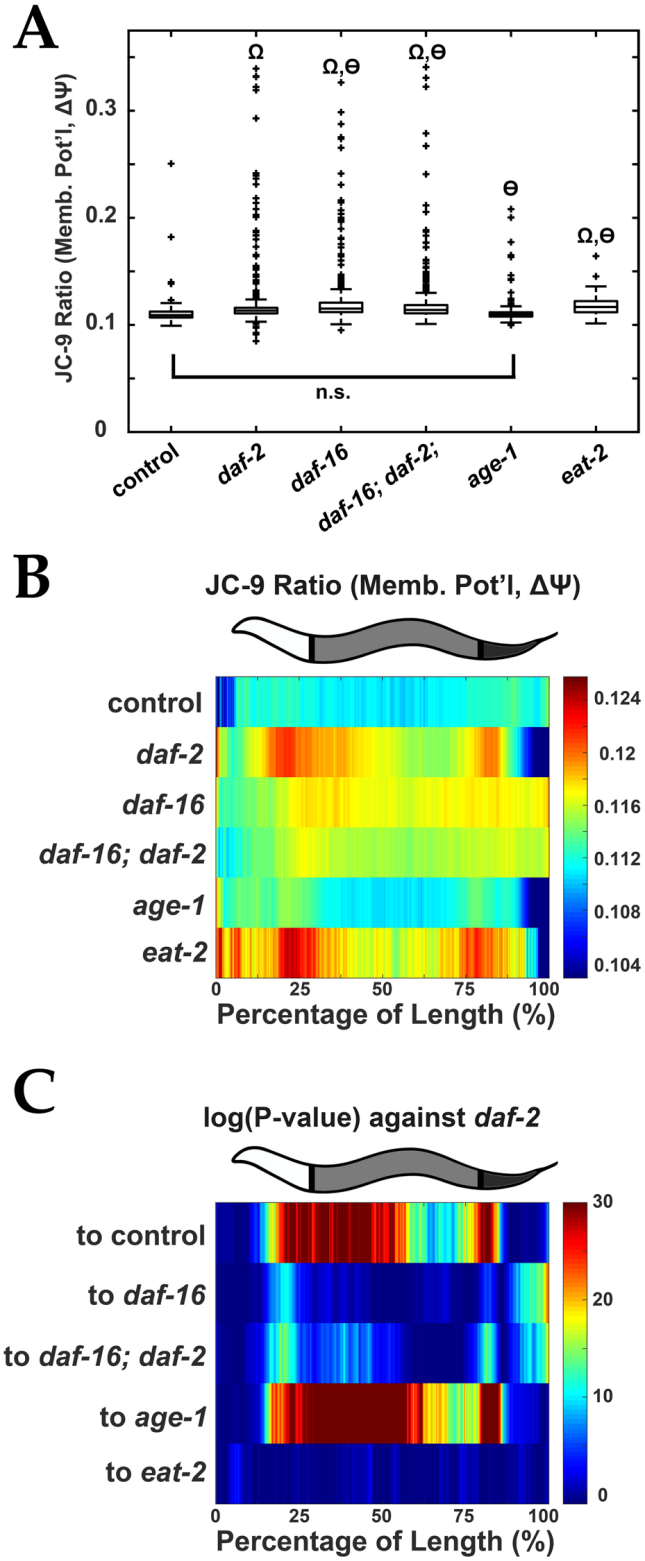
Mitochondrial membrane potential (Δψ) measurement of *C*. *elegans* longevity mutants. (**A**) Quantification of Δψ (by JC-9 ratio) for various long-lived nematode mutants. Wilcoxon Rank Sum tests were performed against N2 control (Ω = *P* < 0.00001) or against *daf-2* (ѳ = *P* < 0.00001) strains. (**B**) Median profiles displaying spatial distribution of JC-9 ratio of *C*. *elegans* longevity strains. (**C**) Significance (log_10_(*P-value*)) plots derived from median profiles in (**B**) comparing each profile to *daf-2* nematodes. For significance plots a value ≥ 0 is equivalent to *P* ≤ 0.05 by Wilcoxon Rank Sum Test. All animals are positioned with the anterior side to the left. Plots are representative of three biological replicates and derived from *n* ≥ 50 organisms. Sample size (*n*) for all experiments and exact *P-values* can be found in the Supplementary Table. L.A. Daniele provided the illustrations.

Nematodes with mutations in the insulin receptor, *daf-2*, not only live longer (2x lifespan increase^24^) but also have higher Δψ (~1.3x)^14^. Using *daf-2* mutants as a control for elevated Δψ, we sought to determine if LAMPro could distinguish a panel of longevity mutants based on their longitudinal pattern of Δψ. A comparison of overall Δψ in several longevity mutants (*daf-2*, *age-1*, and *eat-2*) and controls (N2:wild type background, *daf-16*: mutated downstream effector of *daf-2* (short lived), and *daf-16; daf-2*: mutated downstream effector in *daf-2* background (normal lifespan)) showed that median Δψ is significantly higher than N2 “control” for all strains except *age-1* (Figs 6A and S6A). In contrast, longitudinal profiling of Δψ shows that *daf-2* and *eat-2* mutants have unique regions at high Δψ (median and variability are in Fig. 6B and Figure S6B, respectively). Comparison between the Δψ profiles of these two strains shows no significant difference between *daf-2* and *eat- 2* (Fig. 6C). Mutants in *age-1*, however, possessed an entirely different longitudinal Δψ profile from *daf-*2. These observations suggest that positional Δψ provides a new dimension to the effects of mutation on different signaling pathways and, more generally, to the quality of phenotypic analysis in future studies of longevity.

### High-Throughput Longitudinal Profiling of Tissue-Specific Mitochondrial Morphology Mutants

Genetic screening in *C*. *elegans*, done through treatment with RNAi libraries, has identified genes that control mitochondrial morphology in adult muscle^25–29^. Much less is known, however, about the variability between tissues and the regulation of mitochondrial morphology within the cells of a given tissue. High-throughput longitudinal profiling of *C*. *elegans* treated with a subset of the RNA library previously reported to control mitochondrial morphology^28^ could unveil genes responsible for abnormal mitochondrial patterns in *C*. *elegans* muscle, an accepted model for mitochondrial dynamics research. Importantly, this gene set was identified in larval L4 or older animals. Which of these genes, when downregulated, leads to normal development but aberrant mitochondrial morphology? To test this technical hypothesis, nematodes possessing a mitochondrially-targeted GFP only expressed in the muscle (and a tdTomato-labeled pharynx to enable orientation) (see Figure S7A–E) were fed a panel of RNAi-expressing bacteria (in a 96-well plate format) that had previously been associated with maintenance of mitochondrial morphology (see Figure S7F–K)^28, 30^. Most treatments increased total mitochondrial signal (see Figure S7F), but largely preserved animal length and width/granularity (see Figure S7G,H). Longitudinal profiling of worms from each individual treatment (i.e. individual well) showed how each RNAi treatment affected local GFP expression (Figures S7I,J), making it possible to identify how muscle mitochondria morphology significantly varied relative to that of the “empty vector” control.

**Figure 7:**
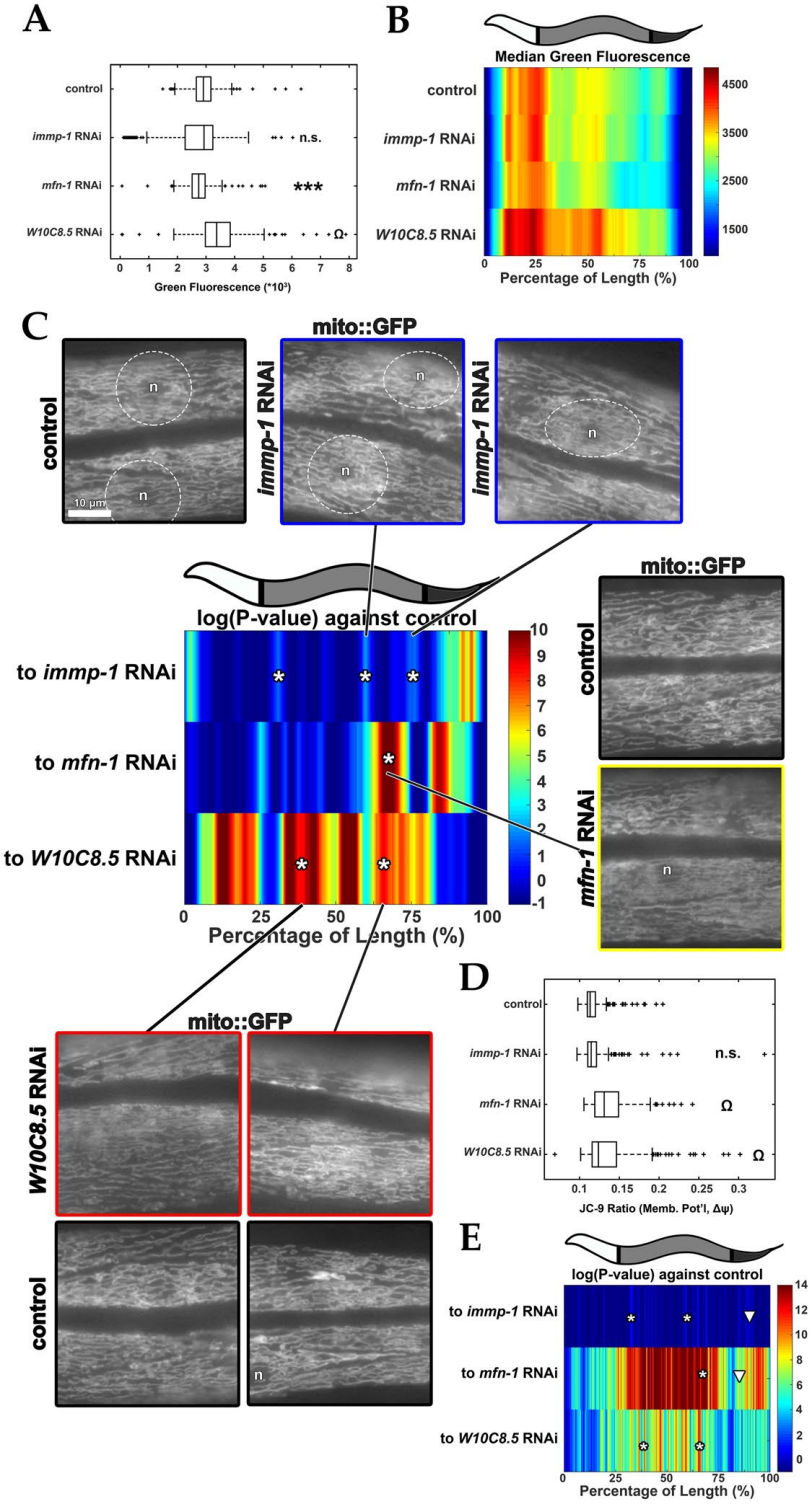
Mitochondrial morphology screening after treating *C*. *elegans* with selected RNAi’s. (**A**) Mitochondrial mass after selected RNAi knockdown of mitochondrial morphology genes. Muscle-specific, mitochondria-localized MLS2::GFP = “mito::GFP”. “n.s.” = not significant, ***P < 0.001, and “Ω” = *P* < 0.00001. (**B**) Median profiles of mitochondrial MLS2::GFP after RNAi treatments. (**C**) Significance (log_10_(*P-value*)) plots derived from median profiles in (**B**) compared to “empty vector” (control). Asterisks (*) = regions that deviate significantly from controls. For significance plots, a value ≥ 0 is equivalent to *P* ≤ 0.05 by Wilcoxon Rank Sum Test. Plots are representative of two biological replicates of *n* ≥ 100 organisms. High magnification micrographs of muscle-specific, matrix-localized MLS2::GFP signal, across a representative animal for control (empty vector, black boxes), *immp-1* RNAi (blue boxes), *mfn-1* RNAi (yellow box), and *W108*.*5* RNAi (red boxes). Scale bar is 10 µm. Muscle cell nuclei = “n”. (**D**) Mitochondrial membrane potential, Δψ (by JC-9 ratio) after RNAi treatments in (**A–C**). ***P < 0.001, and “Ω” = *P* ≤ 0.00001. (**E**) Significance (log_10_(*P-value*)) plots derived from median Δψ comparing each profile to “empty vector” treated “controls. Asterisks (*) = identified sites in (**C**) where Δψ is also affected. White arrowheads = positions where both mitochondrial morphology (in **C**) and Δψ are affected. Plots in (**D**,**E**) are representative of four biological replicates of *n* ≥ 100 organisms. Sample size (*n*) for all experiments and exact *P-values* for Wilcoxon Rank Sum tests against empty vector “control” can be found in the Supplementary Table. L.A. Daniele provided the illustrations.

By systematically employing our software’s ability to detect and visualize subtle, yet highly significant, regional differences in the fluorescent trace of a muscle-specific mitochondrial marker, we have established a protocol for the discovery of subtle variants in mitochondrial morphology. Notably, none of these regions would have been identified from the direct, more traditional, measurements of “whole” GFP fluorescence (mitochondrial mass) and TOF (animal size) in the biosorter (compare Figure S8A,B to Fig. 7A). In contrast, when we mined profile differences with microscopy, we could observe the mitochondrial variants that led to these regional differences (Figs 7B,C and S8C). The *C24H11*.*6* (*immp-1*) gene, an inner-membrane mitochondrial protease, would not have been identified based on the TOF and green fluorescence, which was not significantly different from the control (Figs 7A–C and S8A–C). In contrast, its median profile shows regional differences from that of the control strain (Fig. 7C, asterisks mark regions where GFP signal deviates significantly from controls). Fluorescence micrographs of the regions defined by the profile suggests an increase in lamellar mitochondria in the perinuclear region (Fig. 7C, white circles) of the muscle cells (blue boxes in Fig. 7C; compare Figure S8D and S8E and see Fig. S8H,I). Targeting the *W02B12*.*9* (*mfn-1*) gene, encoding a MitoFerrin implicated in iron transport to mitochondria, resulted in a lower green fluorescence (Figs 7A–C). The median profile illustrates where this RNAi treatment causes changes in GFP fluorescence relative to the control (Fig. 7B). Microscopy imaging associates thinner and more tubular mitochondria to the regions with the most significant change in GFP fluorescence (yellow box in Fig. 7C; comparison of Figures S8D and S8F). Lastly, targeting the *W10C8*.*5* gene, a creatine kinase, resulted in a slightly higher, green fluorescence than the control after application of the exclusion criteria (Figs 7A–C and S8A–C). Microscopy imaging of profile regions that are significantly different (red box in Fig. 7C; comparison of Figure S8D and S8G) showed thinner mitochondria, similar to that observed upon treatment with RNAi targeting of *mfn-1*. Finally, we measured mitochondrial membrane potential (Δψ) finding that *immp-1* RNAi treatment overall decreased Δψ (Figs 7D and S8K,M), while *mfn-1* and *W10C8*.*5* RNAi treatment, increased Δψ across the entire profile (Figs 7D and S8K,M). In contrast, the three RNAi treatments decreased mitochondrial mass overall (Figure S8J). Strikingly, when membrane potential was compared along each profile, as in 7C, several regions that showed large changes in mitochondrial morphology also appeared to differ in Δψ (Fig. 7E, compare asterisks to corresponding ones in Fig. 7C). Collectively, the ability to perform live screens based on comparisons of longitudinal profiles should (1) facilitate the identification of mutants with significant changes in regional mitochondrial mass and (2) enable one to morphologically and functionally characterize the areas/tissues most affected in complex mitochondrial phenotypes in living animals.

## Discussion

Biosorters have been instrumental to the development of high-throughput analyses of *C*. *elegans*. The resulting data however, consists of a limited number of measurements (e.g. total fluorescence in three channels (green, yellow, and red), object length (time of flight, TOF) and granularity/width (extinction, EXT)) per animal^2, 4–6^. Individual measurements taken along the length of each animal have not been utilized in such studies, ultimately overlooking positional information inherent to the data. To address this issue, several biosorter data analysis programs have been reported^2, 4–6^, but they have limitations in regard to (1) exclusion of artefactual data, (2) alignment of profiles, and (3) data visualization. The software (LAMPro) described here addresses these issues and enables region-specific phenotypic characterization and longitudinal comparisons in large cohorts of *C*. *elegans* data. In addition, this software has broader applications because it can effectively align and visualize biosorter data from a wide range of organisms including *Drosophila* embryos, larval mosquito, and larval zebrafish (data not shown). LAMPro excludes aberrant profiles, thereby reducing biologically irrelevant variability. LAMPro also systematically aligns and compares longitudinal profiles of different organism models displaying a wide range of sizes, widths, and granularities (see Fig. 1B,C), of *C*. *elegans* at different developmental stages despite their differences in length (see Figs 4C and 5) and of *C*. *elegans* of genetically manipulated strains displaying different phenotypes (see Figs 2A, 3A, 6B and 7B). Finally, LAMPro enhances the quality of comparisons between longitudinal profiles through (heat-map) visualization of median, difference, statistical significance, and MAD profiles and distills positional information for determination of regional significance. Through its current capabilities (e.g. visualization of median profiles, detection of regional differences and quantification of their significance), LAMPro can be used for systematic high-throughput screening in a 96-well plate format, enabling selection of region specific phenotypes when using a library of treatments.

In our applications of LAMPro three factors were carefully considered to collect data of high quality. First, we applied an exclusion criteria to eliminate viable worms that had clumps of bacteria (from the food plate) sticking to their head or tail, which caused high Extinction peaks on the profile edges and a deformation/distortion of the fluorescent trace (Figure S1C,D). Notably, this criterion also eliminates profiles of folded worms traveling through the detector. To reduce unnecessary exclusion of viable profiles, we suggest one spend more time washing worms before running them on the biosorter. Second, the fidelity of the orientation algorithm requires asymmetric profiles. A symmetric trace, for instance, would be difficult to orient since “forward” and “backward” facing animals would look similar. Similarly, for more complex profiles, e.g. those that possess two or more peaks, other algorithms may be needed. When in doubt, a comparison of the aligned profiles with line plots from micrographs (e.g. similar to how we developed the “Derivative Test”, Figure S5D) or use of asymmetric fluorescent markers (e.g. *myo-2p::*tdTomato) is recommended for determining the proper orientation algorithm to use. Third, the native fluorescence of animals (e.g. older worms) or fluorescence bleed-through of fluorescent proteins with similar emission spectra (e.g. GFP and YFP) can confound the outputs of the algorithms. Proper configuration of the biosorter optics (e.g. selection of filter cubes and PMT settings) to match the spectral characteristics of the fluorescent proteins will reduce complications with bleed-through. The correct setting of threshold conditions (e.g. basal fluorescent conditions, “default” settings can be found in Supplemental Methods) on the sorter (e.g. “Store Gated” condition) or as “basal fluorescence” values in the Perl code (lines 897–899) can also reduce these potential biases.

It is currently unknown why differential sensitivity to UPR^mt^ exists in various *C*. *elegans* tissues. Transcriptional reporters, such as *hsp-6p* *::gfp*, are commonly used to investigate stress in *C*. *elegans* ^1, 3^. Longitudinal profiling of *hsp-6p* *::gfp* expression illustrated that mitochondrial stress can have disparate and unique patterns of UPR^mt^ activation dependent on the genetic model (Fig. 3A). Relative to the *cco-1* RNAi model, only treatment with *tim-17*(*2*) RNAi had mostly identical UPR^mt^ activation profiles, while the *mrps-5*, *spg-7*, and *tim-17*(*1*) RNAi were dramatically different (Fig. 3B). Further examination revealed regional significant differences between regions (“Anterior”, “Middle”, and “Posterior”), which should enable region-specific comparisons and screening (Fig. 3C). It is interesting that the *hsp-6p* *::gfp* activation between *cco-1* and *mrps-5* RNAi is so dramatically different when both treatments are known to extend lifespan via activation of UPR^mt^ ^1, 7^. One possible factor in this distinction is that the effects of *mrps-5* RNAi are mediated, presumably by “whole-body” knockdown of this protein while the lifespan extension via *cco-1* RNAi is likely a combination of (1) cell non-autonomous activation of UPR^mt^ in neurons (which then signal this mitochondrial stress to the intestine) and (2) the activation of UPR^mt^ cell autonomously in the intestine by the RNAi alone. A potential experiment which might enable one to parse out the region-specific contributions of these two forms of cell-cell communication is to compare the *hsp-6p::gfp* activation pattern between worms expressing a *cco-1* hairpin in exclusively neurons or exclusively intestine (in a *sid-1* background, so RNAi cannot be transmitted between tissues). Finally, we cannot discount the possibility that some of the observed differences could be due to variability in the efficiency of RNAi knockdown, thus more precise experiments using serial dilutions of each RNAi expressing bacteria, in conjunction with the sorter, should enable the identification of tissues “most sensitive” to reporter activation. This future work, however, does not undermine the impact that this methodology would have in future studies requiring comparisons of region-specific transcriptional reporters and its potential use in discovery of additional regulators that maintain or vary the positional expression of biologically relevant reporters.

Through comparisons of longitudinal profiles of Δψ (Figs 4 and 5) during nematode development, we identified dramatic changes that would be lost if taking only one Δψ measurement per animal. The “Anterior” and “Posterior” regions in early development (L1–L2) had the largest differences of Δψ, but all three regions showed statistically significant differences between adjacent larval stages (Fig. 5). Dramatic and transient increases in mitochondrial Δψ have been associated with the differentiation/expansion of “new tissues” (i.e. nervous system, liver development^20, 21^) and we have observed a comparable transient spike in Δψ in mitochondria isolated from egg to larval L2 stages^11^. Dramatically, the most significant differences in Δψ were between Day 1 and Day 2 adults, for each regional comparison, and the “Anterior” and “Posterior” regions of the transition from larval (L4) to adulthood (Day 1 adult) (see Fig. 5C). Significance in Δψ difference in Days 1 and 2 is unlikely to be attributed to eggs because our JC-9-labeling protocol results in a limited staining of germline and eggs relative to somatic tissues (Figure S2G); nevertheless, changes in Δψ from L4 larval to Day 2 adult stages could still be associated with germline development^22, 23^. Future comparisons between larval stages may include screening for regulators of Δψ during development.

Long-lived nematodes with mutations in the insulin receptor, *daf-2*, have higher Δψ than wild-type strains^14^, which posits mitochondria as a factor for longevity. It is not known whether other mutants directly or peripherally associated with the insulin/IGF-like signaling pathway (IIS) display elevated Δψ and whether the elevated Δψ is positional along the length of the nematode. Comparison of longitudinal Δψ profiles of various longevity mutants (*daf-2*, *age-1*, and *eat-2*) provided great insight (Fig. 6). The *daf-2* mutant displayed elevated Δψ in two regions (~25% and ~80% from the head end) (see Fig. 6B). The elevated Δψ regions seen in *daf-2* mutants are not prevalent when *daf-16* (or *daf-16; daf-2*) are mutated (Fig. 6B). When the insulin receptor (DAF-2) binds insulin, the transcription factor DAF-16 becomes phosphorylated and is excluded from the nucleus. In the absence of DAF-2, DAF-16 is constitutively sent to the nucleus, activating genes implicated in starvation response, stress resistance, and lifespan extension^31^. Notably, the *age-1* mutant did not show regions of elevated Δψ. Because AGE-1, a phosphatidylinositol-3-kinase, is upstream of DAF-16 phosphorylation^31–33^, the lack of an elevated Δψ, suggests DAF-16 nuclear translocation is associated with region-specific elevation of Δψ. Finally, the regional dependence of Δψ is remarkably similar between the *eat-2* and the *daf-2* mutants. EAT-2 is a pharyngeal ion channel, which causes feeding defects (dietary restriction) when mutated^31–33^, but does not induce the nuclear localization of DAF-16 ^31^, suggesting the Δψ is not uniquely associated with the nuclear localization of DAF-16. Undoubtedly, longitudinal profiling of Δψ could be extended to other mutants to explore the relevance of Δψ in other models of longevity and disease (e.g. neurodegeneration)^34, 35^.

While conventional genetic screens reveal the identity of proteins that are important in global maintenance of mitochondrial morphology^25–29^, information regarding the anatomical regions where such control takes place often requires extensive follow-up experiments. We developed LAMPro to discover new mutants that would not have been previously identified based on the GFP fluorescence of each animal measured by the biosorter, but could be defined by longitudinal profiling of a muscle-specific mitochondria-targeted GFP under various RNAi treatments, which reportedly alter muscle mitochondria morphology^28^. The treatments with RNAi against *C24H11*.*6* (*immp-1*), *W02B12*.*9* (*mfn-1*) and *W10C8*.*5* genes were chosen to place emphasis on gene knockdowns that would not have been identified based exclusively on total GFP fluorescence prior to exclusion (compare Figure S8A to Fig. 7A) and possessed significant, isolated, changes in the longitudinal profiles (Fig. 7B). Because GFP fluorescence is a surrogate of muscle mitochondrial mass, it was anticipated that local differences in the longitudinal profile of GFP fluorescence might correspond to changes in mitochondrial morphology in the respective anatomical regions (Figs 7C and S8D–G). In agreement, the three RNAi treatments highlighted here, showed dramatic alterations in mitochondrial morphology, despite minimal changes in muscle mitochondrial mass. The RNAi targeting of the mitochondrial membrane protease gene *C24H11*.*6* (*immp-1*) led to phasic regions of high signal in lamellar mitochondria in the perinuclear regions of muscle cells in the mid region (Figs 7C, S8E, and S8H,I). Marked peaks of higher Δψ were also observed in this phasic pattern (amid overall lower Δψ), suggesting a functional preservation of local Δψ by this morphological change (Figs 7D,E and S8M). This webbed, bulbous, mitochondrial phenotype could be due to the abnormally long cristae membranes which bow the mitochondria^36^ and is likely to disrupt Δψ due to a decrease in mitochondrial transport of cytochrome subunits^37, 38^. The RNAi targeting of the ion transporter Mitoferrin gene, *W02B12*.*9* (*mfn-1*), by contrast, caused a dramatic thinning of mitochondria and a massive increase in tubulation close to the anterior region of the animal (Figs 7C and S8F), while a similar phenotype appeared in two different regions of the animal treated with *W10C8*.*5* RNAi (Figs 7C and S8G). Both mutants caused marked increases in Δψ and we observed higher Δψ in the same areas where mitochondrial morphology also differed (Figures S7D–E and S8M). Notably, *mfn-1* mutants have an extended lifespan and show UPR^mt^ activation^39, 40^, possibly due to a decrease in iron transport and increase in mitophagy^41^, which might explain the simultaneous decrease in mitochondrial mass and localized areas of increased Δψ. Thus, future uses of longitudinal profiling could make it possible to define high-throughput association studies (with *mfn-1* as a control) to link UPR^mt^ activation with mitochondrial morphology and Δψ, and then characterize hits for lifespan extension. Finally, although this wouldn’t require high-throughput techniques, one notable example where these tools could be immediately applied is in distinguishing the striking phenotypic differences (regional- Δψ, ATP levels, and ROS generation) and differential lifespan extensions of *nuo-6*; *isp-1* double mutants (for mitochondrial complex I and III subunits, respectively) and the single mutants paired with its RNAi counterpart (e.g. *nuo-6; isp-1* RNAi)^42, 43^.

## Concluding Remarks

Our development and application of the freely available bioanalytical software LAMPro to the study of mitochondrial biology has enabled the identification and characterization of region-specific mitochondrial function and morphologies. In particular, our discovery of concomitant regional changes in mitochondrial morphology and activity (Δψ) should pave the way for future *C*. *elegans* studies of the tissue specific etiology of complex physiological phenomena (e.g. mitochondrial bioenergetics) and disease. The ability to extend these studies to phenotypic RNAi and/or drug screens to reverse the effects of aging and neurodegenerative disease models on mitochondrial health are now indeed exciting possibilities.

## Materials and Methods

### Strains and Reagents Used

#### Strains

The following worm strains were obtained from the Caenorhabditis Genetics Center (Minneapolis, MN, USA): N2, CB1370 *daf-2*(*e1370*), CF1038 *daf-16* (*mu86*), CF1043 *daf-2*(*e1370*)*; daf-16*(*mu86*), DA1116 *eat-2* (*ad1116*), TJ1052 *age-1*(*hx546*), SJ4100 *zcIs13* (*hsp-6p* *::gfp*), RT130 *pwIs23* (germline/eggs expression, *vit-2::gfp*), AM101 *rmIs110* (nervous system expression, *rgef-1p* *::q40::yfp*), VS30 *hjSi158* (intestinal expression, *vha-6p* *::SEL-1(1–79)::* *mCherry::HDEL*::*let-858 3′UTR*), and IG274 *frIs7* (red hypodermis expression, *nlp-29p* *::gfp*; *col-12p* *::dsRed*). Additional transgenic worms with stable extrachromosomal arrays were created using the following vectors pGH8 (nervous system, *rab-3p* *::mCherry::unc-54 3'UTR*), pCFJ90 (pharynx, *myo-2p* *::mCherry::unc-54 3'UTR*), pCFJ104 (muscle, *myo-3p* *::mCherry::unc-54 3'UTR*). Co-injection of any one of these plasmids with the Mos1 transposase pCFJ601 (*eft-3p* *::Mos1 transposase*) dramatically improved the likelihood of creating stable extrachromosomal arrays. These mCherry plasmids all possessed an *unc-119* rescue protein to identify successful DNA injections. The germline of late larval L4 worms was injected as described previously into strain *eg6703* (*unc-119*(*ed3*) *III; cxTi10816 IV;*) which was mutant for *unc-119* ^44^. After injection worms that moved normally across the plate (or *unc-119*+) were picked and placed on new plates to enrich for worms with extrachromosomal arrays. One extrachromosomal line was isolated for each tissue described. The muscle-specific mitochondrially-targeted GFP strain, AGD1664, was created by fusing (1) a myo-3p muscle promoter, (2) a putative MLS from the mitochondrial gene *H28016*.*1a*, (3) a GFP cDNA, and (4) an *unc-54* UTR together (or “*myo-3p* *::MLS2::gfp::* *unc-54 3′UTR*”) and inserting this gene into the chromosome 1 locus *ttTi4348* by MosSCI integration. This strain was then crossed to strain AGD1582, containing *fer-15* (*b26*), *fem-1*(*hc17*), and *uthIs272*[*myo-2p* *::tdTomato*]. The resulting strain, AGD1756, contained *myo-3p*::MLS*::gfp*, *myo-2p* *::tdTomato*, and showed partial sterility at 25 °C.

#### Reagents

The ratiometric mitochondrial membrane potential sensor dye JC-9 (3,3′-Dimethyl-α-naphthoxacarbocyanine iodide) (D-22421) was purchased from Life Technologies (now Thermo Fisher) while valinomycin (V0627) and Levamisole Hydrochloride (31742) were from Sigma Aldrich.

### Live Labeling and Microscopy

#### Sensory Neuron Labeling

A subset of nematode sensory neurons was labeled in with DiO (D275, ThermoFisher) or DiI (D22885, ThermoFisher) to facilitate orientation in Figs 2 and 3. The excitation/emission wavelengths for DiO and DiI are 484/501 nm and 549/565 nm, respectively so strains expressing GFP were labeled with DiI and strains expressing mCherry or dsRed were labeled with DiO using a protocol modified from Schultz & Gumienny 2012^45^. Briefly, staged worms were rinsed off plates with M9 [42 mM Na_2_HPO_4_, 22 mM KH_2_PO_4_, 86 mM NaCl, and 1 mM MgSO_4_.7H_2_O] and added to Eppendorf tubes. Animals were spun down and supernatant was removed and replaced with 1 mL of M9 and 5 µl of DiO or DiI [2 mg/ml in DMF]. Tubes were rocked at 20 °C for 1 to 1½ hours and then spun down, washed with fresh M9 twice before animals were placed on fresh OP50 plates for at least 2 hours at 20 °C to allow the dye to pass through the digestive tract (eliminates non-specific labeling). Worms were then washed off the plates with fresh M9 and run through the biosorter.

#### Functional Mitochondrial Labeling

To label live worms for Figs 4–6 (with JC-9) 2 µL of liquid OP50 culture (normally used to seed NGM plates for culturing worms) was used to inoculate 1 mL of LB broth. This culture was then shaken at 37 °C for 4 hours. JC-9 (10 µL per 1 mL) of a 5 mM stock was then added to this culture and the tube was put at 37 °C for another 4–6 hours. Cultures were then spun down at 4400 × G for 10 minutes (to remove any JC-9 that was not taken up by the bacteria). These pellets were then resuspended with 500 uL of fresh, unlabeled, LB broth. This labeled culture was then seeded on NGM plates and allowed to dry overnight in a lightproof box (of note, all steps of this labeling used light-protected tubes to decrease the chances that the dye got bleached by light). Dry, seeded, plates were then used the next day when L4 staged worms were spotted on the plates for 1–2 hours at 20 °C. After labeling, worms were washed off plates, and run in the biosorter. When preparing samples for microscopy (for Fig. 7), individual worms were picked off plates, put on a microscope slide and anesthetized in 100 nM sodium azide solution. A coverslip was added and sealed with nail polish and live slides were taken to the microscope for immediate imaging. Specimens were viewed on a Leica DM60013 upright light microscope using the 10x or the 63x HC PL Fluotar Objectives, with constant acquisition settings when comparing specimens within a given experiment.

### Worm Staging and running on the Biosorter

Worm staging was performed at 20 °C in line with previously published developmental timing and worm growth media^46, 47^. Worms of the specified stages were washed off plates and resuspended in autoclaved M9 into 15 ml conical tubes. We used a Union Biometrica complex object parameter analysis sorter (COPAS) Biosorter (product no. 350–5000–000) using both 561 nm and a 488 nm light sources. After the Biosorter was calibrated and flushed with sequential 10% bleach, M9, COPAS cleaning solution (#300–5072–000), and finally, M9, worms were added to the “cup” or “hopper” and data was acquired using previously optimized settings for laser PMT power and size gating. More specifically, only data from worms of the proper size (using Extinction (EXT, “width”*) and Time of Flight (TOF, “length”) was stored (using the “Store Gated” option). This prevented the acquisition of data that resulted from debris, eggs, and unstaged (e.g. younger L1 or L2) worms from being saved when this was not the intention. *N.B. Although there is an empirically established a relationship between the EXT and object “width”, much like “Side Scatter” (or “90° scatter”) in conventional flow cytometry, these measurements also take into account additional properties including density, texture, and granularity.

### RNAi Feeding

Worms were fed from hatch HT115 *E*. *coli* containing an empty vector control or expressing double-stranded RNA. RNAi strains were taken from the Vidal library, if present, or from the Ahringer library if absent from the Vidal library. RNAi clones used were for the genes *cco-1* (a homologue of human COX5B, F26E4.9, Vidal #11012 A12), *spg-7* (a homologue of the human SPG7, Y47G6A_247.f, Ahringer #27 H12), *mrps-5* (a homologue of the mitochondrial 28S ribosomal S5, E02A10.1, Vidal #10012 A01), and two different RNAi constructs against *tim-17* (a homologue of the human mitochondrial importer, TIM-17, E04A4.5, Vidal #10001 D7 (“*tim-17*(*2*)”) and Ahringer #97 G12 (“*tim-17*(*1*)”) and an empty vector “control” which consisted only of the L4440 vector. All RNAi clones were sequence verified prior to use and knockdown verified previously^3, 7, 48, 49^. For mitochondrial dynamics experiments, L1 arrested worms were grown to L4 on RNAi strains which came from a documented and published RNAi library of genes implicated in the maintenance of mitochondrial morphology^28, 30^.

### Software Specifications and Data Analysis

Worm profile data was collected using the Biosort 5401.1 software provided for use with the biosorter machine. Prior to running our orientation algorithm (described below), all profiles were converted from one of multiple.dat file(s) into a single.txt file using the “Export as Text” function in the Profile Reader 16.1 software (also sold by Union Biometrica) and then saved in a folder alongside the.lmd and.dat files that were saved by the Biosorter at the time of data acquisition. All orientation algorithms were written in Perl5 and interpreted in Cygwin (a Linux API that can run on Windows). Data analysis, significance testing, and data plotting was run using MATLAB scripts (version R2015a) which have been integrated into a single graphical user interface (“LAMPro Suite”). A flow chart for this “Generalized Workflow for Data Processing” is available in the Supplementary Information.

Regarding data processing and analysis, we discovered several conditions inherent to data acquisition in a biosorter that led to non-ideal data. We developed experiments and exclusion criteria to eliminate the effects of (1) small, non-staged animals and debris e.g. clumps of bacteria, eggs, L1 and L2 worms, (2) animals with aberrant extinction profiles (e.g. ones that had peaks on the edges and differed substantially from the “ideal” profile in Fig. 1A (also see Figure S1)), and (3) animals that were not labeled sufficiently with the intended dye or expressed a fluorescent transgene above “background”. To ensure experiments had adequate statistical power, we only analyzed data from runs with *n* ≥ 50 animals per run (and most experiments had *n* ≥ 150 animals). Notably, these sample sizes were reflective of the data after our software’s exclusion criteria had been applied.

More detailed information regarding the development of the alignment software, worm exclusion criteria, MATLAB scripts for data visualization (e.g. Heatmap, median profile, Tukey box plots (median with 1.5 interquartile range above and below), regional significance) can be found in the Supplementary Information. Of note, our software can detect and visualize when a fluorescent reporter becomes saturated (e.g. large portions of the profiles are at max signal intensity) which is likely to eliminate fine differences in activation patterns (see Figure S3D–F). A flow chart for this “Workflow for LAMPro Algorithm” is available in the Supplementary Information.

For a detailed, step-by-step, walkthrough of the workflow necessary for formatting the files and then running the software refer to the section titled “LAMPro Directions” in the Supplementary Information. Links to download all these programs freely is available through the journal’s Supplemental Material.

## Electronic supplementary material

Supplementary Information

Software
