# Supplementary material for: “High-Throughput Characterization of Region-Specific Mitochondrial Function and Morphology”
Source: Sci Rep. 2017 Jul 27;7:6749. doi: 10.1038/s41598-017-05152-z (PMC5532364; doi:10.1038/s41598-017-05152-z)
Supplement: Supplementary file 1 — Supplementary Information [file 41598_2017_5152_MOESM1_ESM.pdf]

SUPPLEMENTARY INFORMATION

"High-Throughput Characterization of Region-Specific Mitochondrial Function and Morphology."

Joseph R. Daniele[1], Daniel J. Esping[2], Gilbert Garcia[1], Lee S. Parsons[2], Edgar A. Arriaga*[2] & Andrew Dillin[1]

1. Department of Molecular & Cellular Biology,

University of California, Berkeley

Berkeley, CA 94720-3370

2. Department of Chemistry,

University of Minnesota

Minneapolis, MN 55455

*** = Corresponding Author: arriaga@umn.edu**

Keywords: large particle flow cytometry, longitudinal profiling, mitochondrial membrane potential, live labeling, *C. elegans*

SI Table of Contents

SUPPLEMENTARY METHODS 3
WORKFLOWS FOR DATA PROCESSING AND LAMPRO ALGORITHM 8
SUPPLEMENTAL REFERENCES 9
SUPPLEMENTARY FIGURE LEGENDS 10
SUPPLEMENTARY FIGURES 14
LAMPro DIRECTIONS 22
LAMPro GUI DIRECTIONS 33
SETUP 33
LAMPro GUI 34
1. Select File 35
2. Choose Figure Directory 36
3. Select Desired Figures 38
A. Standard Figure Type 38
B. Comparison Figure Type 39
SD. Summary Figure Type 40
E. Regional Comparison Figure Type 40
LAMPro WELLS GUI 42
1. Select File 42
2. Choose Figure Directory 42
3. Select Desired Figures 43
A. Standard Figure Type 43
B. Comparison Figure Type 43
C. Summary Figure Type 44

SUPPLEMENTARY METHODS

Strains and Reagents Used

Strains: Worms with blue fluorescent protein (“TagBFP” or “BFP”) expressed exclusively in one tissue were created using pieces from five vectors: (1) a promoter (see below), (2) the mitochondrial localization signal from the Fire lab *C. elegans* vector kit (pD96.32), (3) a codon optimized TagBFP generously given to us by the Abby Dernberg lab (pSK16)[1], and (4) the 3’ UTR from the *unc-54* transcript also from the Fire lab *C. elegans* vector kit (pD96.32), and (5) a vector backbone (pCFJ365) which included an *unc-119* rescue protein to identify successful DNA injections. The promoters used were (1) the muscle specific promoter from the *myo-3* gene generously given to us by the Barbara Meyer Lab (pCFJ104), (2) the pan-neuronal specific promoter cloned from the *rgef-1* gene, (3) the hypodermis specific promoter cloned from the *lin-26* gene, and (4) the intestine specific promoter cloned from the *vha-6* gene. The germline of late larval L4 worms was injected as described previously into strain *eg6703* (*unc-119(ed3) III; cxTi10816 IV;)* which was mutant for *unc-119*[2]. After injection, worms that moved normally across the plate (or *unc-119* +) were picked and placed on new plates to enrich for worms with extrachromosomal arrays. One extrachromosomal line was isolated for each tissue-specific transgenic (strains AGD1745, AGD1804, AGD1810, AGD1812) and used in this paper.

Live Labeling and Microscopy

To label live worms (which were used to validate MLS::BFP labeling in the various tissues) OP50 was labeled with JC-9 as previously described, spotted on NGM plates and kept in a lightproof box[3]. Dry, seeded, plates were then used the next day when L4 staged worms were spotted on the plates for 1-2 hours at 20º C. After worm labeling, individual worms were taken off plates, put on a microscope slide and anesthetized in levamisole hydrochloride (10 mM) solution. A coverslip was added and sealed with nail polish and live slides were taken to the confocal for immediate imaging. Specimens were viewed on a Zeiss LSM700 Inverted confocal microscope, with constant acquisition settings when comparing specimens within a given experiment. Worms with both the muscle-specific MLS::BFP and MLS2::GFP were also imaged in this manner. Images in **Figure S4A, S7A** are maximum intensity projections (using the ‘Processing’ tab in Zen software) of stacks spanning the width of various tissues within an anesthetized, unlabeled, transgenic animal. Image quantification in **Figure S8G-H** was performed by identifying muscle nuclei (characterized by a lack of mitochondrial signal) and then drawing a circular region of interest (ROI) with a diameter equal to the width of each strip of muscle. The “Radial Profile” plug-in in ImageJ was then employed on the ROI to measure the mean fluorescent values on the perimeter of increasing concentric circles emanating out from the center. The “normalized” fluorescence of the perinuclear region was calculated by taking the integrated fluorescence from 1/8 (12.5%) up to ½ the radius (50%) from the nucleus. Images in **Figures S1D, S2A, S2F, S3B, S4D, S5A, S6A, 7C, and S8D-G** were taken on a Leica DM60013 upright light microscope using the 10x or the 63x HC PL Fluotar Objectives, with constant acquisition settings when comparing specimens within a given experiment.

GFP Bacteria labeling: A GFP-expressing plasmid (a kind gift from the Don Rio lab) was transformed into competent HT115 *E. coli* bacteria and a single GFP-positive colony was grown overnight and seeded on NGM plates. Nematodes were fed from hatch on these GFP-bacteria plates, grown to L4 larval stage, washed off plates and run in the biosorter. Orientations were performed using a fluorescent pharynx or DiI as a marker.

LAMPro Software Details and Specifications

The Profile Reader software will sometimes fail if the number of profiles is too large (e.g. data from a 96 well plate). In these cases, we recommend saving each well as an individual file (use the "Export individual wells as single files" toggle) which adds a "PA_W#" to the exported file name, where # is the well number. Data analysis, significance testing, and data plotting was run using Matlab scripts. It should be noted that a version of **L**ongitudinal **A**lignment **M**etabolic **Pro**filer ('LAMPro') and the Matlab scripts to visualize the data that are compatible with data derived from a 96-well plate has also been provided in the supplement. This should enable our software to be applied to high-throughput screening.

Algorithms Developed

Length Normalization Algorithm: To facilitate orientation and allow for worms of very different sizes to be compared we developed a "length normalization" algorithm which fixed all profiles at 400 "data points". While this was roughly the "length" in TOF of most L4 worms, the range we observed in TOF was from ~100 points for the smallest L1 larval animals to ~600+ for animals that were in Day 2 of adulthood. To enable the transformation of this range of objects, an interpolation method of averaging was employed. This method created increments of 0.25% from '0' to '100' percent for each profile. This system was able to create high resolution profiles which replicated the fluorescent signatures of the worms faithfully regardless of whether the profile was "scaled" up or down (**Figure S1A-B**, respectively).

Orientation by K means Testing: To enable positional statistics to be performed, we developed an orientation algorithm. We assumed that worms move through the sorter in opposite orientations, head-first and tail-first, which we considered distinct data clusters. Since profiles length was normalized (c.f. *Length Normalization Algorithm*), a modified Pearson Chi Squared (X^2) test was performed to compare the intensities in the desired channel at each percentile point between two profiles ("profile x" and "profile y") using Equation (1).

$$\chi^2 = \frac{1}{N} \sum_{x,y=1}^{N} \frac{(O_x - x)(O_y - y)}{\sigma_x \sigma_y}$$

Equation (1):

Where the O_x or O_y were the observed extinction intensities, the *x* or *y* were the mean extinction intensities, σ_x or σ_y were the standard deviation of the extinction intensities. The sample size, N, is the standardized length of the profile, which was set to 400 for all *C. elegans*.

Initial testing using the Pearson X^2 test to align profiles proved adequate for highly asymmetric profiles (e.g. pharynx marker, 76.1 ± 7.1%, Mean ± SEM) but when profiles had multiple peaks, despite being highly asymmetric, the Pearson test was much less reliable (e.g. neuronal marker, 61.7 ± 13.2%, Mean ± SEM). N.B. Orientation fidelity was validated by running the software on 50 profiles from various data sets in which we knew the what a correctly orientated profile (using an asymmetrical marker in another channel or using a micrograph line scan for reference) looked like and then counting by hand the number or incorrectly aligned profiles. It should be noted that orienting using physical properties (density, texture, granularity or 'Extinction') proved inappropriate since high-quality EXT *C. elegans* profiles are symmetrical (see section on ***Exclusion Criteria***) For other organisms, however, like zebrafish, orientation by EXT works very well.

Thus, to improve our orientation algorithm, our Pearson X^2 test was coupled with a "k-means" clustering algorithm test[4,5]. Briefly, the k-means test is a sorting algorithm that categorizes profiles into k number of groups. Since only forward and reverse facing groups are possible, a k=2 was used. Each profile is placed randomly into one of the two groups. The program then calculates a mean X^2 value for both groups from the X^2 values of each worm. Worms are then switched, iteratively, between the two groups until both groups have reached the maximal mean X^2. This sorting is repeated until there was no growth in either group mean-X^2 values. The "reverse"-facing ('tail-first') group is then "flipped" and combined with the "forward"-facing group ('head-first'). This orientation algorithm dramatically improved the accuracy of acutely asymmetric profiles (e.g. see **Figure 1B**, and **Figure S2B & S2C**, and 97.4 ± 1.5% and 95.4 ± 2.2%, Mean ± SEM, oriented correctly for a pharynx and neuronal marker, respectively, a ~21% and 34% improvement, respectively, on our previous method).

Decision Points and Alternatives to the K-means test: Using the k-means test alone worked effectively for profiles that were acutely asymmetric but profiles do not always conform to this. In fact, we found that when the "orientation channel" had two peaks of similar amplitude the k-means algorithm fidelity decreased to 65% ± 15.1%, Mean ± SEM. Thus, we designed the program to test the "appropriateness" of the k-means test prior to orienting any profiles. All profiles with two peaks (in the 30% and 70% position of the profile) and had similar amplitudes (within 75% of the max peak) were counted and if they represented > 60% of the total then the "Derivative Test" was performed instead. One can also default to several different tests which simplify the orientation algorithm's decisions. These various tests can be found in the ***LAMPro Directions*** section.

Worm Profile Exclusion criteria

(1) *Size:* Debris and eggs were excluded by default. When bleached eggs were run though the sorter alone, all profiles were found to be below time of flight (TOF) = 75 thus, only animals above that TOF were considered. If data from only gated adult worms was saved then "debris and eggs" usually comprised < 2% of data. For other staged worms, which were also gated by size, "debris and eggs" comprised the following percentages, Mean ± SEM, L1:45 ± 5%, L2:5 ± 2%, L3:2 ± 2%, L4:1 ± 1%, D1:2 ± 3%, and D2:3 ± 3%, all *n*=4 biological replicates. N.B. If animals were not gated by size, the percent of "debris and eggs" was higher (e.g. at D1:18 ± 0.01%, Mean ± SEM, *n*=18 biological replicates).

(2) *Background Fluorescence:* Worms that had total fluorescence below background fluorescence in any channel (queried in the software command prompt) were also excluded. We determined the background fluorescence levels empirically for all stages of worms by running unlabeled, staged, animals through the cytometer and then creating Tukey Plots for each fluorescent channel. Total background fluorescence levels were determined by finding the point where a Tukey Plot would deem a data point an "outlier" (e.g. above 88th percentile).

Background Fluorescence Cutoff Points Using Standard PMT Levels					
	Larval L1	Larval L2	Larval L3	Larval L4	Adult ≥D1
Green Channel PMT=300	33.6	37.7	40.9	49.6	56.6

Yellow Channel PMT=380	49.6	77.7	85.1	109.1	161.6
Red Channel PMT=380	33.2	38.1	41.9	53.2	57.9

(3) *Extinction Artefact:* Refer to **Figure 1A** for an ideal EXT profile. Worms that were folded or had bacteria stuck to the tail or head (which both register as a high EXT and can influence TOF and the fluorescence profiles) (**Figure S1C-D**) were excluded by eliminating any profiles which were 1 SD above an average regression computed from the PH EXT on the Edges (y) and Middle (x) of the worm (**Figure S1E**). On average, this exclusion eliminated 17 ± 2%, Mean ± SEM (*n*=30 biological replicates) and greatly reduced the EXT variability on the edges of the profiles (**Figure S1F-H**). Notably, when using the 96-well plate reader (Reflex), the average exclusion was much higher than running animals through the cup, 63 ± 1%, Mean ± SEM (*n*=46).

Data Presentation (LAMPro GUI)

A short description of some of the applications and data analysis using oriented data from the ***LAMPro GUI*** software is provided below. A graphical user interface can be found in the supplement which should allow one to perform these functions using one program.

Median Profile Algorithm and Median Absolute Deviation (MAD): The median of each longitudinal value for all the worms within a file were plotted as a function of the percentage length along the worm the variability of this signal is plotted using median absolute deviation (MAD).

Stacking Profiles: Information from any set of profiles can be stacked in any order and compared (e.g. same heat scale) with other profiles.

Longitudinal Difference Plots, Significance Plots: Two median plots were compared by first taking the difference between the median longitudinal value for each point along the length of each worm and then plotting this difference (Δ) longitudinally. A Wilcoxon Rank Sum Test was calculated between each data set (for each 0.25% of the profile) along the length of the worm. Degree of significance was plotted such that all *P* values < 0.05 were plotted as numbers > "0". More specifically, "$-1.3-\log_{10}(P\text{-}value)$ " was plotted which distinguished regions of significance as above "0" ($P < 0.05$) and regions that were not significant ($P > 0.05$) as below "0" (see **Figure 2C**). With regard to a screen, we suggest a two-step process for analysis. (1) One has to observe differences (using a *Difference Plot*) between the median profiles in question and regard whether certain regional values are higher or lower than "control" animals. (2) One then can confirm the degree of significance of these differences by consulting the *Significance Plot*. Briefly, we wanted simplify the visualization of significance so we created a calculation that made all values ≥ 0 significant ($P \leq 0.05$). More specifically, a "$-1 .3-\log_{10}(P\text{-}value)$" was plotted which distinguished regions of significance by Wilcoxon Rank Sum Test. In situations where the observed difference is very small, because of the precision and sensitivity of the data visualization, one should be able to resolve even subtle differences using a *Significance Plot*.

Regional Significance: Using data from the significance plot function, the frequency of *P-values* of differing significance (*P* < 0.0001, 0.001, 0.01, 0.05, and not significant, N.S.) within a set region of the animal (i.e. 0-25%, 25-85%, and 85-100%) can be plotted in a stacked histogram (see **Figure 3C**, **S4F,** and **S5K**).

Saturation Plots: When screening it can be useful to know if a set of profile data has a large proportion of fluorescent values that are "maxed out" or above the maximum allowed fluorescent values for that particular channel can be plotted using the saturation plot. This function displays the percent of profiles within each data set that contained a Peak Height (PH) fluorescence that was "maxed out" (**Figure S3D**). The saturated regions can be characterized using the "saturation plot" function which displays, like a median profile plot, the frequency of "maxed out" points along the animal (**Figure S3E-F**).

WORKFLOWS FOR DATA PROCESSING AND LAMPRO ALGORITHM

Generalized Workflow for Data Processing

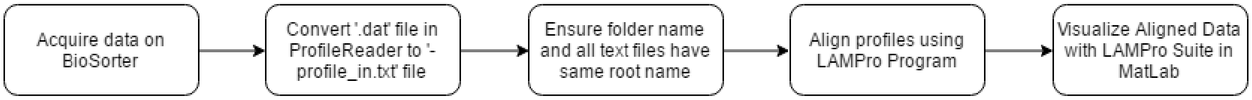

Workflow for LAMPro Algorithm

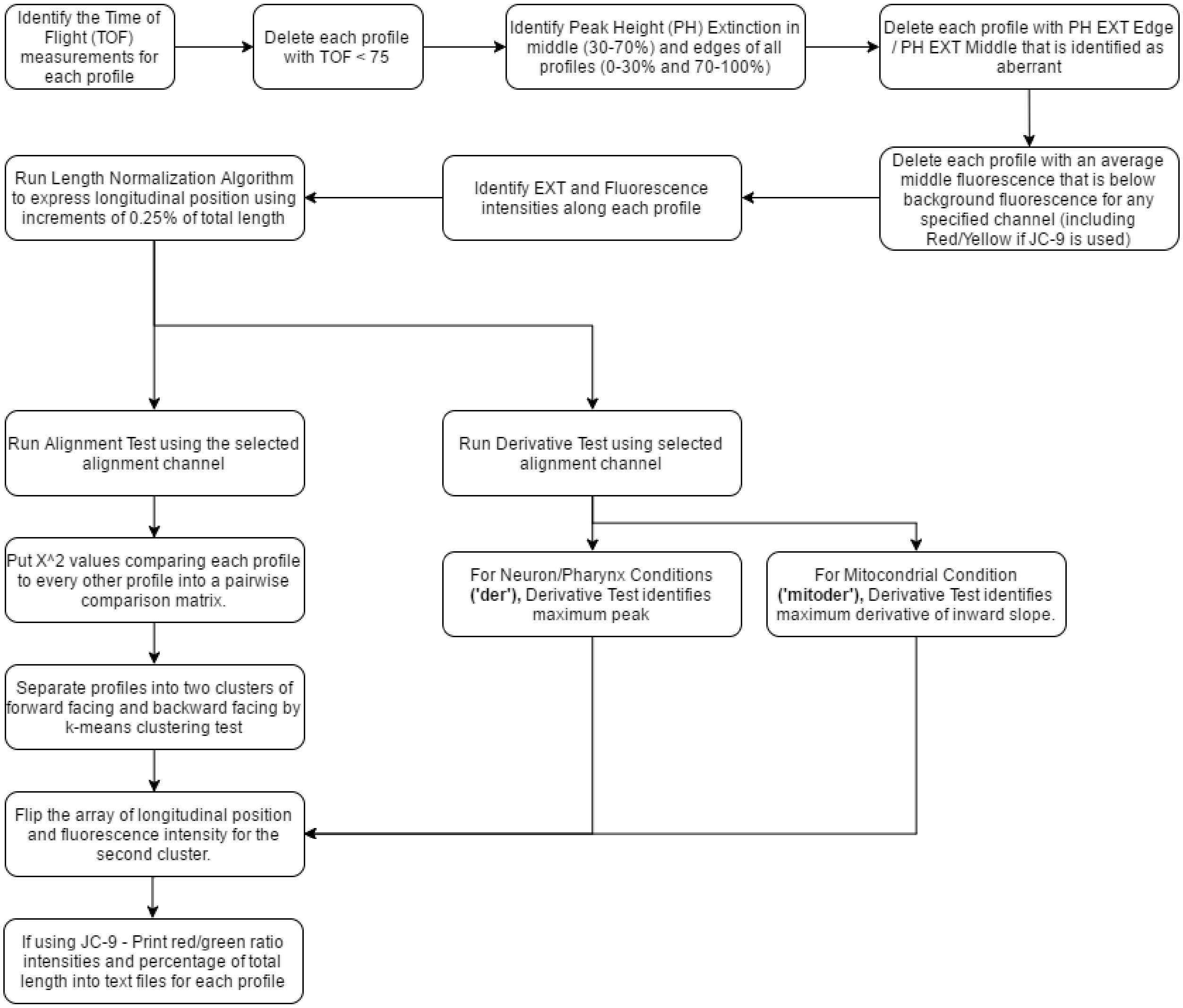

SUPPLEMENTAL REFERENCES

1. Chai, Y. *et al.* Live imaging of cellular dynamics during Caenorhabditis elegans postembryonic development. *Nat. Protoc.* **7,** 2090–2102 (2012).
2. Zeiser, E., Frøkjær-Jensen, C., Jorgensen, E. & Ahringer, J. MosSCI and Gateway Compatible Plasmid Toolkit for Constitutive and Inducible Expression of Transgenes in the C. elegans Germline. *PLoS ONE* **6,** e20082 (2011).
3. Daniele, J. R., Heydari, K., Arriaga, E. A. & Dillin, A. Identification and Characterization of Mitochondrial Subtypes in Caenorhabditis elegans via Analysis of Individual Mitochondria by Flow Cytometry. *Anal. Chem.* **88,** 6309–6316 (2016).
4. Lloyd, S. Least Squares Quantization in PCM. *IEEE Trans Inf Theor* **28,** 129–137 (2006).
5. Arthur, D. & Vassilvitskii, S. K-means++: The Advantages of Careful Seeding. in *Proceedings of the Eighteenth Annual ACM-SIAM Symposium on Discrete Algorithms* 1027–1035 (Society for Industrial and Applied Mathematics, 2007).

SUPPLEMENTARY FIGURE LEGENDS

Figure S1. Specifics of longitudinal alignment algorithm. (A-B) Length standardization algorithm retains the positional information and profile fingerprint regardless of "expansion" **(A)** or "distillation" **(B)** of individual profiles. All profiles are "transformed" to become 400 bit profiles. 'A.F.U.' = Arbitrary Fluorescence Units. **(C)** (Top) Median profiles derived from *C. elegans* labeled with GFP-positive bacteria to correlation between the MAD (Bottom) of bacteria on the edges of worms and Extinction (EXT). Plots of Pharynx and Intestine are also shown (Top) to depict the location and edges of the mouth and digestive system, respectively. **(D)** Representative micrographs of worms fed GFP-labeled bacteria (as in **C**) showing common places where bacteria aggregate. Worms are oriented with anterior to the left. "ANT." = Anterior; "POST." = Posterior; 'TL' = Transillumination. Scale = 200 µm. **(E-E'')** Demonstration of exclusion algorithm to eliminate 'non-ideal' profiles with high EXT on the edges across various developmental stages of nematodes. Red crosses represent data points prior to exclusion (OFF) while blue circles represent data after exclusion (ON). Regression represents a line that is one standard deviation above the 'average' regression derived from a relationship between Peak Height EXT on the 'edges' of profiles (20% on edges) vs. Peak Height (PH) EXT in the 'middle' of profiles (middle 60%). **(F)** Box plots of PH EXT on profile edges: PH EXT in the middle of profiles ratios for L1-D2 worms with (white boxes) and without (grey boxes) exclusion. **(G-H)** Individual profiles (Y axis) represented as in **(C)** with signal intensity plotted as a function of the animal's length (X axis). EXT of individual oriented profiles is displayed without **(G)** and with **(H)** exclusion criteria on. **(G'-H')** Median MAD of oriented profiles without **(G')** and with **(H')** exclusion criteria on. All animals are positioned with the anterior side to the left. Plots are representative of three technical replicates and derived from $n \geq 50$ organisms unless otherwise stated. **P<0.001 and ***P<0.0001 by Wilcoxon Rank Sum Test. Sample size (n) for all experiments and exact *P-values* can be found in the Supplementary Table.

Figure S2. Supplement to tissue expression data. (A) Representative micrographs of transgenic worms analyzed in **Figure 2A-C** with tissues (Red) and sensory neurons (Green) fluorescently labeled. Worms are orientated with their anterior to the left. "ANT.' = Anterior; 'POST.' = Poster; 'TL' = Transillumination. Scale = 200 µm. **(B-C)** Demonstration of orientation algorithm showing individual profiles (Y axis) represented with signal intensity plotted as a function of the animal's length (X axis). Sensory neurons have been labeled (DiO in **B** and DiI in **C**) to enable orientation. Data is shown in a heat map with Red highest intensity signal and Blue as the lowest intensity. **(D)** Median fluorescence (or extinction) intensities (top) and corresponding median absolute deviation (MAD) plots (below). **(E)** Median profiles derived from a germline/egg specific GFP transgenic worm (VIT-2::GFP) showing location of eggs/germline at different developmental stages in worm. Worms were oriented using DiI signal. **(F)** Representative micrographs of worms shown in **(E)** with gonad and eggs (Green) and sensory neurons (Red). "ANT." = Anterior; "POST." = Posterior. Scale = 200 µm. **(G)** Representative micrographs of the germlines of live-labeled nematodes with JC-9. Labeled mitochondria (JC-9 Monomer, Yellow), polarized mitochondria (JC-9 Aggregate, Red) 'TL' = Transillumination. Scale bar is 50 µm. Sample size (n) for all experiments can be found in the Supplementary Table.

Figure S3. Supplement to transcriptional reporter data. (A) Quantification of reporter fluorescence after induction of the mitochondrial unfolded protein response (UPR^{mt}) by various RNAi treatments. White boxes are with exclusion criteria 'ON' (to eliminate low quality profiles) and grey boxes are with exclusion criteria 'OFF'. Unless otherwise labeled, all fluorescence values were significantly different (P<0.00001) when compared to "control" or to "*cco-1* RNAi" by Wilcoxon Rank Sum in both the

exclusion "ON" and the "OFF" settings. "n.s."= not significant. **(B)** Representative micrographs of transcriptional mitochondrial unfolded protein response (UPR^{mt}) reporter worms (*hsp-6p*::GFP, analyzed in **Figure 3**) with increased GFP representing induction of UPR^{mt} (Green) and sensory neurons labeled with DiI (Red). Worms are oriented with anterior to the left. "ANT." = Anterior; "POST." = Posterior; 'TL' = Transillumination. Scale = 200 µm. **(C)** Median absolute deviation (MAD) plot derived from median profiles seen in **Figure 3A**. All plots are positioned with the anterior side to the left. Plots are representative of three technical replicates and derived from $n \geq 50$ organisms unless otherwise stated. **(D)** Saturation plot displaying the percentage of profiles from each data set that contain a peak height fluorescence measurement that is at the 'max' intensity. White bars represent PMT settings that are too high in sensitivity; black bars represent PMT settings that accommodate the dynamic range of the reporter. **(E-F)** Spatial distribution for two RNAi treatments (*spg-7* and *tim-17(1)* RNAi) where profiles are saturated under 'high' (**S3E**) and 'optimized' (**S3F**) PMT sensitivities. Sample size (*n*) for all experiments and exact *P-values* can be found in the Supplementary Table.

Figure S4. Representation of live labeling of mitochondria and membrane potential in *C. elegans*. **(A)** Representative micrographs of live-labeled nematodes using JC-9 (Green) and mitochondrially-targeted blue-fluorescent protein (MLS::BFP, Blue) expressed in various tissues. Scale bar is 10 µM. **(B-C)** Quantification of mitochondrial membrane potential using the JC-9 monomeric (Yellow, **S3B**) and aggregate (Red, **S3C**) average fluorescence from dye-labeled nematodes with (ON, white boxes) and without (OFF, grey boxes) exclusion criteria on. Signal from aggregates (Red, **S3C**) decrease when worms are treated with the depolarizing agent valinomycin. ****P<0.00001 by Wilcoxon Rank Sum Test. **(C**) Representative micrographs of live-labeled nematodes with JC-9 under polarized and depolarized conditions. Labeled mitochondria (JC-9 Monomer, Yellow), polarized mitochondria (JC-9 Aggregate, Red), and membrane potential (Red signal/ Yellow signal) are shown. The magnitude of membrane potential is represented with the highest values in white and the lowest values in black. 'TL' = Transillumination. Scale bar is 200 µm. **(D)** Median absolute deviation (MAD) plot derived from the median profiles of various *C. elegans* lines in **Figure 3B**. **(E)** Frequency plots for the percentage of bits in each region of the worm (left) that correspond to varying degrees of significance (white = not significant 'N.S.'; light grey = P<0.005; medium grey = P<0.01; dark grey = P<0.001; black = P<0.0001). Comparisons are made for each of these regions between polarized and depolarized data sets. All animals are positioned with the anterior side to the left. Plots are representative of three technical replicates and derived from $n \geq 50$ organisms unless otherwise stated. Sample size (*n*) for all experiments and exact *P-values* can be found in the Supplementary Table.

Figure S5. Supplement to developing *C. elegans* data set. (A) Representative micrographs of live-labeled nematodes using JC-9 at various stages of early development. Labeled mitochondria (JC-9 Monomer, Yellow), polarized mitochondria (JC-9 Aggregate, Red), and membrane potential (Red signal / Yellow signal) are shown. The magnitude of membrane potential is represented with the highest values in white and the lowest in black. 'TL' = Transillumination. Scale bar is 200 µm. **(B,C)** Tukey box plots of median time of flight (TOF) and extinction (EXT) for various developmental stages of nematode. A Wilcoxon Rank Sum tests was performed between adjacent stages (e.g. L1 to L2) unless otherwise labeled. ****P<0.00001 and "n.s." = not significant. **(D)** Illustration of process to define JC-9 monomer binding pattern to early developmental stages of *C. elegans*. (Top) Line scan of JC-9 monomer signal intensity taken from 5 representative micrographs of L4 *C. elegans* and plotted as a function of nematode length. (Bottom) Representative median biosorter profile from the yellow channel (JC-9 monomer) signal intensity of 25 individual L4 nematodes plotted against length. Error bars are median absolute deviation (MAD). All animals are positioned with the anterior side to the left. **(E-G)** Demonstration of orientation

algorithm showing individual profiles (Y axis) represented with signal intensity plotted as a function of the animal's length (X axis). Yellow (monomeric JC-9) fluorescence is shown which enables orientation in **(E)** larval L1, **(F)** larval L4, and **(G)** D2 adult *C. elegans*. Data is shown in a heat map with Red as highest intensity signal and Blue the lowest intensity. **(H)** Median profiles from animals in **(B)** generated by plotting JC-9 ratio ($\Delta\psi$) against developmental stage. **(I)** Median absolute deviation (MAD) plot derived from median profiles in **Figure S5H**. **(J)** Difference (Δ) plots derived from median profiles in **Figure S5H** comparing each profile to its previous stage (e.g. L1 – L2). **(K)** Frequency plots for the percentage of bits in each region of the worm (left) that correspond to varying degrees of significance (white = not significant 'N.S.'; light grey = P<0.005; medium grey = P<0.01; dark grey = P<0.001; black = P<0.0001). Comparisons are made for each profile to its previous stage for all these regions. All plots are positioned with the anterior side to the left. Plots are representative of three technical replicates and derived from $n \geq 50$ organisms unless otherwise stated. Sample size (*n*) for all experiments and exact *P-values* can be found in the Supplementary Table.

Figure S6. Supplement to *C. elegans* longevity model data. (A) Representative micrographs of L4 staged long-lived mutants that have been live-labeled with JC-9. Labeled mitochondria (JC-9 Monomer, Yellow), polarized mitochondria (JC-9 Aggregate, Red), and membrane potential (Red signal / Yellow signal) are shown. The magnitude of membrane potential is represented with the highest values in white and the lowers in black. 'TL' = Transillumination. Scale bar is 200 µm. **(B)** Median absolute deviation (MAD) plot derived from median profiles seen in **Figure 6B**. All plots are positioned with the anterior side to the left. Plots are representative of three technical replicates and derived from $n \geq 50$ organisms unless otherwise stated. Sample size (*n*) for all experiments can be found in the Supplementary Table.

Figure S7. Supplement to *C. elegans* mitochondrial dynamics measurements. (A) Representative micrographs of transgenic nematodes using matrix-localized MLS::BFP (Blue) (as in **Figure S4A**) and muscle-specific mitochondrially-targeted (using a different MLS) green-fluorescent protein (MLS2::GFP) expressed in muscle tissues. 'TL' = Transillumination. Scale bar is 10 µM. **(B,C)** Tukey box plots of median **(B)** Time of Flight (TOF) and **(C)** Green fluorescence when 20,000+ genotypically identical nematodes with green fluorescent muscle mitochondria (*myo-3p::MLS2:::GFP*) and red fluorescent pharynx (*myo-2p::mCherry*, to enable alignment) were run and analyzed using our 96-well plate program. To facilitate visualization and minimize cross-well contamination animals were plated in every other well. **(D,E)** Median plots from the **(D)** red alignment channel and the **(E)** green muscle mitochondria. **(F-H)** Tukey box plots of **(D)** Green fluorescence (mitochondrial mass), **(E)** TOF, and **(F)** Extinction of animals under various RNAi treatments against genes known to be important in the maintenance of mitochondrial morphology. **(I)** Median green fluorescence and **(J)** median absolute deviation (of green signal) for RNAi treatments in **F-H** demonstrate spatial distribution of muscle mitochondrial signal. **(K)** Significance ($\log_{10}$(*P-value*)) plots derived from median profiles in **(I)** comparing each profile to "control" or "empty vector" treated nematodes. For significance plots a value ≥ 0 is equivalent to $P \leq 0.05$ by Wilcoxon Rank Sum Test. Sample size (*n*) for all experiments and exact *P-values* can be found in the Supplementary Table.

Figure S8. Supplement to *C. elegans* mitochondrial morphology mutant characterization. (A-C) Tukey box plots of **(A)** Green fluorescence and **(B)** TOF for several select genes when exclusion criteria are turned off. Differences in Green fluorescence are lost for *C24H11.6* (*immp-1)* and *W02B12.9* (*mfn-1)* treatments and the TOF measurements are also marked different from data with exclusion. **(C)** Median absolute deviation (MAD) plot derived from median profiles seen in **Figure 7A**. All animals are

positioned with the anterior side to the left. Plots are representative of two technical replicates and derived from $n \geq 100$ organisms unless otherwise stated. **(D-G)** Micrographs of muscle-specific, matrix-localized MLS2::GFP ("mito::GFP") signal across an entire representative animal for **(D)** control (empty vector), **(E)** *immp-1* RNAi, **(F)** *mfn-1* RNAi, and **(G)** *W108.5* RNAi treatments. Scale bar is 50 µm. The '*' in **(E)** marks areas that deviates significantly from control. Colored boxes in **D-G** represent areas within the animal, identified in **(C)**, where median profiles deviate significantly from controls. **(G)** Fluorescence on the perimeter of concentric circles was plotted moving radially from the nucleus for control (black, empty vector) and *immp-1* RNAi (red) nematodes. **(H)** Median integrated fluorescence of the perinuclear region in control (black) and *immp-1* RNAi (red) expressing nematodes. **(J)** Tukey box plots of mitochondrial mass by JC-9 monomeric (Yellow) signal. Wilcoxon Rank Sum tests were performed against "empty vector" controls. $^{**}P<0.01$ and "Ω" = $P<0.00001$. **(K)** Median membrane potential ($\Delta\psi$, JC-9 Yellow/Red ratio) and median absolute deviation (MAD) derived from **(K)** are seen in **(L)**. **(M)** Difference in ratio vs. control along each median profile is also plotted. Sample size (n) for all experiments and exact *P-values* can be found in the Supplementary Table.

SUPPLEMENTARY FIGURES

Figure S1

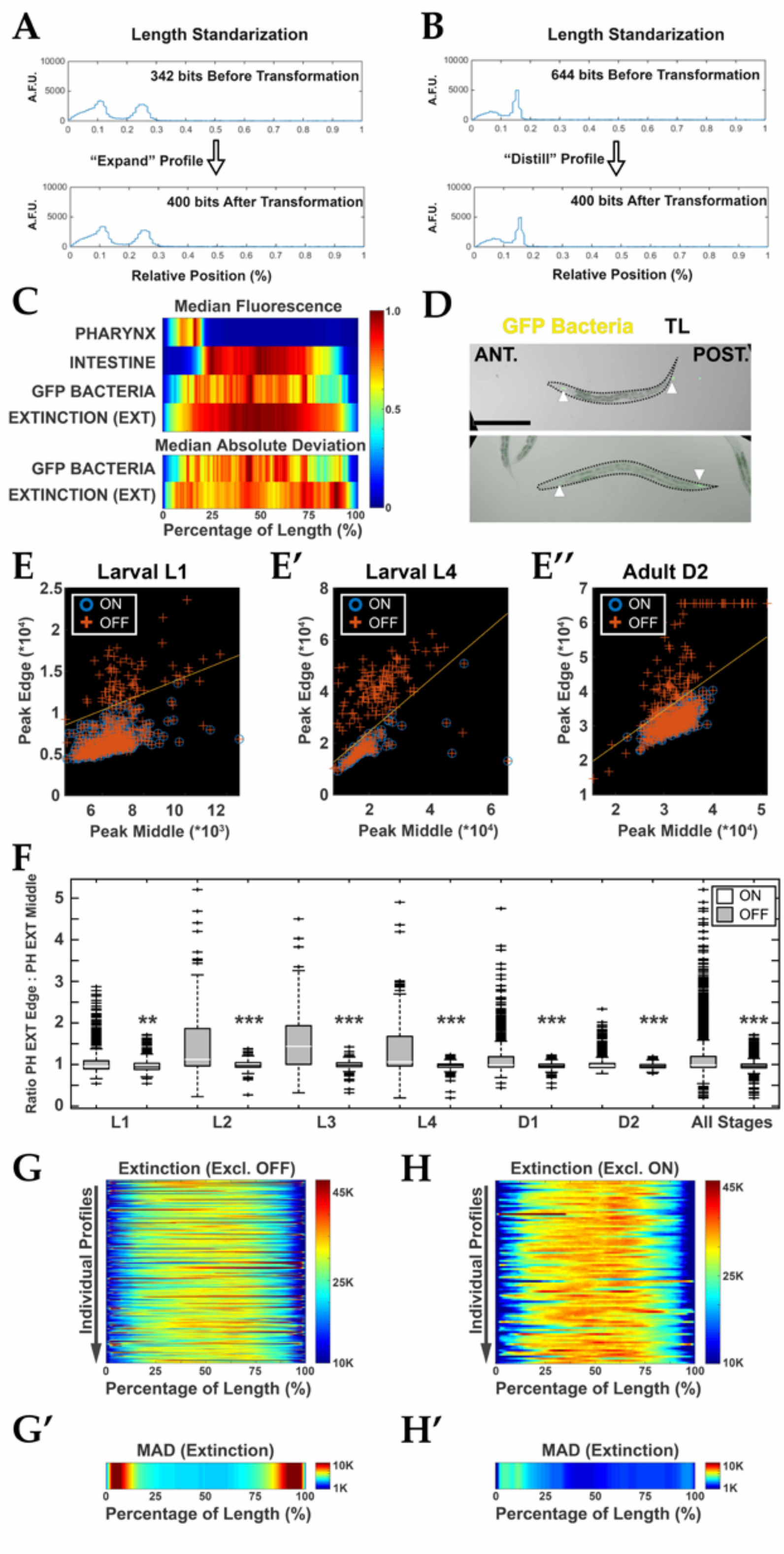

Figure S2

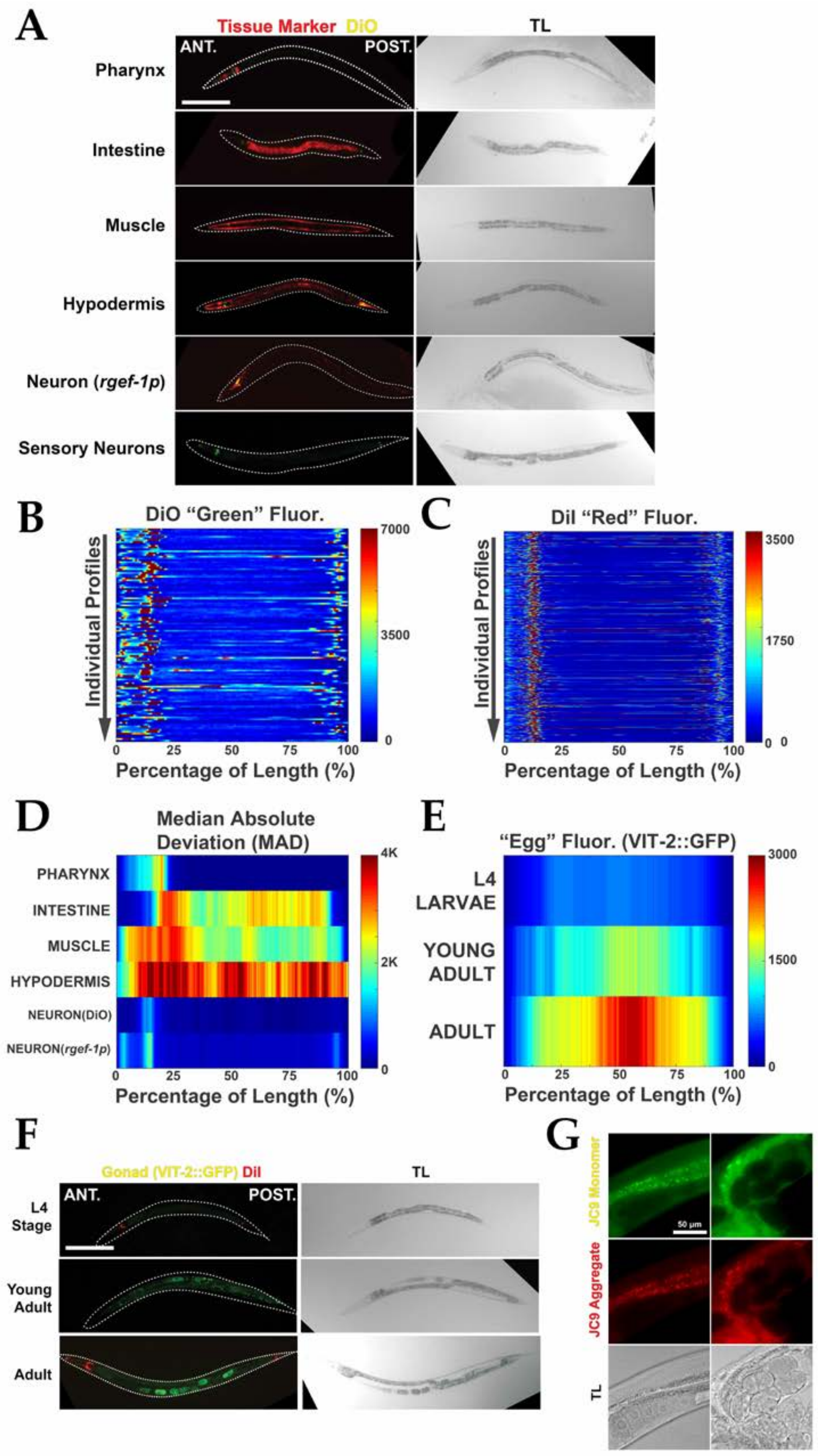
A
Tissue Marker DiO
TL
ANT.
POST.
Pharynx
Intestine
Muscle
Hypodermis
Neuron (rgef-1p)
Sensory Neurons
B
DiO "Green" Fluor.
Individual Profiles
Percentage of Length (%)
C
DiI "Red" Fluor.
D
Median Absolute Deviation (MAD)
PHARYNX
INTESTINE
MUSCLE
HYPODERMIS
NEURON(DiO)
NEURON(rgef-1p)
E
"Egg" Fluor. (VIT-2::GFP)
L4 LARVAE
YOUNG ADULT
ADULT
F
Gonad (VIT-2::GFP) DiI
L4 Stage
Young Adult
Adult
G
JC9 Monomer
JC9 Aggregate
50 μm

Figure S3

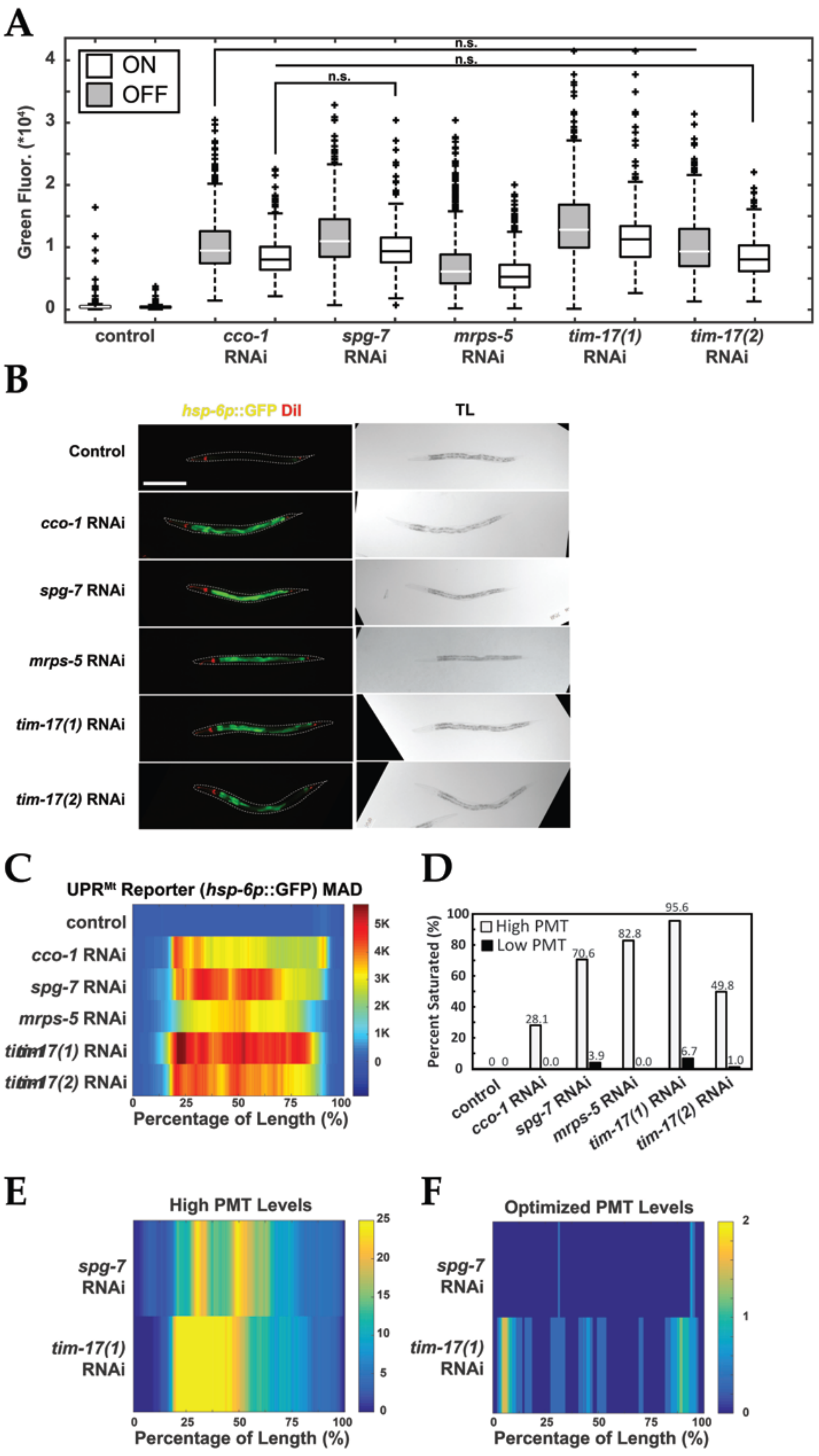
A
ON
OFF
n.s.
n.s.
n.s.
Green Fluor. (*10⁴)
control
cco-1 RNAi
spg-7 RNAi
mrps-5 RNAi
tim-17(1) RNAi
tim-17(2) RNAi
B
hsp-6p::GFP DiI
TL
Control
cco-1 RNAi
spg-7 RNAi
mrps-5 RNAi
tim-17(1) RNAi
tim-17(2) RNAi
C
UPRMt Reporter (hsp-6p::GFP) MAD
control
cco-1 RNAi
spg-7 RNAi
mrps-5 RNAi
tim-17(1) RNAi
tim-17(2) RNAi
Percentage of Length (%)
D
High PMT
Low PMT
Percent Saturated (%)
28.1
70.6
82.8
95.6
49.8
3.9
6.7
1.0
control
cco-1 RNAi
spg-7 RNAi
mrps-5 RNAi
tim-17(1) RNAi
tim-17(2) RNAi
E
High PMT Levels
spg-7 RNAi
tim-17(1) RNAi
Percentage of Length (%)
F
Optimized PMT Levels
spg-7 RNAi
tim-17(1) RNAi
Percentage of Length (%)

Figure S4

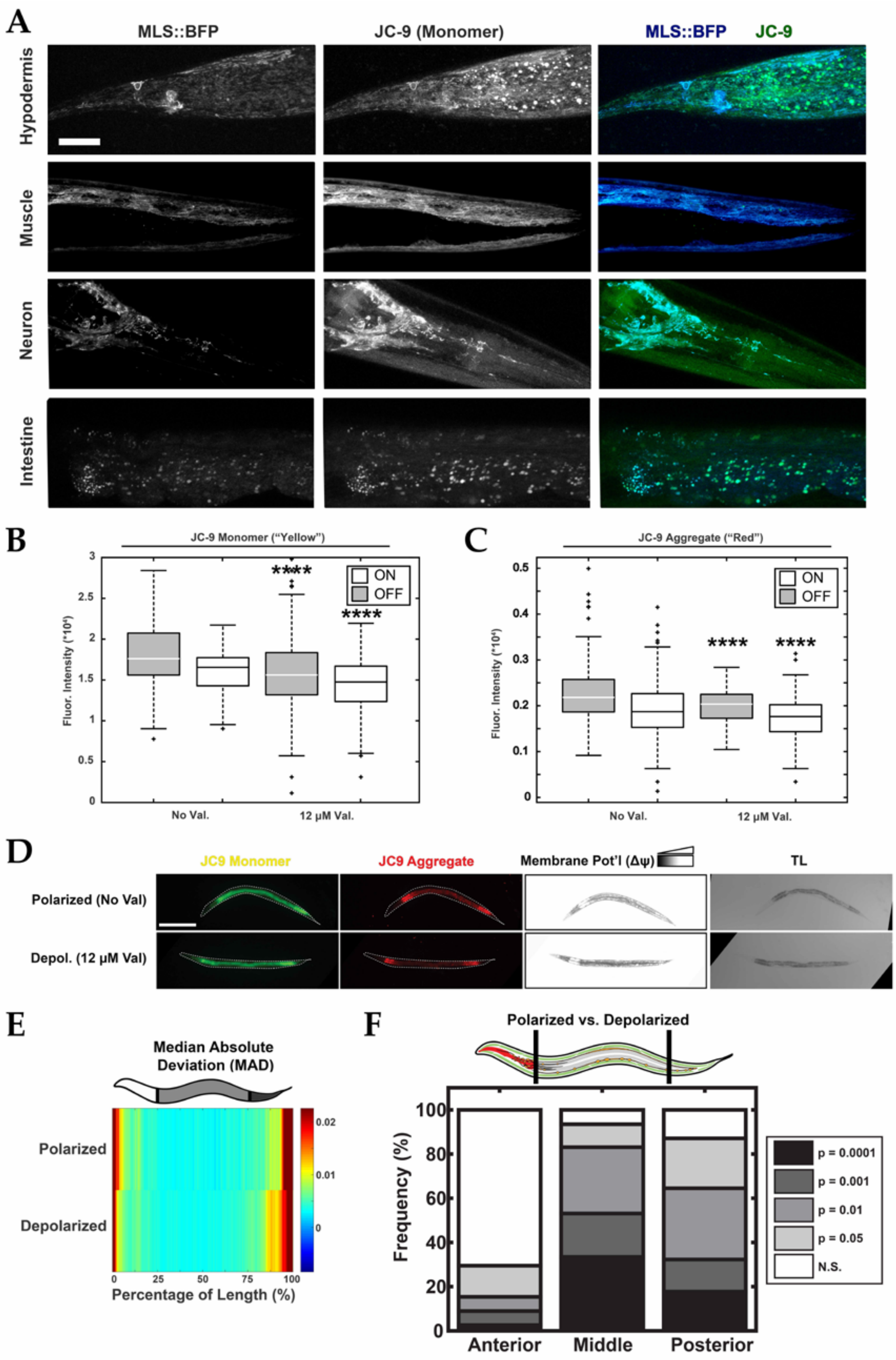
A
MLS::BFP
JC-9 (Monomer)
MLS::BFP JC-9
Hypodermis
Muscle
Neuron
Intestine
B
JC-9 Monomer ("Yellow")
Fluor. Intensity (*10⁴)
ON
OFF
No Val.
12 μM Val.
C
JC-9 Aggregate ("Red")
D
JC9 Monomer
JC9 Aggregate
Membrane Pot'l (Δψ)
TL
Polarized (No Val)
Depol. (12 μM Val)
E
Median Absolute Deviation (MAD)
Polarized
Depolarized
Percentage of Length (%)
F
Polarized vs. Depolarized
Frequency (%)
p = 0.0001
p = 0.001
p = 0.01
p = 0.05
N.S.
Anterior
Middle
Posterior

Figure S5

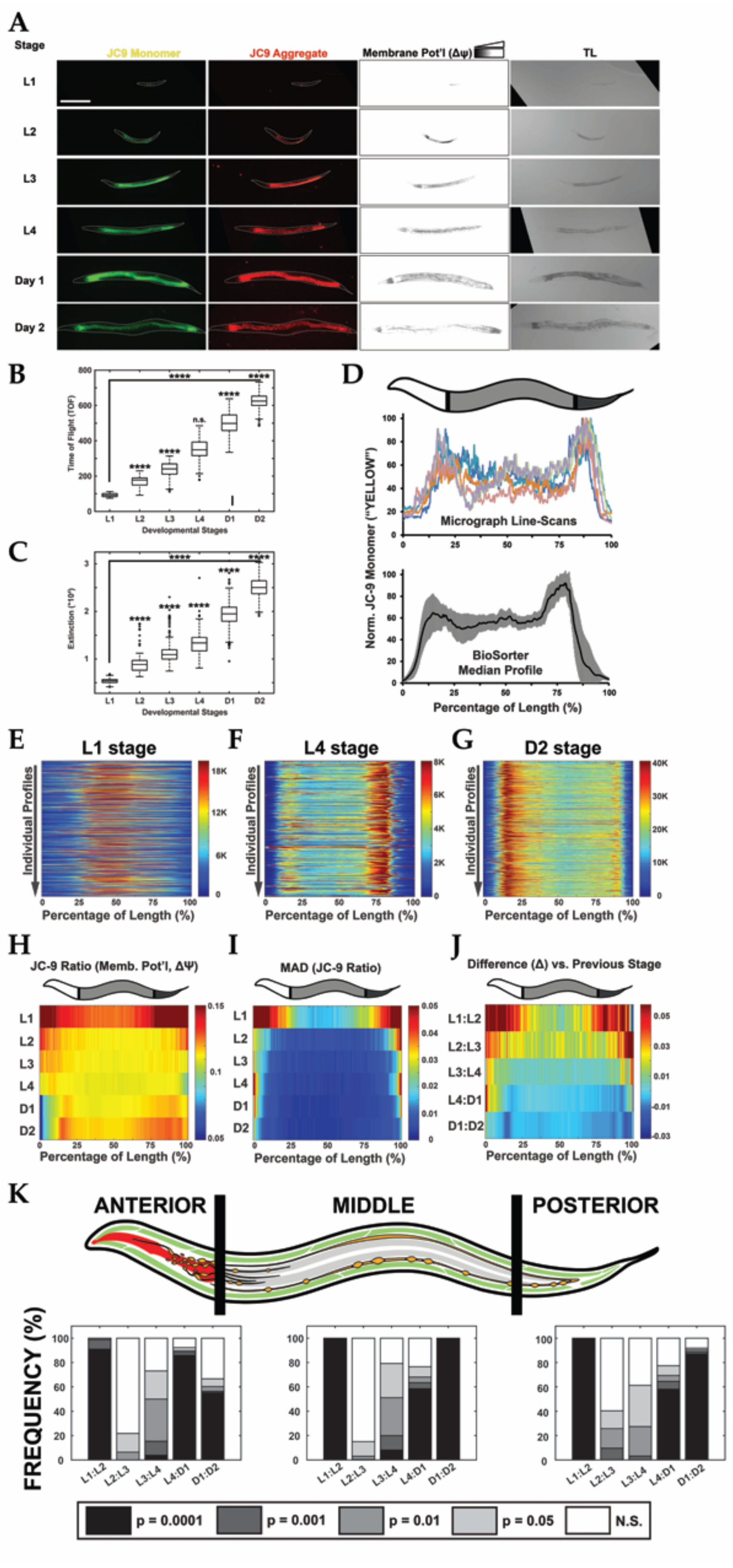
A
Stage
JC9 Monomer
JC9 Aggregate
Membrane Pot'l (Δψ)
TL
L1
L2
L3
L4
Day 1
Day 2
B
Time of Flight (TOF)
Developmental Stages
C
Extinction
D
Norm. JC-9 Monomer ("YELLOW")
Micrograph Line-Scans
BioSorter
Median Profile
Percentage of Length (%)
E
L1 stage
F
L4 stage
G
D2 stage
Individual Profiles
Percentage of Length (%)
H
JC-9 Ratio (Memb. Pot'l, ΔΨ)
I
MAD (JC-9 Ratio)
J
Difference (Δ) vs. Previous Stage
K
ANTERIOR
MIDDLE
POSTERIOR
FREQUENCY (%)
p = 0.0001
p = 0.001
p = 0.01
p = 0.05
N.S.

Figure S6

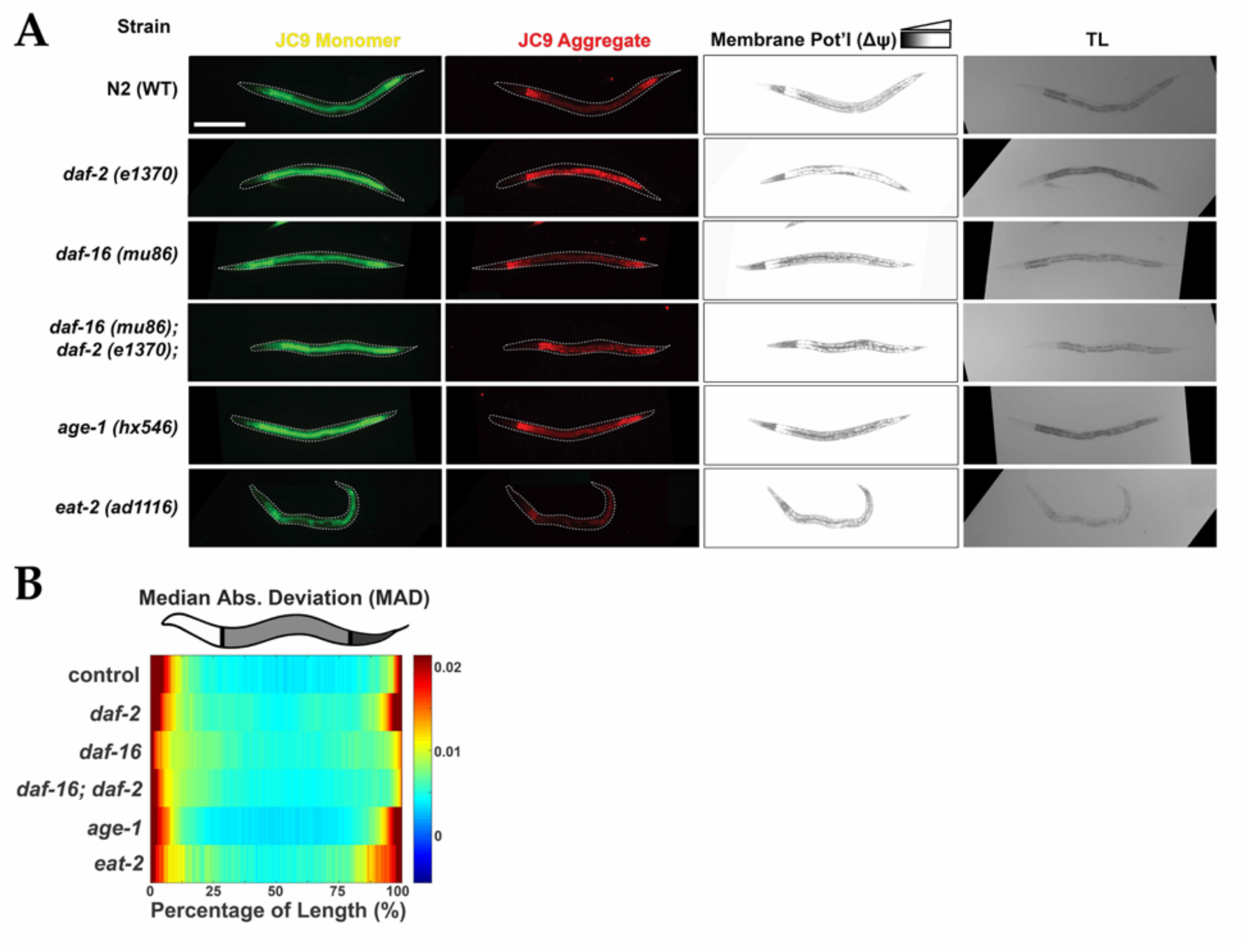
A
Strain
JC9 Monomer
JC9 Aggregate
Membrane Pot'l (Δψ)
TL
N2 (WT)
daf-2 (e1370)
daf-16 (mu86)
daf-16 (mu86);
daf-2 (e1370);
age-1 (hx546)
eat-2 (ad1116)
B
Median Abs. Deviation (MAD)
control
daf-2
daf-16
daf-16; daf-2
age-1
eat-2
0.02
0.01
0
0
25
50
75
100
Percentage of Length (%)

Figure S7

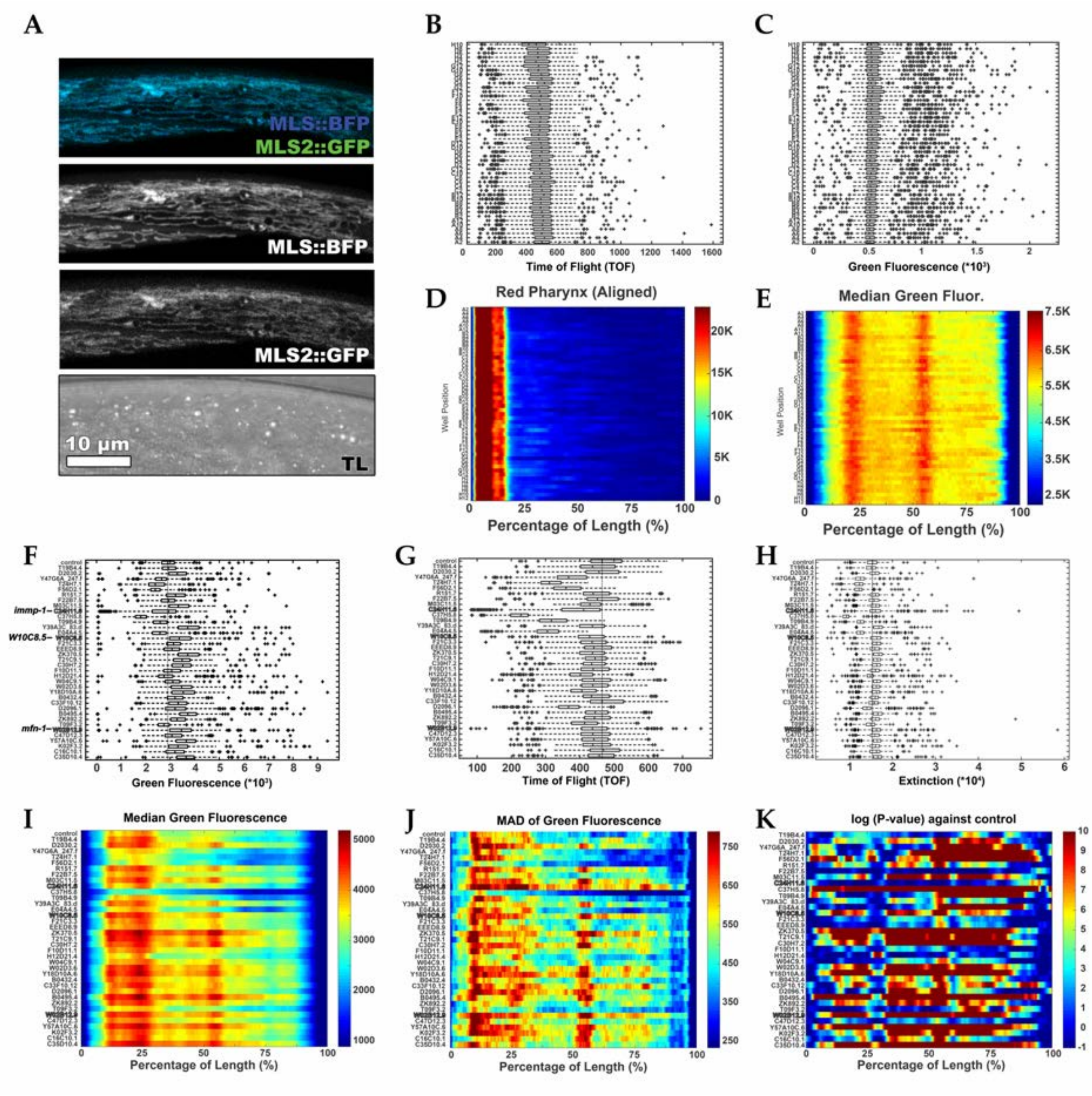
A
MLS::BFP
MLS2::GFP
MLS::BFP
MLS2::GFP
10 μm
TL
B
Time of Flight (TOF)
C
Green Fluorescence (*10³)
D
Red Pharynx (Aligned)
Well Position
Percentage of Length (%)
E
Median Green Fluor.
Well Position
Percentage of Length (%)
F
immp-1
W10C8.5
mfn-1
Green Fluorescence (*10³)
G
Time of Flight (TOF)
H
Extinction (*10⁴)
I
Median Green Fluorescence
Percentage of Length (%)
J
MAD of Green Fluorescence
Percentage of Length (%)
K
log (P-value) against control
Percentage of Length (%)

Figure S8

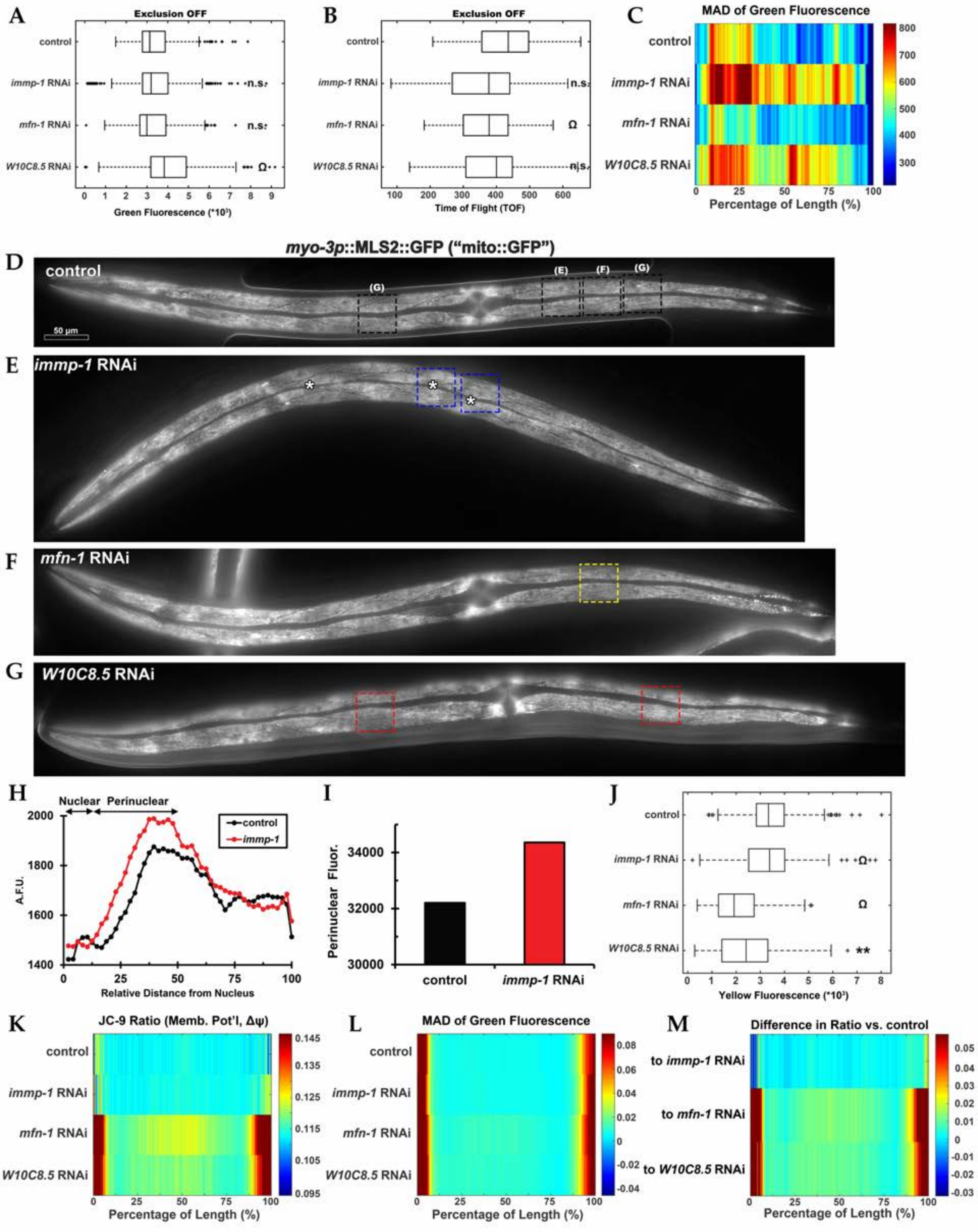
A
Exclusion OFF
control
immp-1 RNAi
mfn-1 RNAi
W10C8.5 RNAi
n.s.
n.s.
Ω
Green Fluorescence (*10³)
B
Exclusion OFF
n.s.
Ω
n.s.
Time of Flight (TOF)
C
MAD of Green Fluorescence
Percentage of Length (%)
myo-3p::MLS2::GFP ("mito::GFP")
D
control
50 μm
E
immp-1 RNAi
F
mfn-1 RNAi
G
W10C8.5 RNAi
H
Nuclear
Perinuclear
control
immp-1
A.F.U.
Relative Distance from Nucleus
I
Perinuclear Fluor.
control
immp-1 RNAi
J
Yellow Fluorescence (*10³)
**
K
JC-9 Ratio (Memb. Pot'l, Δψ)
Percentage of Length (%)
L
MAD of Green Fluorescence
Percentage of Length (%)
M
Difference in Ratio vs. control
to immp-1 RNAi
to mfn-1 RNAi
to W10C8.5 RNAi
Percentage of Length (%)

LAMPro DIRECTIONS

Step-By-Step Directions for Running the LAMPro Software

1. Place all files for the desired run into a folder labeled identically to the respective filename.txt

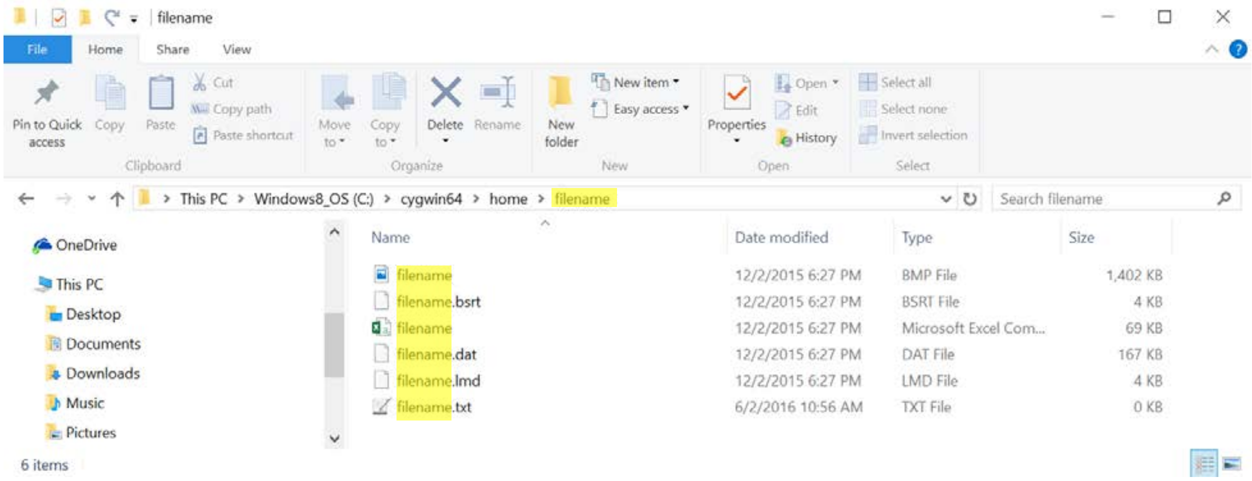

2. Obtain the longitudinal data
    a. Open ProfReader161.exe
    b. Open the desired filename.dat from the folder created in Step 1

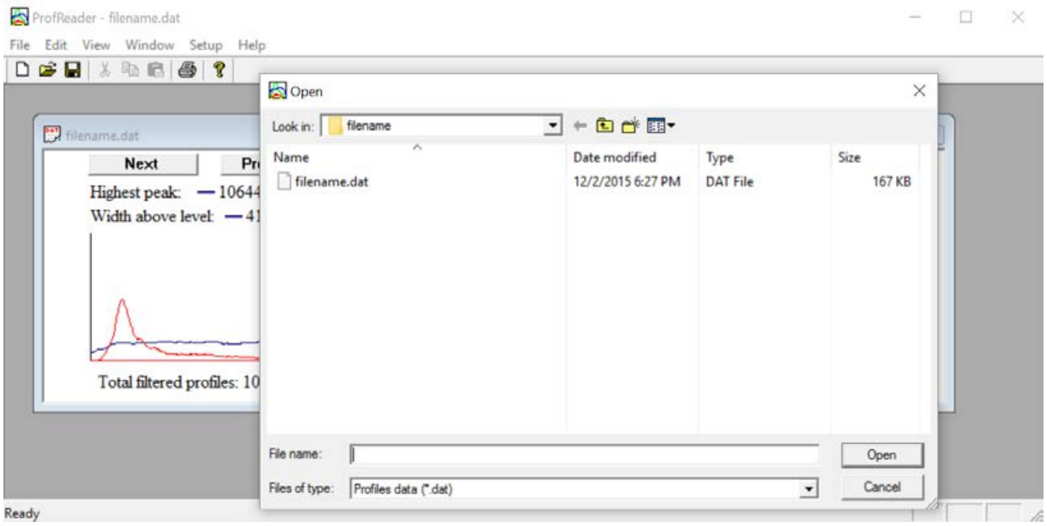

i. Double-check that all 4 channels (Extinction, Green, Yellow, Red) are saved while obtaining the data. The program will not work if the highlighted box does not have all 4 channels.

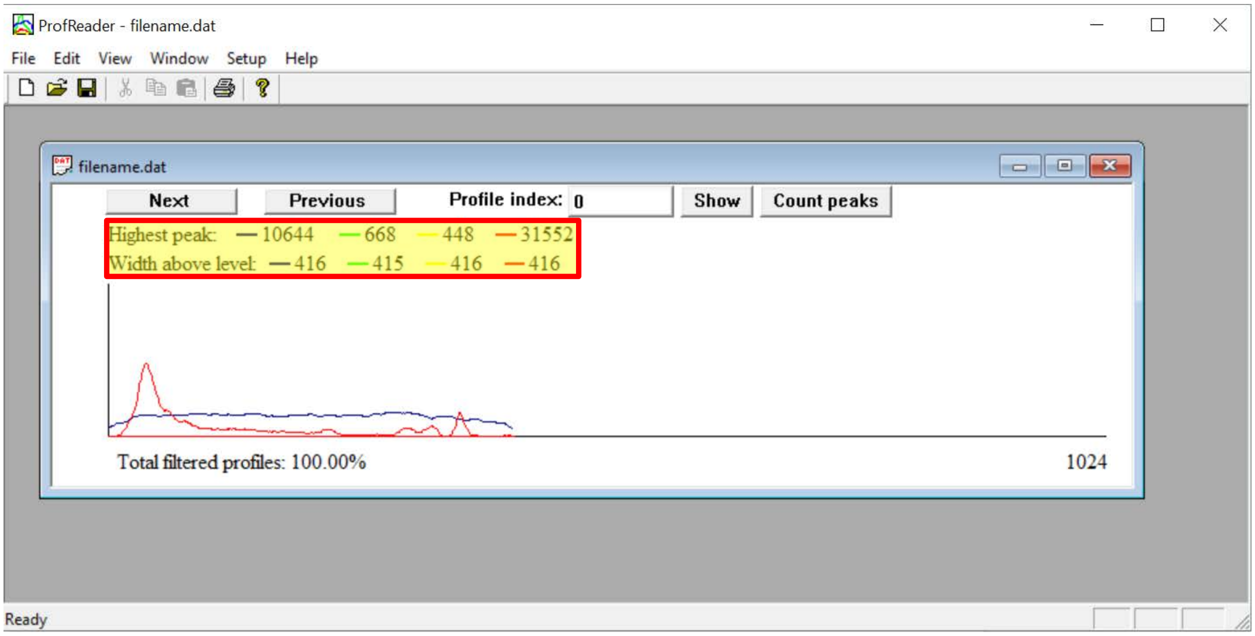

c. Find the number of profiles that you would like to export

i. If it is the maximum number of profiles for the experiment, type a large number into the Profile index box.

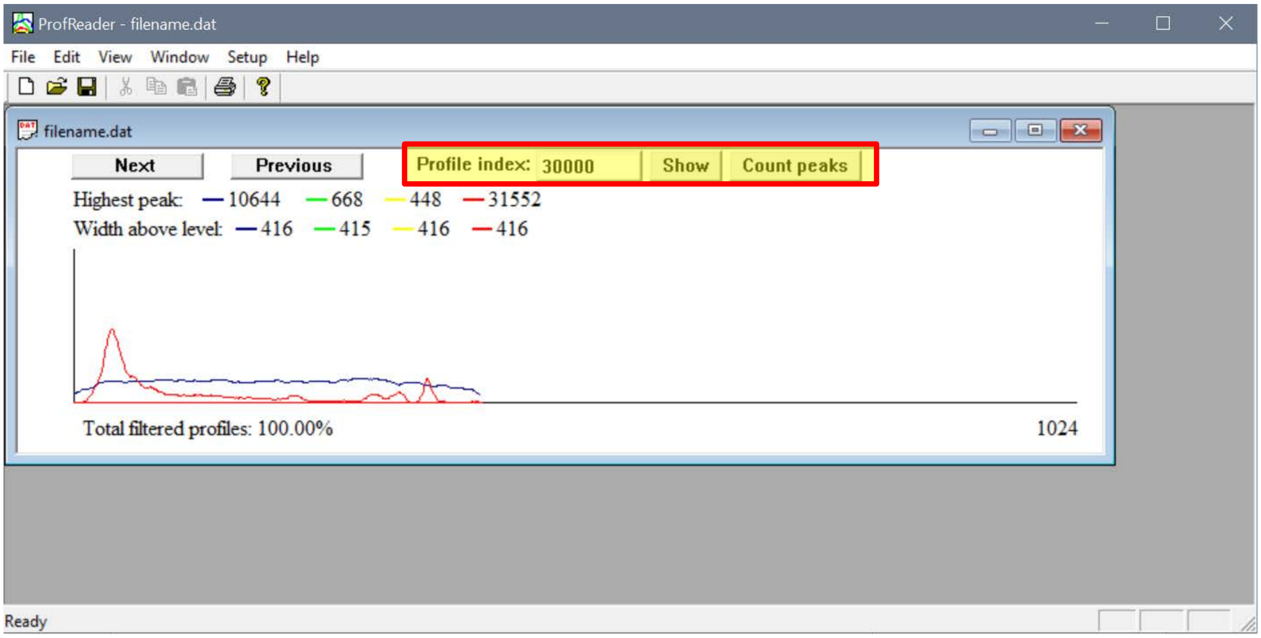

ii. Then, a dialogue box will show if you are over the maximum number of profiles, indicating the maximum number of profiles for the experiment. Increase the Profile Index number until this message is shown

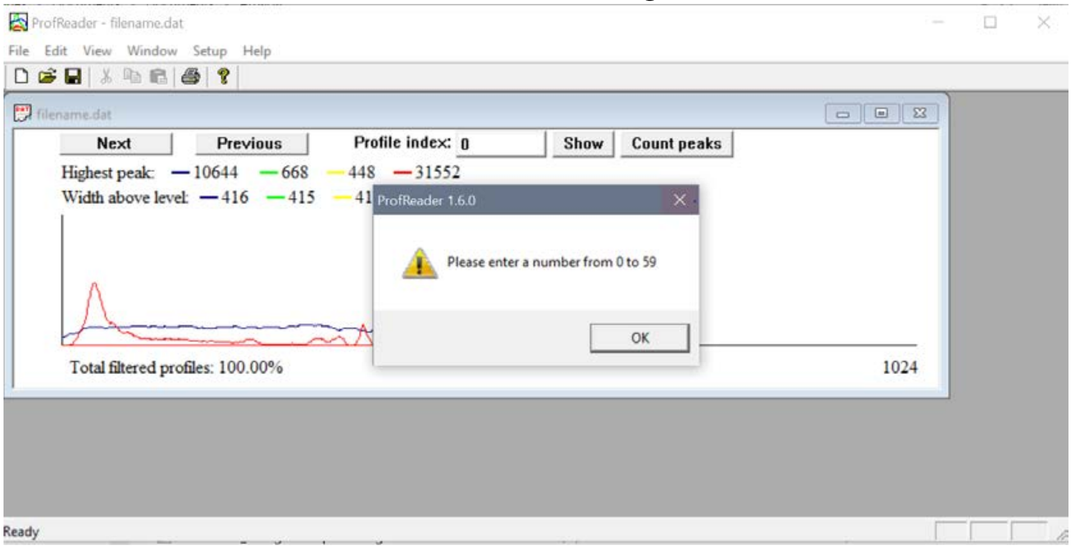

d. Go to File>Export as text

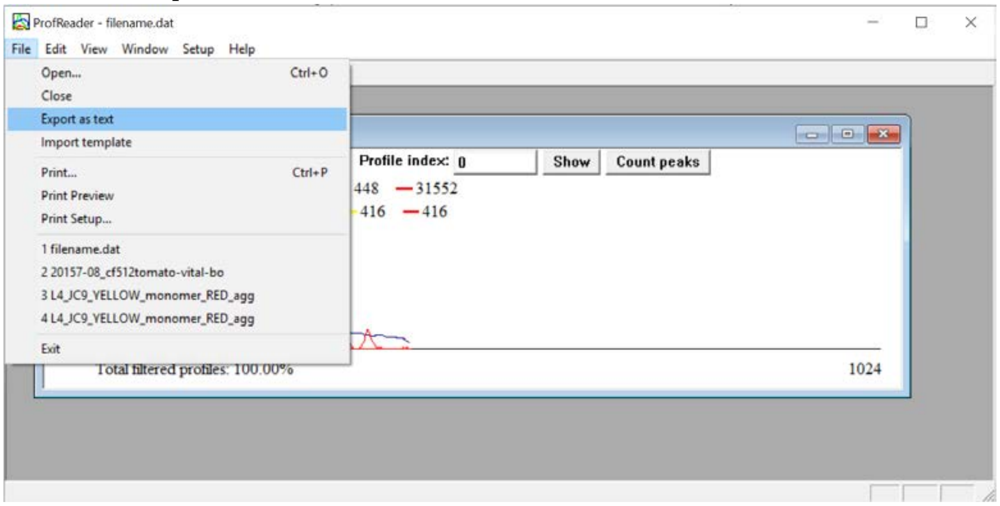

e. In the export data window, select the group of profiles to export (0-100, 0-200, etc.)

   i. Standard Window

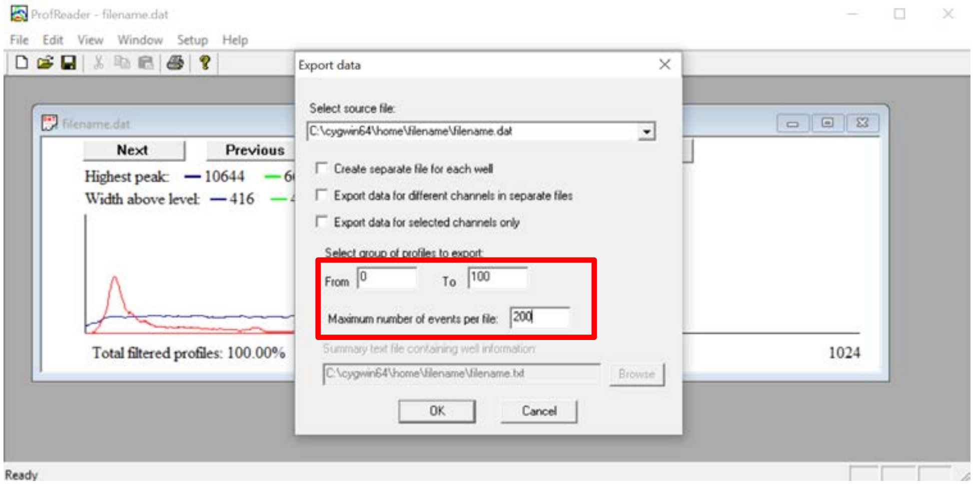

   ii. For the LAMPRO Suite, only these needed to be edited, as all data must be exported together, so ignore checkboxes.

      *Note: Maximum number of events per file must be higher than the number of profiles you are exporting.

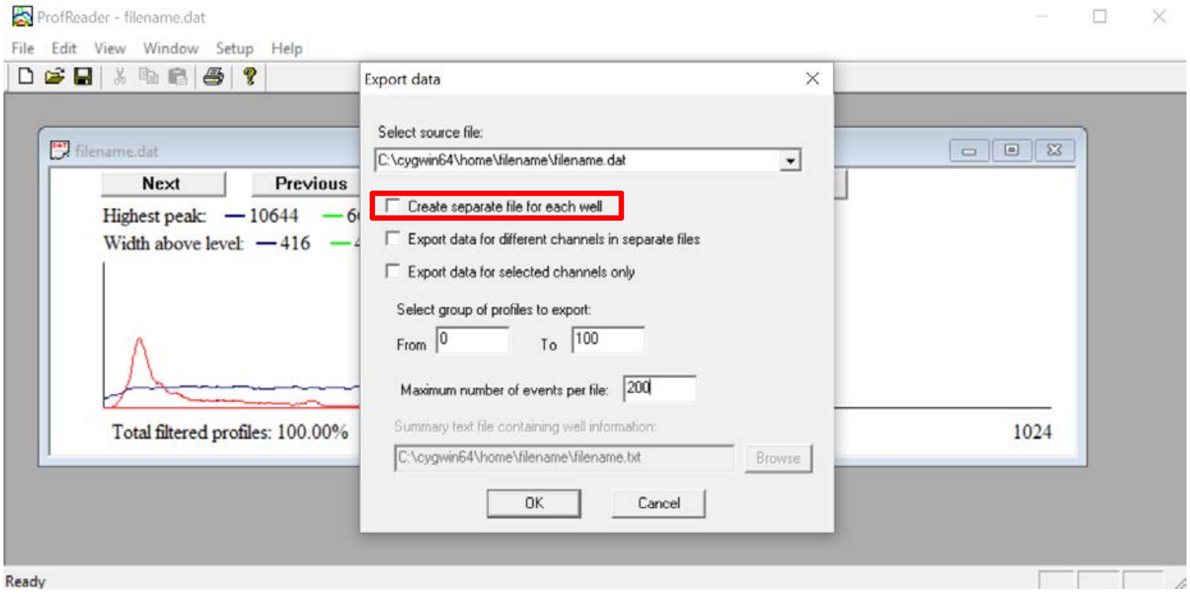

   iii. If the program is being run for well-specific data (a 96-well plate), check "Create separate file for each well". This prints all the profiles into separate textfiles based on the wells, for ease of use later

f. Ensure the maximum number of events per file is greater than the total number of exported profiles.
   i. For 0-334, # of events >= 335
   ii. 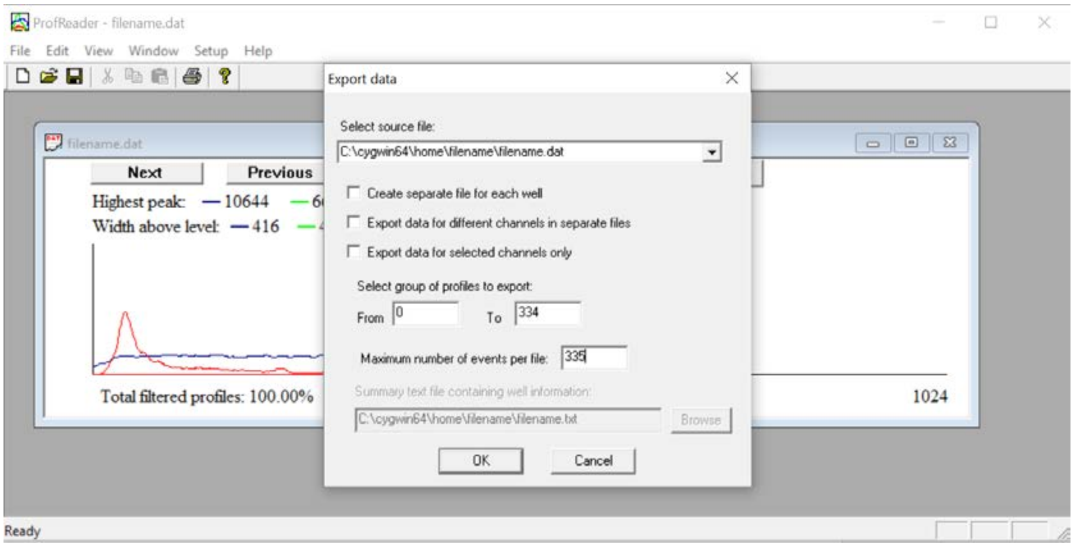

g. Hit okay, and label the new text file: "filename-profile_in.txt" with the desired filename in place of filename. "**–profile_in" MUST BE AT THE END, SO THAT METADATA IS NOT DELETED, AND THE PROGRAM CAN ACCESS THE DATA CORRECTLY.**

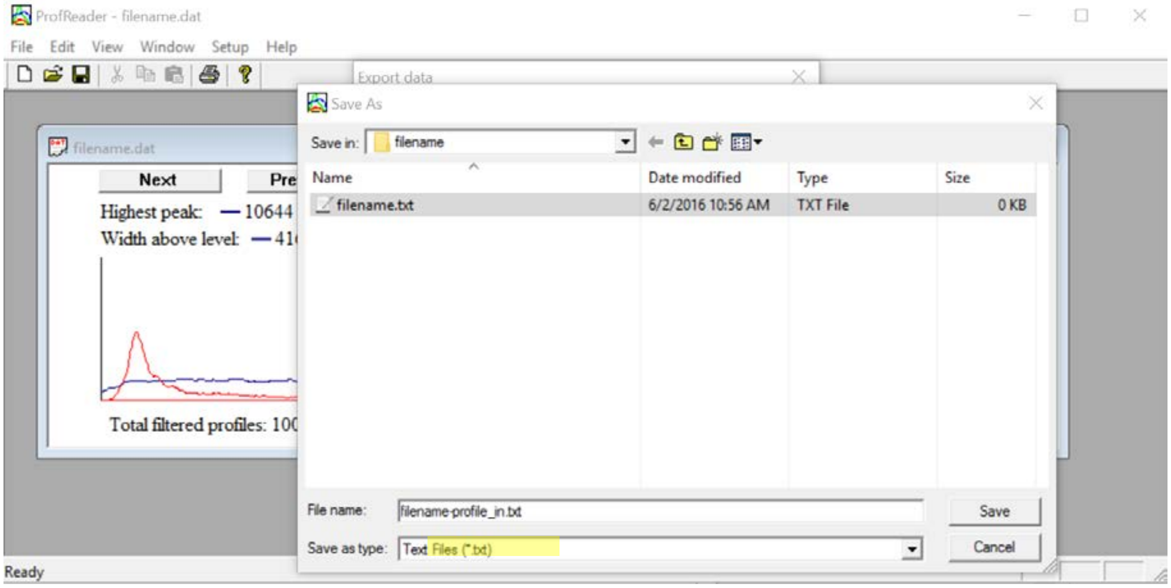

h. Save the file in the same folder as your profile data.

Cygwin setup: LAMPro is run through cygwin

3. Locating Files in Cygwin for use
    a. Open the cygwin terminal

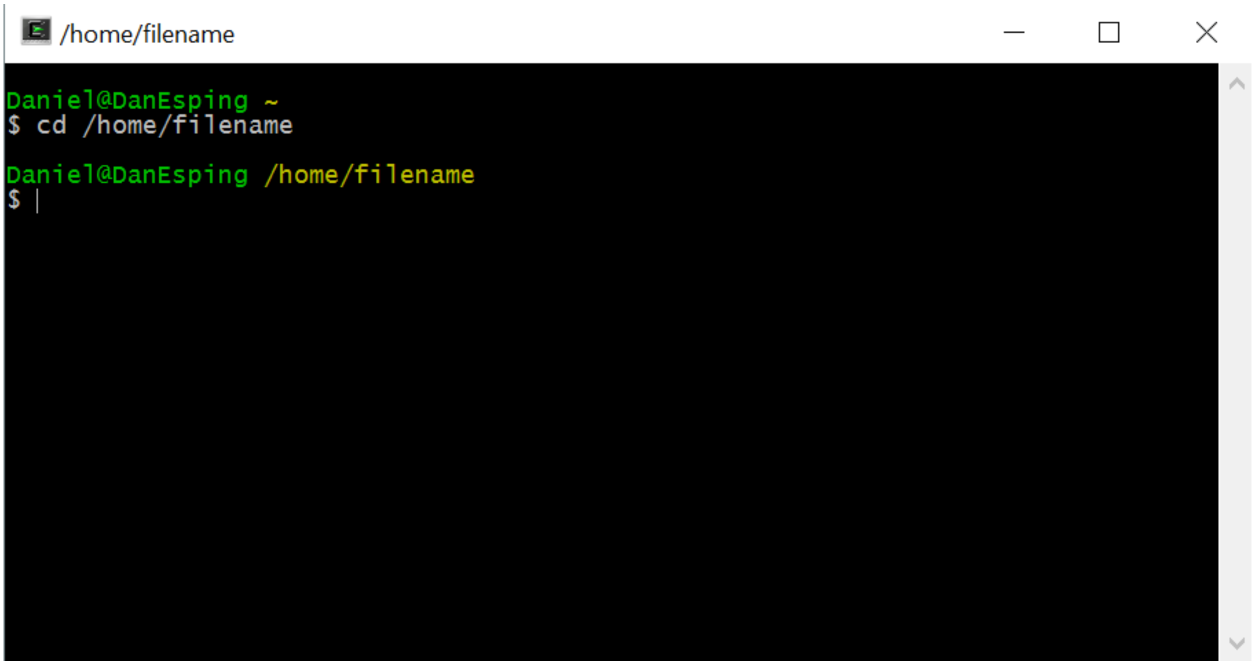

    b. Changing Directory/Cygwin Directory basics

Inputs	Windows Connection
/cygwin64/	64 bit Cygwin folder
/cygwin32/	32 bit Cygwin folder
~	C\cygwin64\home\Users Name
/	C\cygwin64
dir	List the directory
cd	Change directory
/cygdrive/	Base windows disk names. The directory of /cygdrive/ displays the C:// drive, D:// drive, etc.

4. Symbolic Link to correct Folder: For ease of use in executing the perl code, set up a symlink or shortcut from one folder to the folder with stored data
    a. Because of heavy data use, it could be useful to keep data in a D drive, etc, and use a symbolic link to get to the folder easily
    b. Locate the desired directory in your User directory

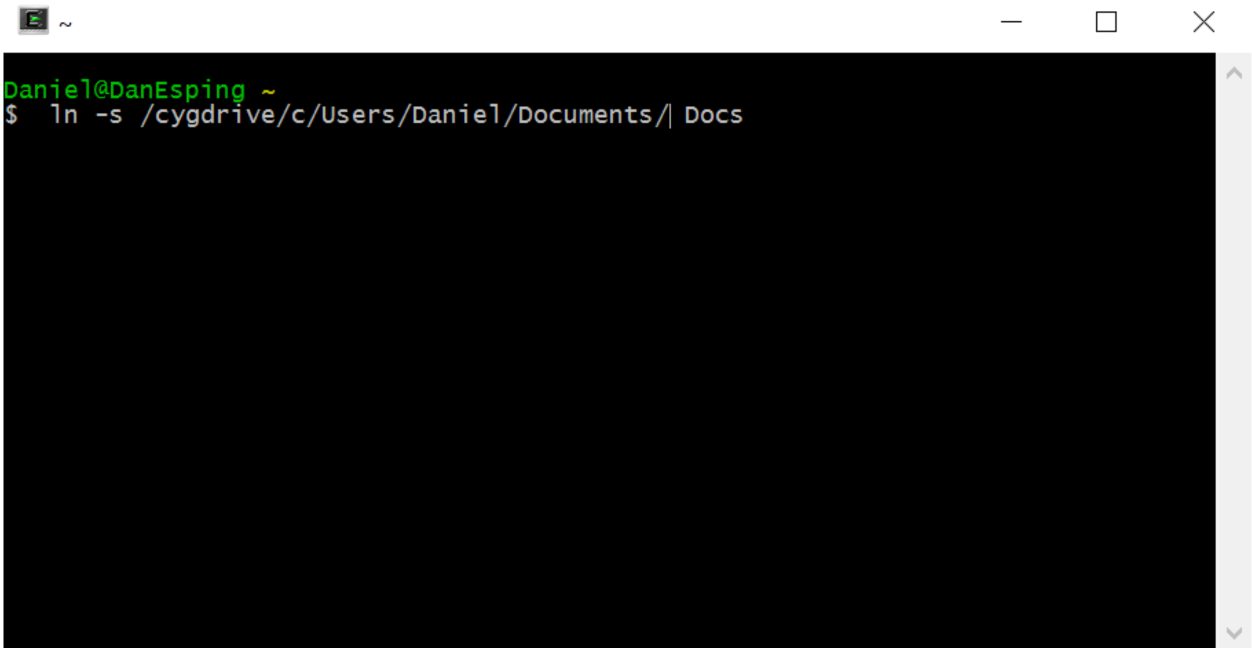

i. The name after the file location can be of your choosing
    1. This name will be your directory shortcut name, and can be accessed as shown below using the “cd” command
ii. Now, LAMPro can be run after opening the Cygwin folder. This is where the LAMPro suite should be stored

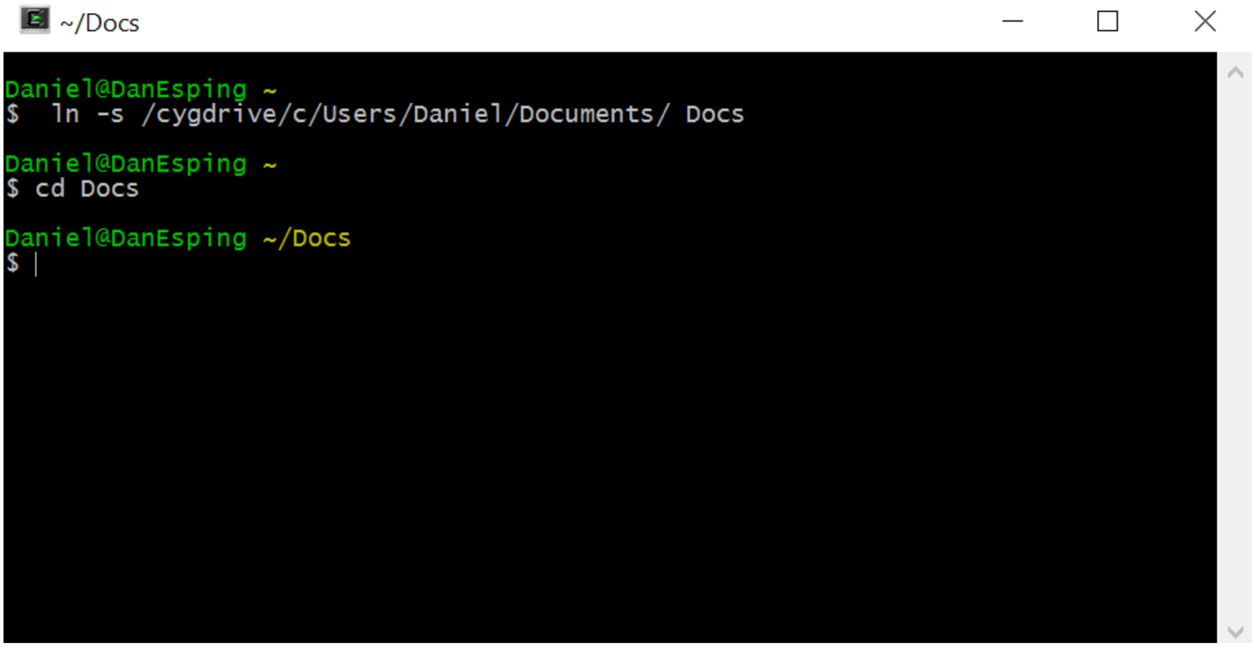

c. Now the system is ready to call the LAMPro program, assuming all files are saved in the correct folder (Docs in this example)

5. Running LAMPro alignment software

a. The LAMPro program syntax is: perl LAMPro.pl <filename> <orientation color> printtype <print color> exclusion <on/off> <type> &> <filename>-screen.txt, as shown below: (green is typed always, yellow is changeable from the below picture)

   i. Perl:
   ii. LAMPro.pl or LAMPro_wells.pls (if running the well-specific program for screens):
   iii. Printtype: indicates the print color being chosen
   iv. Exclusion: indicates the exclusion choice is being made
   v. &> filename-screen.txt: exports all printed errors and data to filename-screen.txt in the ~/Docs folder

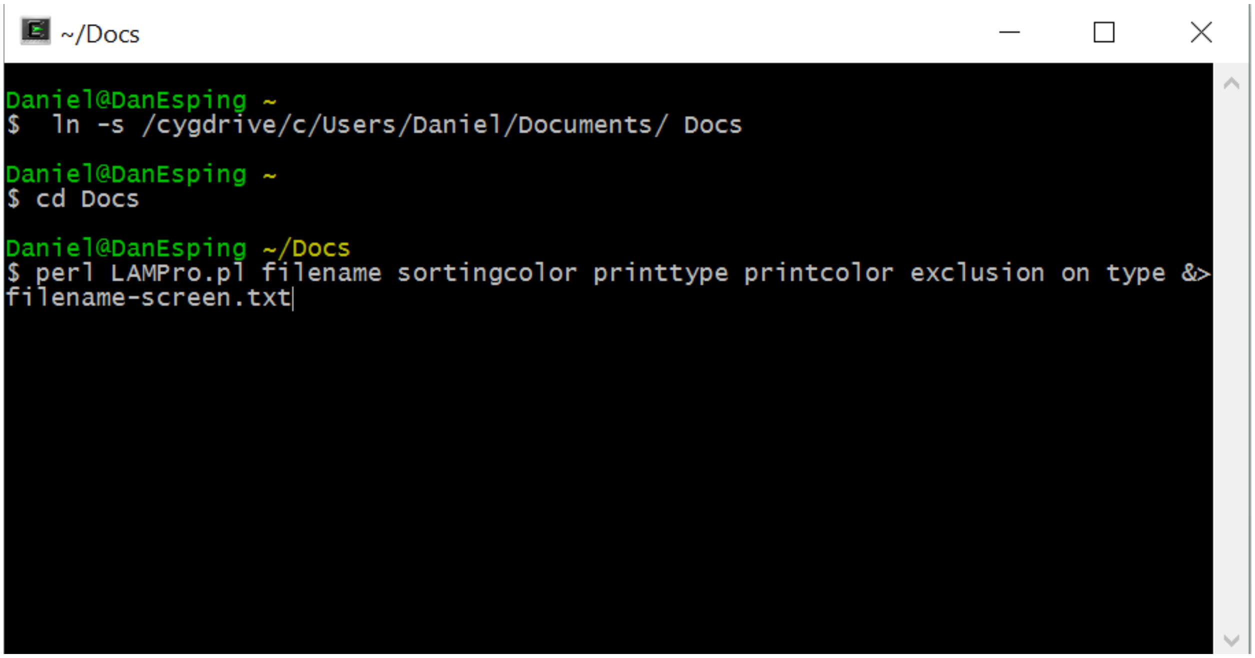

Changeable Text	Options	Explanation
Filename		Should not have a .txt at the end of it
Sortingcolor	yellow, red, green, ext	Desired fluorescence or extinction for sorting and orienting
Printcolor	yellow, red, green, ext, ratio (ratio = red/yellow fluorescence)	Desired fluorescence, extinction, or ratio to be printed
Exclusion Choice	On	Exclusion on will reduce the total number of profiles, but will increase the quality of the profiles
	Off	Exclusion off still imposes a TOF exclusion to ensure the profiles are large enough

Type		Specific commands that trigger specific exclusion, orienting, and printing instructions, to be used when certain protocols must be followed, or can be ignored
	Red, Yellow, Green, or EXT	Implements the exclusion criteria for the desired fluorescence or optical density<br>• The sorting color and printing color exclusion criteria will be automatically implemented, so any additional fluorescence/density specific criteria that should be enacted are used here
	ratio	Uses extra ratio exclusion criteria to reduce irregularities in the calculated ratio
	der	Implements the derivative orientation system for neuronal and pharynx-based orientation markers.<br>• What changes: Searches for a max value in the 20% position on the anterior or posterior of the profile
	mitoder	Implements the derivative orientation system for mitochondrial-based orientation markers, such as JC-9.<br>• What changes: Searches for a max value in the 40% position on the anterior or posterior of the profile
	noder	Ensures that only the Chi-Squared orientation system is used for the orientation of profiles, even if the derivative-specific system patterns are apparent in the data.
	DiO	Used when the sorting color is green and the DiO dye was used
	DiI	Used when sorting color is red and the DiI dye was used
	Mpo1a	Used when running zebrafish data.<br>• Changes the length of the profiles and tof requirements to better suit the larger organism
	Mosquito	Used when running Mosquito data. Same purpose as mpo1a, but for mosquito.

Dros	Used when running *Drosophila* data. Same purpose as mpo1a and mosquito

vi. <filename> should not have a .txt at the end of it

vii. <sortingcolor>: Desired fluorescence or extinction for sorting and orienting

  1. options: yellow, red, green, ext

viii. <printcolor>: Desired fluorescence, extinction, or ratio to be printed

  1. options: yellow, red, green, ext, ratio (ratio=red/yellow fluorescence)

ix. <on/off>: choose if the exclusion criteria is on or off

  1. Exclusion off still imposes at TOF exclusion to ensure the profiles are large enough
  2. Exclusion on will reduce the total number of profiles, but will increase the quality of the profiles

x. <type>: Specific commands that trigger specific exclusion, orienting, and printing instructions, to be used when certain protocals must be followed, or can be ignored

  1. Ratio: Uses extra ratio exclusion criteria to reduce irregularities in the calculated ratio
     a. Turns off the yellow fluorescence-specific exclusion criteria, as the ratio exclusion criteria overlaps
  2. Red, Yellow, Green, or EXT: Implements the exclusion criteria for the desired fluorescence or optical density
     a. The sorting color and printing color exclusion criteria will be automatically implemented, so any additional fluorescence/density specific criteria that should be enacted are used here
  3. Der: Implements the derivative orientation system for neuronal and pharynx-based orientation markers.
     a. What changes: Searches for a max value in the 20% position on the anterior or posterior of the profile
  4. Mitoder: Implements the derivative orientation system for mitochondrial-based orientation markers, such as JC-9.
     a. What changes: Searches for a max value in the 40% position on the anterior or posterior of the profile
  5. Noder: Ensures that only the Chi-Squared orientation system is used for the orientation of profiles, even if the derivative-specific system patterns are apparent in the data.
  6. dio: Used when the sorting color is green and the DiO dye was used
     a. eliminates profiles with DiO in the intestine of the worm
  7. dii: Used when sorting color is red and the DiI dye was used
  8. mpo1a: Used when running zebrafish data.
     a. Changes the length of the profiles and tof requirements to better suit the larger organism
  9. Mosquito: used when running Mosquito data. Same purpose as mpo1a, but for mosquito.
  10. Dros: used when running *Drosophila* data.

Example LAMPro.pl command and Explanation

perl LAMPro.pl hsp6pGFP_EV_rnai_GREEN_DiI_RED red printtype green exclusion on der>hsp6pGFP_EV_rnai_GREEN_DiI_RED-screen.txt

perl LAMPro.pl - calls the program

hsp6pGFP_EV_rnai_GREEN_DiI_RED – specifies which folder to look into

red – specifies the program to “orient” using the “red” channel

printtype green – tells the program to “print”/output “profiles” from the “green” channel after orientation

exclusion on der - tells the program to utilize our exclusion criteria (TOF, basal fluorescence, extinction) and to utilize the “derivative” test to orient profiles

>hsp6pGFP_EV_rnai_GREEN_DiI_RED-screen.txt – tells the program what to call the screen file (if any errors occurred while running) which will be printed when the program finishes running

LAMPro GUI DIRECTIONS

Step-By-Step Directions for Running the LAMPro GUI (Data Visualization Suite)

SETUP

1. To install the LAMPro GUI Suite Application, first download the LAMPro_UI.mlappinstall file.

2. Open MATLAB, and go to the APPS Tab at the top. Click on the Install App Button.

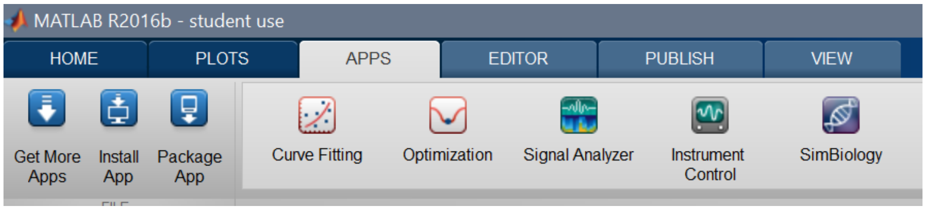

3. Navigate to where the LAMPro_UI App was downloaded, and click to choose the correct file.

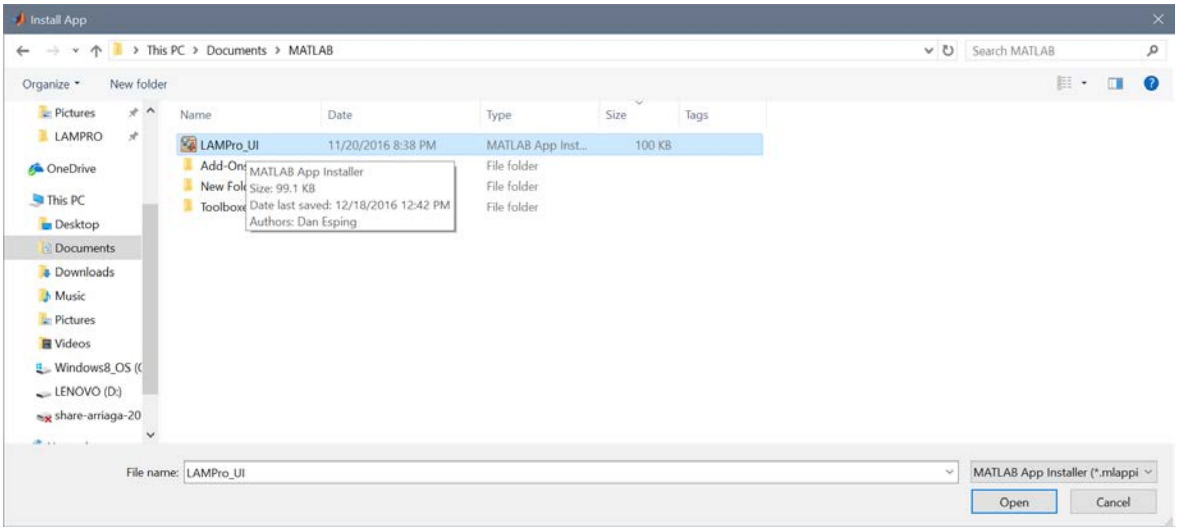

4. Agree to Install the Application into MATLAB.

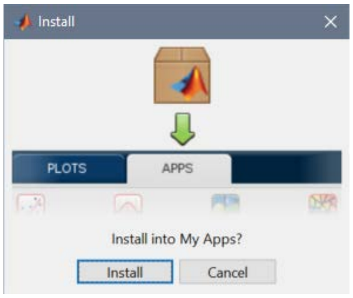

If attempting to update the application, an update confirmation box will appear. Choose Update to replace old version of the program with the new version.

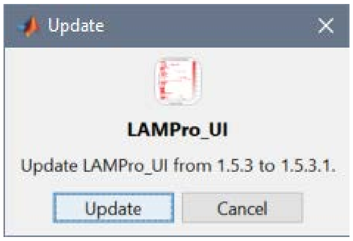

5. See the LAMPro_UI Application is now displayed in your list of APPS in the APPS Tab. Double Click the App to open

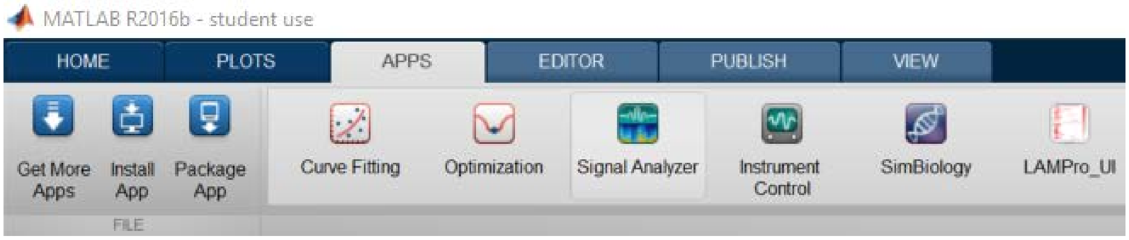

Hovering over the icon in the APP Tab will display version number, MATLAB Required Toolboxes, and File Location of the Application

LAMPro GUI

Once the LAMPro Program has been opened, the LAMPro GUI will appear.

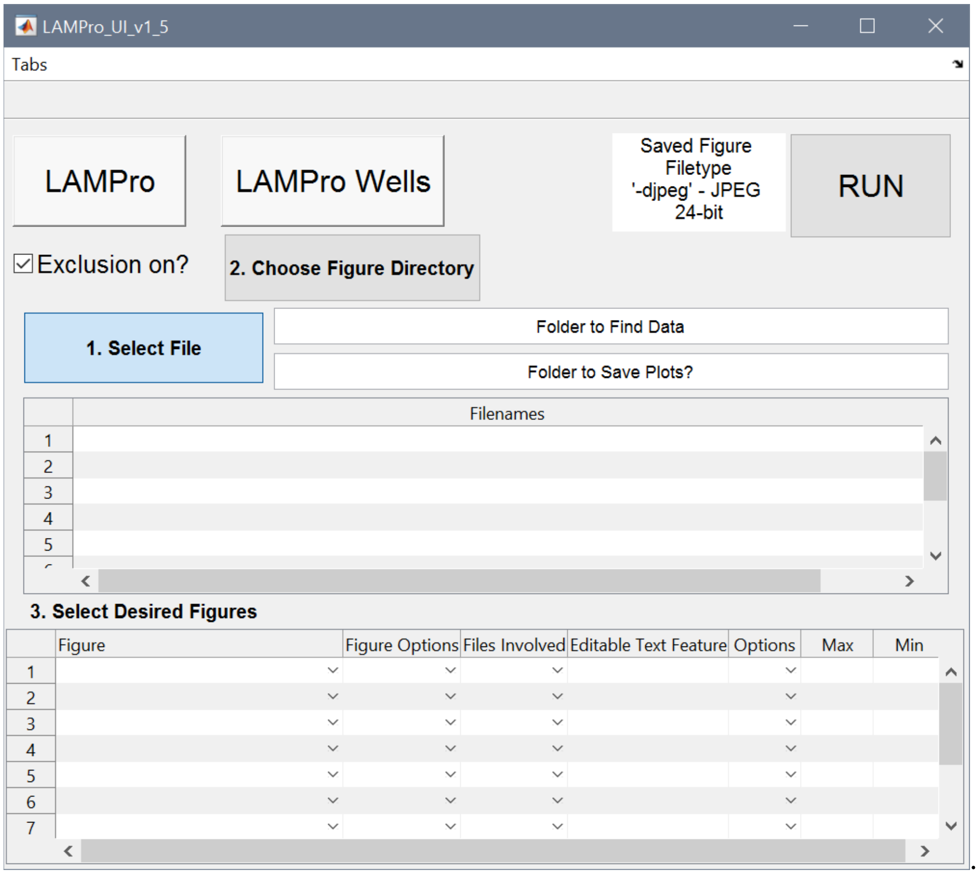

The GUI is automatically set to run for the LAMPro setup, and can be changed to the LAMPro Wells setup by toggling tabs, or clicking the LAMPro Wells button. The GUI also automatically chooses to use data from when the exclusion criteria was used (Exclusion on). By clicking the checkmark, this can be set to exclusion off, where the data being used will not use most exclusion criteria (Exclusion off).

1. Select File
   - Click the Select File Button to select a file that displays the filenames included in the LAMPro GUI run.

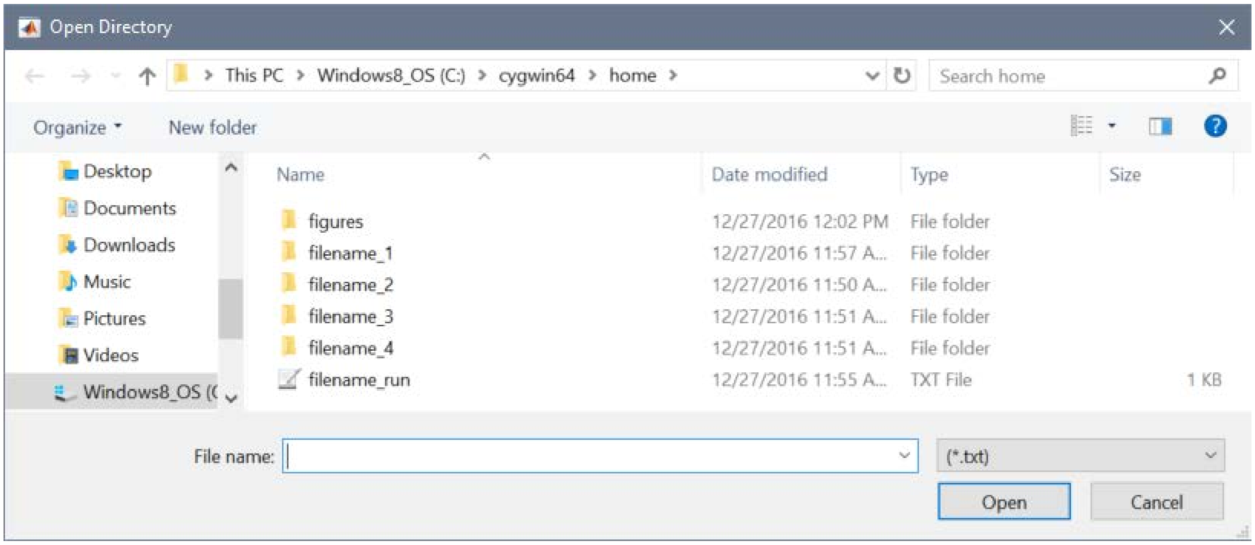

   - This filename_run.txt displays the filenames in succession that will be ran through the GUI, as shown below. The name of the file can vary, but the filename_run text file must be saved in the same directory as the files folders it displays (filename_1 through filename_4 can be seen in the same folder).

   - Note: The filename_run.txt file contains filenames that do not end in .txt; only the filename should be displayed.

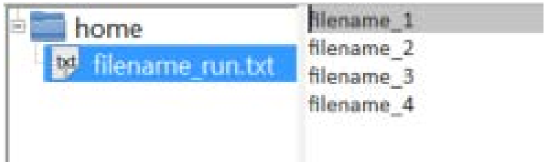

Once filename_run.txt has been chosen, the files included in the text file will be displayed in the filenames field, and the directory will be populated in the text field that displayed "Folder to Find Data."

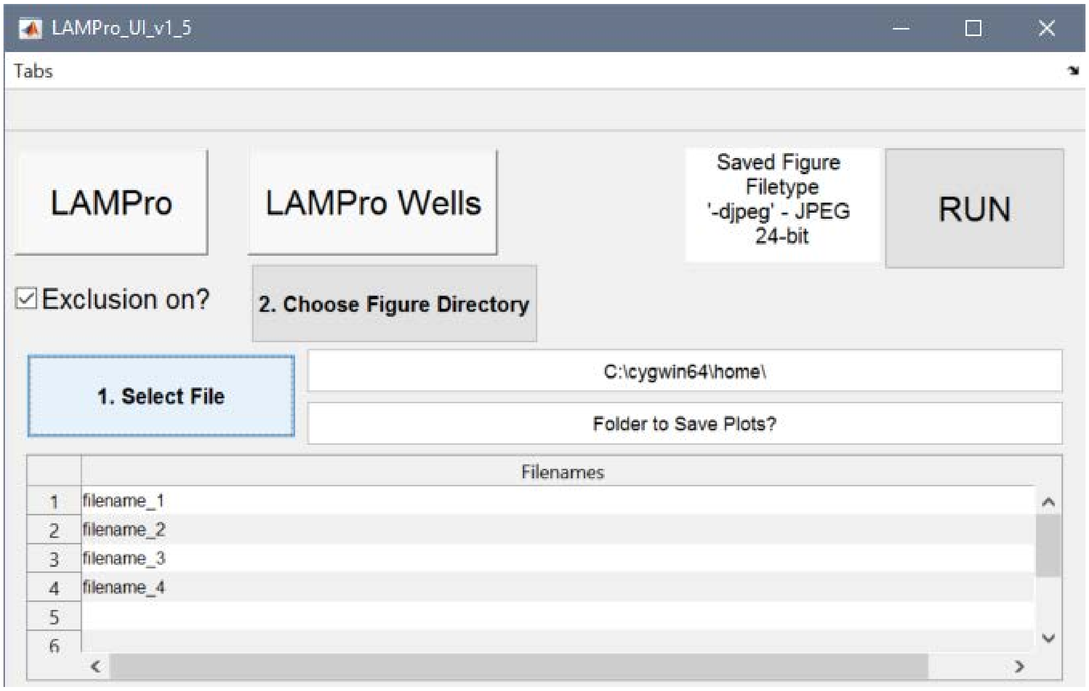

2. Choose Figure Directory

- Click "Choose a Figure Directory" so the figures that will be generated by the GUI will be stored.

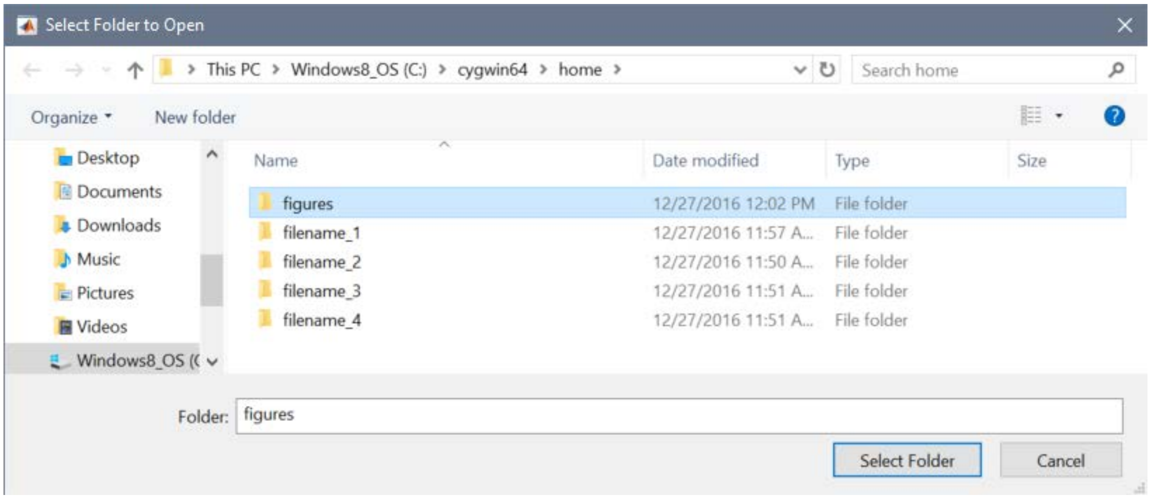

- Note: The folder where the figures will be stored does not need to be in the same folder as where the data is stored, but should be in the same drive (C:\).

- The LAMPro GUI will save the figures in the format of …figures/date/filename_run/exclusion_on/, where "figures" is the selected folder above, date is saved DD-Month-YYYY, filename_run is the text file that displays filenames for the GUI to run, and exclusion_on is the desired exclusion choice, either exclusion_on or exclusion_off, depending on your choice.

Once the figure directory is chosen, the GUI will populate the "Folder to Save Plots?" text field with the desired directory.

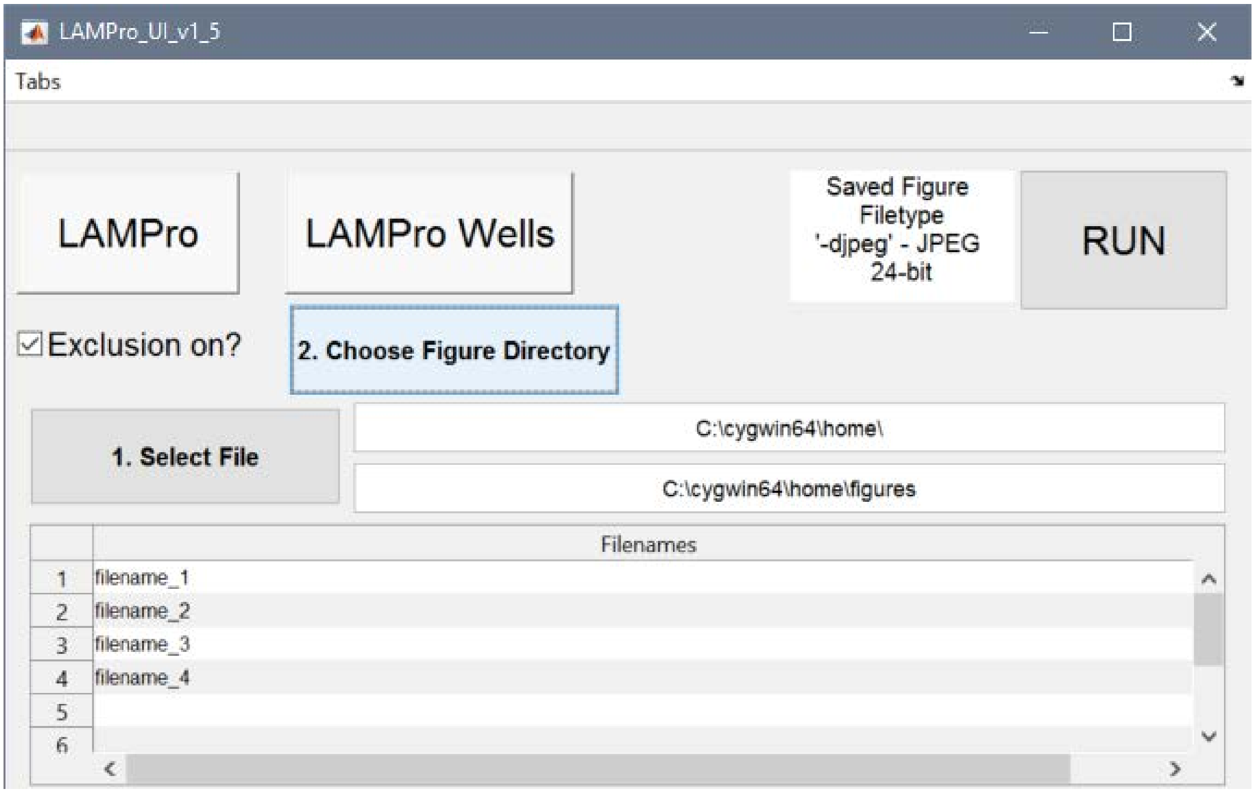

3. Select Desired Figures

- Below is the Desired Figures Table filled with many of the common figures that were analyzed. Based on the chosen figure, different analytical programs will be executed from the ***LAMPro Suite*** to visualize the desired data from the perl LAMPro Program This figure will be referenced by the figure line denoted on the left side of the table.

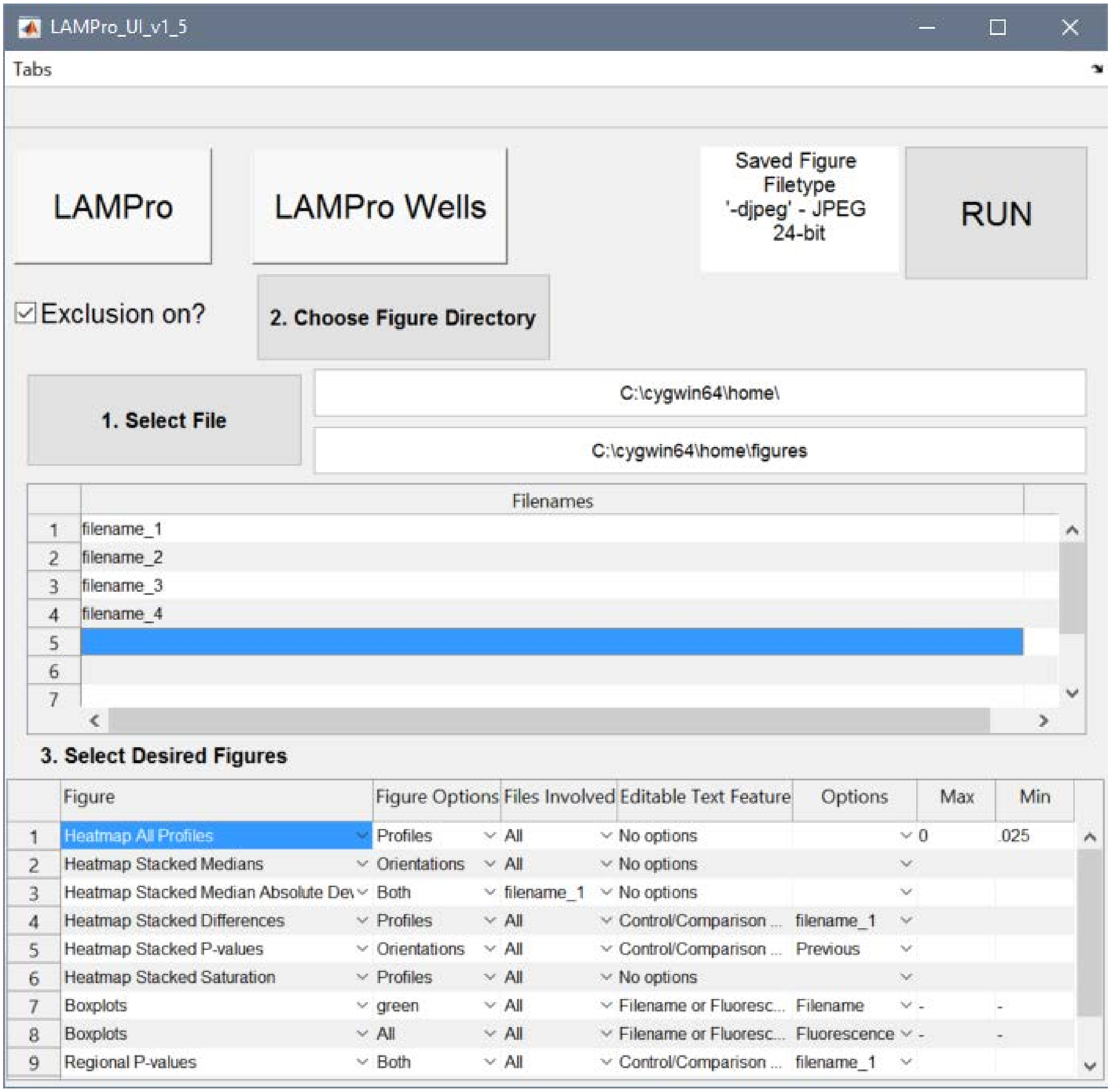

- From the displayed list of Figures, there are a few different types of figures that all use the Same Figure Options, Files Involved and Options Menus

A. *Standard Figure* Type - **Heatmap All Profiles**, **Heatmap Stacked Medians** and **Heatmap Stacked Median Absolute Deviation (MAD)**: The **Heatmap All Profiles** plot prints a separate figure for each filename chosen, where all profiles saved are stacked vertically and displayed. The **Heatmap Stacked Medians** plots the median of each longitudinal value for all the worms within a file as a function of the percentage length along the worm. The variability of this signal is

plotted using median absolute deviation (MAD).

- Figure Options: Choice of using Profiles, Orientations, or Both, where profile is the desired data executed in LAMPro(ratio for JC-9 data), orientation is the signal that was used for orienting the data in LAMPro (yellow for JC-9 data), and both prints figures for both profile and orientation data sets on separate figures.

- Files Involved: The option of choosing one of the four files displayed above, (filename_1 in line 3 above), or choosing All, which will find and print all the four files' data on the same figure.

- Options: There are no additional Options for this figure type

- Min/Max: For the **Heatmap Stacked Medians** plot, there is a special Normalization feature that allows the signal to be normalized to the maximum value in each median profile. This feature was used to distinguish the longitudinal sections that the tissues exist in, such as where the pharynx signal ends and where the neuronal signal peaks exist. To use the Normalization feature, "max" is written into the Max column. The maximum signal for the colormap scale will equal 1. In addition, typing "min" into the Min column will change the colormap scale so that the smallest value in the colormap will equal the minimum signal value for all filenames (the minimum of filename_1, filename_2, filename_3, and filename_4, combined). If both max and min are typed into the Max and Min columns, the plots will most likely have a colormap ranging with bounds of 1 and 0 (assuming one of the plotted median profiles has a value of 0 so that the minimum is 0). Each median profile will show heatmaps normalized to allow for better understanding of how the signal changes with respect to the longitudinal position.

- Examples: **Heatmap All Profiles**, shown in figure line 1, displays every profile for each filename as a heatmap, stacked vertically, with different figures for each of the four filenames. **Heatmap Stacked Medians**, shown in figure line 2, displays the median orientation heatmap for each of the four filenames, stacked vertically on the same figure. **Heatmap Stacked Differences**, shown in figure line 3, displays the Median Absolute Deviation (MAD) profile as a heatmap, with only the profile from filename_1 displayed.

B. *Comparison Figure* Type **- Heatmap Stacked Differences** and **Heatmap Stacked P-values**: The **Heatmap Stacked Difference** plots compares two median plots were by taking the difference between the median longitudinal value for each point along the length of each worm and then plotting this difference (Δ) longitudinally. For the **Heatmap Stacked P-values** plot, A Wilcoxon Rank Sum Test was calculated at each longitudinal point along the length of the worm, using all profiles' values at that location as the data set. Degree of significance was plotted such that all *P-values* ≤ 0.05 were plotted as numbers > "0". More specifically, "$-1.3-\log_{10}(P\text{-}value)$" was plotted which distinguished regions of significance as above "0" ($P \leq 0.05$) and regions that were not significant ($P > 0.05$) as below "0"

- Figure Options: Choice of using Profiles, Orientations, or Both, with the same setup as the standard figure type

- Files Involved: The option of choosing one of the four files or choosing All applies to this figure type, as well.

- Options **-** Control/Comparison File: One of the example's four files will be compared against the other files listed in Files Involved.

- Examples: Selecting filename_1 as in figure line 4 will compare filename_2 through filename_4 to filename 1, where the difference plot will subtract filename_1 from filename_2, etc. If only filename_2 is selected in the Files Involved field, then the plot will only display the difference between filename_2 and filename_1.

C. *Data Validation* Figure Type – **Heatmap Stacked Saturation**: The **Heatmap Stacked Saturation** plot ensures that the longitudinal signal isn't at the top threshold for signal by calculating the percentage of profiles that are at the maximum signal value at each longitudinal position. If 10 of 100 profiles are max

- Figure Options: Choice of using Profiles, Orientations, or Both, with the same setup as the standard figure type

- Files Involved: The option of choosing one of the four files or choosing All applies to this figure type, as well.

- Options: There are no additional Options for this figure type

- Examples: Figure line 6 will print a heatmap for each of the filenames, where the intensity of the heatmap is based on the number of profiles that have a maximum value at that longitudinal position.

D. *Summary Figure* Type – **Boxplots**: The **Boxplots** plot the median signal values for each profile in the desired file using a Tukey boxplot.

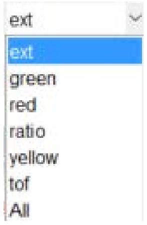

- Figure Options: Choice of optical thickness or extinction (ext), fluorescence signal (green, red, or yellow), ratio (red/yellow ratio, for JC-9 data), Time of Flight or length of the profile (tof), or to print all of the above signals.

- Files Involved: The option of choosing one of the four files or choosing All applies to this figure type, as well.

- Options - Filename or Fluorescence: Filename or Fluorescence dictates how the data is displayed in the figures. If Filename is chosen, the boxplots for each desired signal chosen in Figure Options will be stacked vertically and displayed on the same figure for each of the files chosen in Files Involved, with different figures for each file involved. If Fluorescence is chosen, the boxplots for each of the files chosen in Files Involved is stacked on top of each other and shown on the same figure for each signal chosen in Figure Options.

- Examples:

  1. Filename - If green is chosen for Figure Options, and all is chosen for Files Involved, as in the figure line 7 above, there will be a separate figure for each of the four files involved, with only a green fluorescence boxplot displayed on each figure.

  2. Fluorescence - If all is chosen for Figure Options and Files Involved, as in the figure line 8 above, then there will be separate boxplot figures for ext, green, red, yellow, ratio, and tof signals, with all four files involved displayed as separate boxplots, stacked vertically on the same figure for each of the signals.

E. *Regional Comparison Figure* Type - **Regional P-values**: The **Regional P-values** uses data

from the significance function of **Heatmap Stacked P-values** in the *Comparison Figure* Type. The profiles of the animal are split into anterior, middle, and posterior sections, denoted by the longitudinal percentage ranges of 0-25%, 25-85%, and 85-100%, respectively. In each longitudinal section, the frequency of *P-values* of differing significance ($P < 0.0001$, 0.001, 0.01, 0.05, and not significant, N.S.) within the set region of the animal can be plotted in a stacked histogram.

- Figure Options: Choice of using Profiles, Orientations, or Both, with the same setup as the standard figure type

- Files Involved: The option of choosing one of the four files or choosing All applies to this figure type, as well.

- Options - Control/Comparison File: One of the example's four files will be compared against the other files in Files Involved. For the figure line 9 above, filename_2 through filename_4 will be compared against filename_1 for both the Profile and Orientation datasets. If 70% of the *P-values* in the anterior of the filename_2 vs filename_1 profile are significant for $P < 0.0001$, and the remainder of the longitudinal *P-values* are significant for $P <.01$, the histogram will display 70% $P < .0001$ and 30% $P < .01$. Similar statistics would be found for the comparisons of filename_3 and filename_4 to filename_1, and then separate figures would be printed for the anterior, middle, and posterior sections, with the datasets from each filename stacked horizontally on the same plot.

LAMPro WELLS GUI

1. Select File
   - Click the LAMPro Wells button to go to the LAMPro Wells Tab, then click the Select File button. Navigate to the desired folder and click the text file that contains only the “base” name scheme of the files desired. This file is printed by the COPAS Instrument and contains basic information that will be utilized in the program.

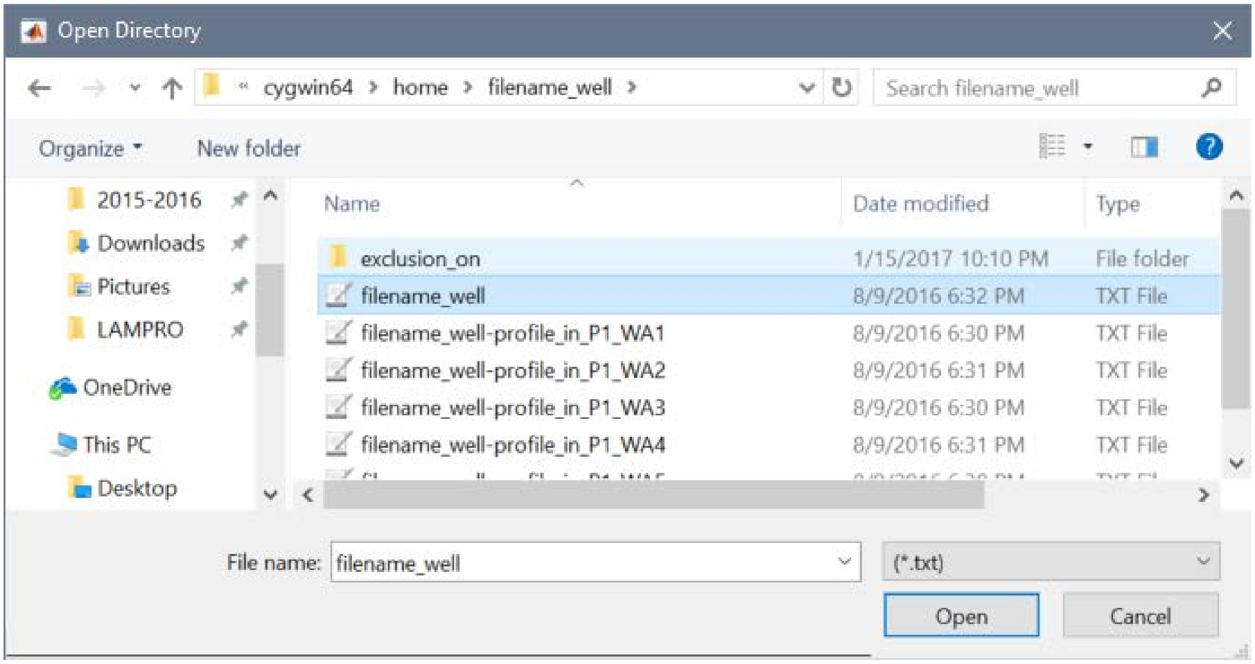

2. Choose Figure Directory
   - Now that the file is chosen, the folder for the saved figures is selected. This step operates the same as the LAMPro GUI.

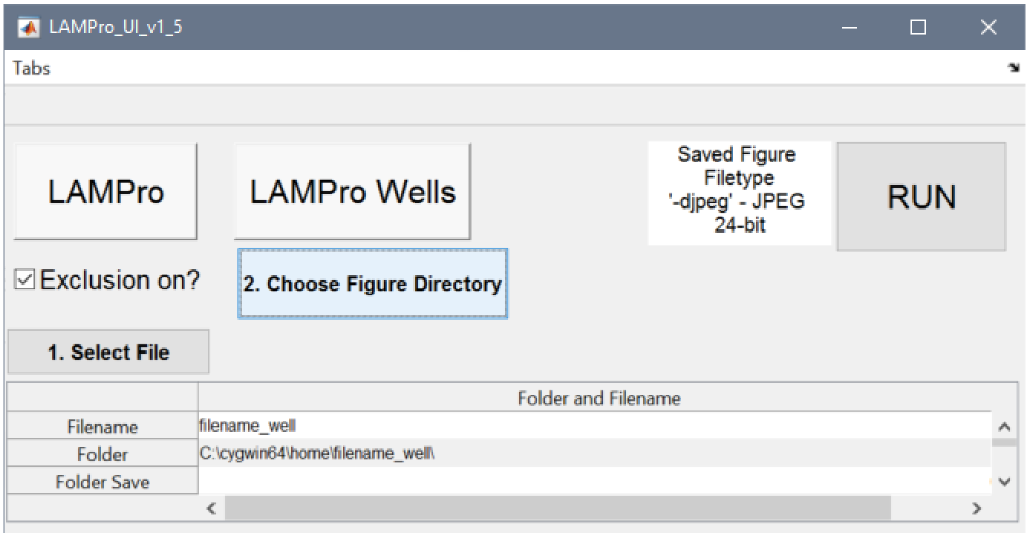

3. Select Desired Figures

- Below is the Desired Figures Table filled with common figures that were analyzed. Based on the chosen figure, different analytical programs will be executed from the LAMPro Wells part of the ***LAMPro Suite*** to attain the desired data from the perl LAMPro Wells Program This figure will be referenced by the figure line denoted on the left side of the table.

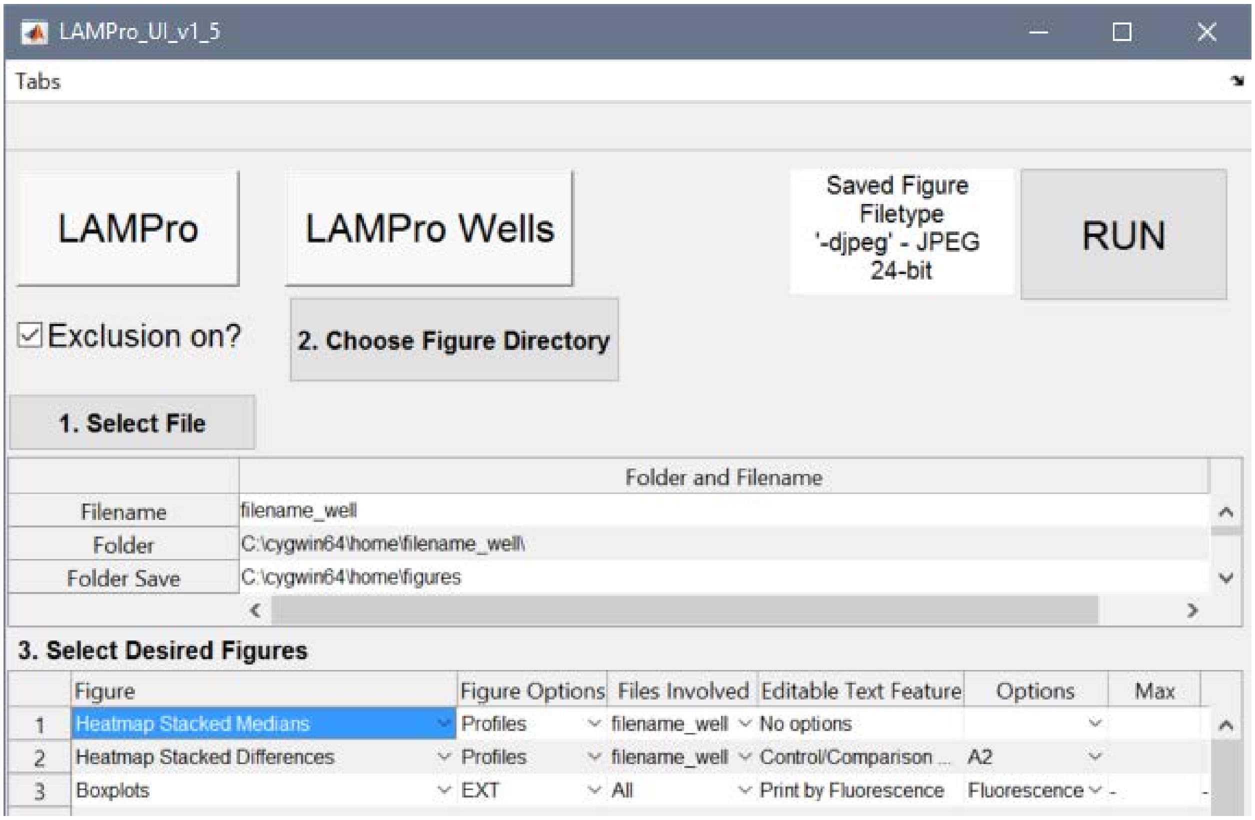

A. *Standard Figure* Type **- Heatmap Medians**: The **Heatmap Stacked Medians** plots the median of each longitudinal value for all the worms within a file as a function of the percentage length along the worm.

- Figure Options: Choice of using Profiles, Orientations, or Both, where profile is the desired data executed in LAMPro (ratio for JC-9 data), orientation is the signal that was used for orienting the data in LAMPro (yellow for JC-9 data), and both prints figures for both profile and orientation data sets on separate figures.

- Files Involved: The option of choosing All or the Filename displayed above both give the same data from the Filename Displayed.

- Options: There are no additional Options for this figure type

- Examples: **Heatmap Stacked Medians**, shown in figure line 1, displays the median heatmap of the profiles for each well, stacked vertically on the same figure.

B. *Comparison Figure* Type – **Heatmap Stacked Differences**: The **Heatmap Stacked**

Difference plots compares two median plots were by taking the difference between the median longitudinal value for each point along the length of each worm and then plotting this difference (Δ) longitudinally.

- Figure Options: Only data from profiles is allowed on this figure.

- Files Involved: The option of choosing All or the Filename displayed above both give the same data from the Filename Displayed.

- Options - Control/Comparison File: A dropdown of all wells allows for one well to be selected as the comparison well for the other wells to be compared against.

  Note: This chosen well must have the correct data stored. If a well is chosen and the program cannot find the data, an error will occur.

- Examples: Figure line 2 displays stacked longitudinal heatmaps for each of the wells, excluding well A2, for the difference between that well and A2, at each longitudinal position.

C. *Summary Figure* Type – **Boxplots**: The **Boxplots** plot the median signal values for each profile in the desired file using a Tukey boxplot.

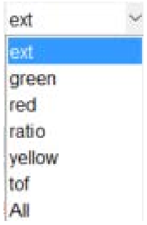

- Figure Options: Choice of optical thickness or extinction (ext), fluorescence signal (green, red, or yellow), ratio (red/yellow ratio, for JC-9 data), Time of Flight or length of the profile (tof), or to print all the above signals.

- Files Involved: The option of choosing All or the Filename displayed above both give the same data from the Filename Displayed.

- Options - Fluorescence: Fluorescence dictates how the data is displayed in the figures. As Fluorescence is chosen as the only option, the boxplots for each of the wells is stacked on top of each other and shown on the same figure for each signal chosen in Figure Options.

- Examples:

  1. Fluorescence – If EXT is chosen for Figure Options, as in the figure line 3 above, then there will be one boxplot figures for the EXT signals, with all wells displayed as separate boxplots, stacked vertically on the same figure. Additional figures would be created for each additional signal if not only EXT was selected.
